# Sequence-Based Prediction of Type III Secreted Proteins

**DOI:** 10.1371/journal.ppat.1000376

**Published:** 2009-04-24

**Authors:** Roland Arnold, Stefan Brandmaier, Frederick Kleine, Patrick Tischler, Eva Heinz, Sebastian Behrens, Antti Niinikoski, Hans-Werner Mewes, Matthias Horn, Thomas Rattei

**Affiliations:** 1 Technische Universität München, Department of Genome Oriented Bioinformatics, Wissenschaftszentrum Weihenstephan, Freising, Germany; 2 Institute for Bioinformatics and Systems Biology (MIPS), Helmholtz Zentrum München, German Research Center for Environmental Health (GmbH), Neuherberg, Germany; 3 University of Vienna, Department of Microbial Ecology, Vienna, Austria; The Rockefeller University, United States of America

## Abstract

The type III secretion system (TTSS) is a key mechanism for host cell interaction used by a variety of bacterial pathogens and symbionts of plants and animals including humans. The TTSS represents a molecular syringe with which the bacteria deliver effector proteins directly into the host cell cytosol. Despite the importance of the TTSS for bacterial pathogenesis, recognition and targeting of type III secreted proteins has up until now been poorly understood. Several hypotheses are discussed, including an mRNA-based signal, a chaperon-mediated process, or an N-terminal signal peptide. In this study, we systematically analyzed the amino acid composition and secondary structure of N-termini of 100 experimentally verified effector proteins. Based on this, we developed a machine-learning approach for the prediction of TTSS effector proteins, taking into account N-terminal sequence features such as frequencies of amino acids, short peptides, or residues with certain physico-chemical properties. The resulting computational model revealed a strong type III secretion signal in the N-terminus that can be used to detect effectors with sensitivity of ∼71% and selectivity of ∼85%. This signal seems to be taxonomically universal and conserved among animal pathogens and plant symbionts, since we could successfully detect effector proteins if the respective group was excluded from training. The application of our prediction approach to 739 complete bacterial and archaeal genome sequences resulted in the identification of between 0% and 12% putative TTSS effector proteins. Comparison of effector proteins with orthologs that are not secreted by the TTSS showed no clear pattern of signal acquisition by fusion, suggesting convergent evolutionary processes shaping the type III secretion signal. The newly developed program EffectiveT3 (http://www.chlamydiaedb.org) is the first universal in silico prediction program for the identification of novel TTSS effectors. Our findings will facilitate further studies on and improve our understanding of type III secretion and its role in pathogen–host interactions.

## Author Summary

Many Gram-negative bacteria live closely associated with humans, animals, or plants. The pathogenic or symbiotic interactions between bacteria and host are often mediated by the secretion of bacterial proteins into the host cells. The Type III secretion system (TTSS) is one of the best studied cellular machineries for this purpose and is able to specifically recognize and export effector proteins, which are injected into the eukaryotic cells through a needle-like structure. However, neither the mechanism of transport nor the recognition of proteins to be exported via the TTSS has so far been fully comprehended. In this study we have developed the first general computational model that is able to identify TTSS effector proteins based on the analysis of a short part of their amino acid sequences. The features of this signal sequence are universal among human and animal pathogens and plant symbionts. Based on our findings, we developed a computer program for the in silico prediction of TTSS effector candidates; for example, in new genomes. The TTSS and its effector proteins constitute a central virulence mechanism of several bacterial pathogens responsible for severe and widespread infectious diseases in humans and animals. Our findings will facilitate and improve further investigations of TTSS-mediated pathogenesis and its role in pathogen–host interactions.

## Introduction

Many Gram-negative bacteria with symbiotic or parasitic lifestyles modulate their environment, the eukaryotic host cell, by the secretion of bacterial proteins into the host cell through the type III secretion system (TTSS) [1]. The unique role of type III mediated transport for establishing as well as maintaining infection makes it a key mechanism for bacterial pathogenesis [2]–[4]. While much progress on resolving the structure of the TTSS itself has been made recently [5], the identity and function of only few effector proteins is so far understood well. These include different virulence factors, which interact with cell signaling pathways to suppress immune response by inducing apoptosis in macrophages as the *Yersina* effector YopJ or the *Salmonella* effector SipB [6],[7]. Other known effectors manipulate the cytosceleton by actin re-arrangements as described for the *Salmonella* effector SipA [8]. The arsenal of known effectors varies widely between different bacterial species due to adaptation to different hosts and different survival strategies [9] and even between different strains of the same organism as shown for *Pseudomonas syringae* [10].

Experimental identification of novel effectors relies on translocation assays using fusion proteins of a putative effector with a reporter gene [11]–[14] or detection of effectors in the culture supernatant [11]. In many of these studies, prior information is derived computationally from the genome or from protein sequences to create candidate lists of putative effectors before testing them in an appropriate assay. Homology to known effector proteins has been used in a screen for effectors in the pathogenic *Escherichia coli* strain O157 [11]. Chromosomal co-localization of putative effectors with TTSS related chaperons has been used in *Bordetella bronchiseptica* [15]. Common transcriptional regulation with elements of the TTSS has been exploited to detect putative effectors in *P. syringae* [13],[16]. In the same organism, an unusual amino acid composition in the N-termini of effectors has been identified as a characteristic of effector proteins and used for their identification [16]–[18].

In all these approaches, the computational analysis successfully limited the amount of candidates which had to be included in experimental analyses in order to find novel effectors. However, none of these methods is either exhaustive or generally applicable. Homology based approaches can only detect effectors which are members of known effector families, and these are mostly specific for certain well-known bacterial species. Approaches using transcriptional co-regulation need knowledge about a TTSS effector specific promoter which has not yet been described for most bacteria possessing a TTSS. The unusual amino acid composition in the effector N-termini has to date only been described and exploited in screens in *P. syringae*. Chromosomal co-localization is only applicable if effectors and TTSS related proteins or chaperones are clustered in genomic proximity as described for the pathogenicity islands in *Salmonella* [19]. However, these pathogenicity islands are absent in other bacteria known to harbour a TTSS such as the *Chlamydiae*, where the genes encoding known effectors are scattered around the genome [20],[21].

In order to create a general method for the prediction of type III secreted proteins, the most straightforward way would be the identification of a general molecular signal which leads to specific recognition of effector proteins by the TTSS. The molecular structure of such a secretion signal is, however, so far unknown. The binding of specific chaperons has been shown to be necessary in some cases [22] but does not seem to be a general prerequisite. Several studies indicate a signal in the N-terminus either encoded in the underlying mRNA [23],[24] or in the peptide [12],[25],[26]. Subtil et al., for example, successfully screened for TTSS effectors using fusion proteins consisting of a chlamydial N-terminus and a reporter gene in a heterologous *Shigella flexneri* assay [12]. This experiment showed that the first 15 amino acids are sufficient for the secretion of several chlamydial effectors.

In this work we demonstrate that information derived from N-terminal peptides is universally applicable to successfully predict type III secreted proteins. We have implemented EffectiveT3, the first general prediction software for type III effector proteins. This software is based on a machine learning approach and can be applied to single proteins as well as complete proteomes. We investigate the molecular shape (i.e., length, position, composition) of the signal captured by the EffectiveT3 software and demonstrate that the signal is taxonomically universal. We applied the EffectiveT3 software to 739 prokaryotic proteomes and discuss the sizes of predicted secretomes.

## Results/Discussion

### Common features of known effector proteins

To comprehensively investigate the nature of the TTSS signal, we compiled a database of known effector proteins from members of the phylum *Chlamydiae* and the genera *Escherichia*, *Yersinia* and *Pseudomonas* by an exhaustive mining of literature. These “animal pathogen” and “plant symbiont” sets consist exclusively of proteins with individual experimental evidence for type III mediated transport and comprise 100 proteins including 48 effectors from animal pathogens/symbionts and 52 effectors from plant symbionts (Table S1). 39 of them can be clustered by sequence similarity into 15 distinct orthologous groups (see Table S2). These orthologous groups, however, turned out to be restricted to their respective taxon. Their members have no counterparts with significant homology over the major part of their sequences in other organisms included in this study.

To investigate whether predicted functional interactions based on genomic context methods [27] could be used for the prediction of TTSS effectors, we analyzed all known effectors using the STRING database [28]. A few cases of conserved chromosomal neighbourhood of effectors with structural TTSS proteins or chaperones could be observed, whereas most effectors do not co-evolve with the TTSS (Table S3). The genomic neighbourhood of known effectors has been further examined by statistical analysis of all co-localized proteins. Components of the TTSS are significantly enriched in the proximity of effectors (Table S4). The highest significance of this enrichment has been observed within the range of 30 proteins up- and downstream. Within these neighbours, 7 structural TTSS proteins show individual enrichment of statistical significance (Table S5). However, particularly in genomes encoding the TTSS on the chromosome as e.g. *Chlamydiae*, the majority of effectors cannot be found in genomic proximity to components of the TTSS (Table S6). Thus we cannot derive a general co-evolution rule for all effectors, which limits the predictive power of genomic context methods significantly. However, the observed co-evolution of certain effectors with each other and the co-localization of several effectors with TTSS components and chaperones make this methodology valuable for situations if such effectors or chaperones are already known or if the TTSS is encoded on a plasmid or on a genomic island.

In a next step we analyzed the N-terminal amino acids of known TTSS effectors in greater detail. Within their N-terminal peptides, the effectors did not show any conserved residues in several multiple sequence alignments performed and analyzed (see Figure S1 for an example). The absence of conserved positions indicative of a common sequence motif or domain signature, which could serve as a signal, demonstrated that a conserved binding domain can be excluded as a general TTSS signal.

A secretion signal could also be encoded in the secondary structure of the N-terminus. We employed secondary structure predictions and counted the structural features (coil, α-helix, β-sheet) at each residue within the first 25 amino acids. In the known TTSS effectors, 51% coil, 39% α-helix and 10% β-sheet have been predicted. In randomly selected proteins (not known to be secreted via a TTSS) we predicted 39% coil, 45% α-helix and 16% β-sheet, which indicates that coiled regions are enriched in the N-termini of TTSS effectors.

**Figure 1:**
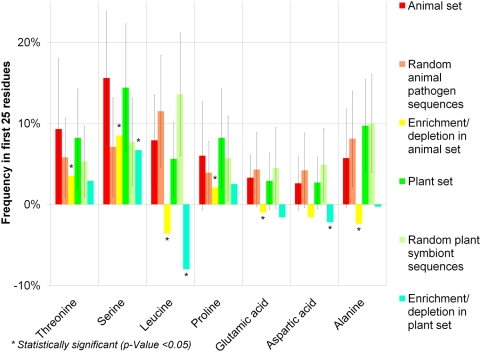
Enrichment of amino acids in effector N-termini. Amino acids that are significantly enriched or depleted in the first 25 residues of effectors from the animal pathogen effector set and from the plant symbiont effector set (p-Value<0.05 in the one sided Mann-Whitney test in at least one of the sets). Frequencies are given as percentage of amino acids within the 25 first residues. Error bars represent one standard deviation in plus and one standard deviation in minus directions.

These findings fit well with data from *P. syringae*, a well-studied plant pathogen, for which an unusual amino acid composition in the N-termini of effectors has been reported [16]–[18],[29]. Therefore we tested, whether this unusual amino acid composition is a general feature of effector proteins. A Mann-Whitney test on amino acid frequencies derived from both the whole sequences and the first 25 residues of the N-termini from the effector sets and randomly selected proteins revealed significant enrichments and depletions of certain amino acids in sequences from animal pathogens and plant symbionts, respectively (Figure 1). This effect is particularly strong in the N-terminal end and therefore, this composition bias could reflect an exploitable signal of TTSS mediated transport. The most significant enrichment in the N-termini of effectors of animal pathogens and plant symbionts is that of serine. Threonine and proline are significantly enriched in the effectors of animal pathogens, and leucine is depleted in both animal and plant effector proteins. Notably, the enrichment of proline could explain the enrichment of coiled regions in the N-termini as this amino acid is known to be less frequent in α-helices and β-sheets. Interestingly, these experiments revealed both commonalities and differences between the N-terminus of effector proteins from plant and animal pathogens, respectively.

### Modeling of the N-terminal TTSS signal peptide using a machine learning approach

The evidence for an unusual amino acid composition in the N-terminus of known TTSS effectors and the lack of a common sequence motif or domain signature prompted us to use a machine learning approach based on a binary classifier to model the TTSS secretion signal. Binary classification algorithms, such as the naive Bayes algorithm [30], are trained by a positive and negative set of instances, each instance represented by a vector of features. The algorithms weight each feature (or combinations of them) during the training process in order to achieve optimal separation between the positive and negative sets. If the performance of the classifier is high, these weights should represent the underlying biological signal. Based on our analyses of the TTSS effector sets, we represented each sequence by a collection of features comprising frequencies of amino acids, amino acid properties and short combinations of them (see material and methods). In an alternative attempt, we used features derived from the predicted secondary structure elements. Subsequently, the performance of the different classification algorithms and strategies was assessed by 10-fold cross-validation (see material and methods). The “Area Under the Curve” (AUC) value of the Receiver Operating Statistic Curve (ROC) represents the performance of a classifier describing the trade-off between sensitivity and selectivity by varying over the classifier's parameter space. The AUC summarizes this overall performance: an ideal classifier yields an AUC of 1.0, whereas a completely random prediction results in a value of 0.5. Values above 0.5 indicate a prediction above random.

A systematic comparison of different classification algorithms on the TTSS effector sets from animal pathogens and plant symbionts, respectively, resulted in a performance far above random for all classifiers tested, with an maximal AUC of 0.85 for the animal pathogen set and an AUC of 0.86 for the plant symbiont set, achieved by the complement naïve Bayesian algorithm. Both sets combined together achieved their best AUC (0.86) with the Naïve Bayesian classifier (Table 1). Training the classifier solely on the predicted secondary structure alphabet of the combined set performed well with an AUC value of 0.8. However, adding this alphabet to the sequence derived features did neither improve nor reduce the performance significantly: the test revealed an AUC of 0.87 with and 0.86 without the secondary structure features. A selection of the most discriminating features (see material and methods) resulted in a reduced list of features. These comprise not only the serine, proline and threonine frequencies as already indicated by the amino acid composition analysis, but also depletion of acidic and single alkaline residues and patterns such as the enrichment of two consecutive alkaline residues or the pattern “polar-hydrophobic-polar” (Table 2).

**Table 1:** Performance of different classification algorithms for the prediction of TTSS effectors.

Algorithm	Sensitivity	sd	Selectivity	sd	AUC	sd
**Animal pathogen set**						
Naïve Bayes complement [57]	0.77	0.02	0.79	0.04	0.78	0.02
1 nearest neighbour [58]	0.54	0.09	0.81	0.04	0.68	0.07
Logistic regression [59]	0.57	0.07	0.75	0.07	0.72	0.08
Naïve Bayes [30]	0.71	0.03	0.85	0.04	0.85	0.03
Naïve Bayes multinomial [60]	0.76	0.03	0.81	0.04	0.85	0.02
Support vector machine [61]	0.57	0.05	0.86	0.04	0.71	0.04
Voted perceptron [62]	0.24	0.04	0.97	0.02	0.78	0.01
**Plant symbiont set**						
Naïve Bayes complement	0.79	0.03	0.77	0.03	0.78	0.03
1 nearest neighbour	0.60	0.04	0.80	0.04	0.69	0.04
Logistic regression	0.62	0.03	0.74	0.06	0.73	0.03
Naïve Bayes	0.81	0.02	0.77	0.03	0.84	0.01
Naïve Bayes multinomial	0.78	0.03	0.78	0.03	0.85	0.02
Support vector machine	0.66	0.04	0.83	0.04	0.74	0.03
Voted perceptron	0.28	0.10	0.96	0.03	0.79	0.04

The performance of different classification algorithms in a tenfold cross-validation on the animal pathogen and plant symbiont training set is shown. The cross-validation has been repeated five times with different negative sets that were randomly chosen from the respective organisms. Sensitivity (defined as TP/[TP+FP]), selectivity (defined as TN/[TN+FP]), and the AUC value are given with their standard-deviation (sd) computed from the five runs.

**Table 2:** Most discriminating features between positive and negative instances.

Pattern	Enriched/Depleted
Polar–hydrophobic–polar	Enriched
Alkaline–alkaline	Depleted
Threonine	Enriched
Serine	Enriched
Proline	Enriched
Polar	Enriched
Alkaline	Depleted
Acidic	Depleted
Hydrophobic–alkaline	Depleted
Polar–polar	Enriched

The most discriminating features as reported by the feature selection procedure. Enrichment or depletion is indicated in respect to the effector class.

**Figure 2:**
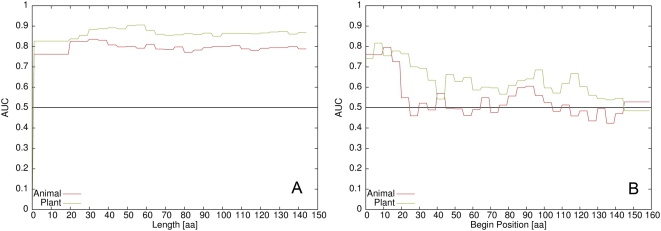
Exploration of position and length of the signal. Exploration of optimal length of the signal (A) and begin position of a 15 amino acid long window (B). The AUC value for each length and begin position is plotted for the animal pathogen set (red) and the plant symbiont set (green).

To define the part of the proteins which contributes most to the signal, we performed two experiments: First, we varied the length of the N-terminal peptide included in the analysis in order to detect the signal's length and secondly, we scanned different start positions of 15 residue long windows. For each selection of length and position, the complete feature creation, training and testing procedure was repeated. The results for these two experiments are shown in Figure 2. As high AUC values are reported over a wide range of N-terminal peptide lengths, with only a slight maximum peak at length 30 in the animal pathogen and length 50 in the plant symbiont set, the actual length of the signal is difficult to determine. However, the position scan revealed that the most discriminating positions are indeed at the N-terminus followed by a region with less predictive power. The best performance was achieved with the residues 0–30 in the plant symbiont and 0–50 in the animal pathogen set of effector proteins. Notably, also the selection 0–15 in both sets gives a good discriminative power.

Some other positions (e.g., residues 90–105 and 120–135 in the plant symbiont set) also show (an indeed weaker) predictive power which could hint to an additional signal or at least regularity in these regions. The majority of positions, however, have no predictive power due to AUC values between 0.4–0.6, and using the 15 C-terminal residues also resulted in an AUC value comparable to a random prediction (Table S7).

Taken together, these findings show the existence of a common signal encoded in the N-termini of TTSS effector proteins and are in agreement with the N-terminal signal peptide theory [23],[24]. Although it cannot be described by a pattern of conserved amino acid residues, the signal comprises a characteristic amino acid composition bias, and can thus be computationally captured using a machine learning approach. Predicted secondary structure elements show predictive power, but are substitutable by the sequence derived features. Therefore, secondary structure features are likely to be part of the signal, but are equally reflected in the sequence composition.

### The TTSS signal peptide is taxonomically universal

**Figure 3:**
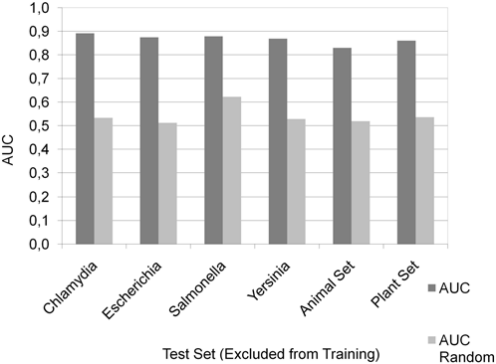
Taxonomic universality of the signal. The y-axis denotes the achieved AUC value of EffectiveT3 when trained without the positive and negative samples from the taxonomic group denoted at the bottom of the x-axis and tested against this set. The performance on a randomly chosen set of positives and negatives having the same taxonomic composition is given for comparison.

The successful applications of heterologous TTSS systems for in vitro screens [11]–[14] indicate that the TTSS secretion signal is universally understood among phylogenetically different microorganisms. The enrichment and depletion of specific amino acids in the N-termini of effectors supports this hypothesis, since the same amino acids are either depleted or enriched in the animal pathogen and plant symbiont sets (Figure 1) except for minor differences between them. To further analyze the evolutionary conservation of the type III secretion signal, we conducted the following experiment: We tested the performance to detect effector proteins in genomes which were not part of the training set and thus did not contribute in the feature selection procedure. For this, we systematically excluded genomes from training and tested the classifiers' performance not by cross-validation, but on the excluded sequences. High AUC-values between 0.83 and 0.89 were observed for all tested combinations (Figure 3, individual results of all effectors in Table S8). Notably, it was possible to predict effectors from the animal pathogen set when trained by the plant symbiont set and vice versa, yielding an AUC of 0.86 and 0.83 respectively. Therefore, the captured signal is not organism specific but must be taxonomically universal.

### Evolutionary history of the TTSS signal peptide

Since the N-terminal TTSS signal is universally detectable, we tested, whether its acquisition during evolution also follows a regular pattern. We investigated this by comparing validated effector proteins with their orthologous counterparts in organisms without TTSS. If a regular acquisition of the signal peptide by N-terminal fusion events occurs, this should be reflected in a regular, N-terminal extension of effector proteins compared with their non-effector orthologs. To test this, we performed two experiments: a systematic multiple sequence alignment approach of effectors and orthologs which are sure non-effector sequences and a pair wise sequence alignment analysis, in which individual elongations and truncations between effectors and non-effector orthologs were assessed.

In total, we could build alignments for 10 orthologous groups containing effector proteins and sure non-effector proteins. A manual inspection of the multiple alignments did not reveal a clear pattern which would support regular fusion events. This result is further supported by the pair wise analysis: Elongations of the effector sequences compared to non-effectors are less frequent (30%) than truncations (57%), whereas a similar length of effector and non-effector occurs in 13% of all pairs (Table S9, Figure S2). All three events can be detected within the same orthologous group. HopAK1, a *Pseudomonas syringae* effector, is the only example which is more often elongated (three cases) than truncated (one case). A similar picture can be seen when only the length of the N-terminal regions before the first common functional domain of effector and non-effector orthologs were compared: N-terminal regions with equal lengths can be found in 4%, shorter lengths for the effector in 39% and longer lengths for the effector in 57% of cases (data not shown).

For elucidating the evolutionary acquisition of the TTSS signal peptide we therefore suggest a model of convergent sequence adaptation. Under the selective pressure of a type III secretion system, the N-terminal sequences of all proteins which are exposed to translocation (e.g., by their cellular localization and transcriptional regulation) have adapted towards or against translocation and thus became effectors or non-effectors. Such a convergent evolutionary acquisition is in congruence with the absence of sequence homology between most of the known type-III effectors. In addition to this general mechanism, singular terminal re-assortment events as described by Starvinides and coworkers [31] might accelerate the acquisition of TTSS signal peptides.

### The signal is robust against point mutations and can even tolerate frame shifts

Our *in silico* model of the N-terminal secretion signal allows the simulation of its robustness against point mutations. In a first experiment, we exchanged residues accumulatively by random. The signal turned out to be robust when changing arbitrary residues: after one point mutation 97% after five 75% and after ten 54% of the effector proteins still have a detectable signal (Figure S3). In a second experiment, we favoured to exchange these features, which we found to have the strongest influence on the signal. For example, we depleted the amount of serine and threonine and exchanged them in favour of arbitrary residues. In this procedure, the signal rapidly breaks down: after one mutation 93% of the effectors, but only 27% after five and 2% after ten mutations carry a detectable signal (Figure S3). Therefore, the signal is robust against single and multiple point mutations as long as the significant enrichments and depletions of certain amino acids are not altered.

Schneewind and coworkers [32] showed that frame shift mutations in the mRNA altering the N-terminal peptide sequence did not abolish transport of three TTSS effector proteins of *Yersinia* species. This seems to contradict the N-terminal signal peptide hypothesis but could be explained, if the frame shifts lead to altered amino acids in the N-terminus, which nevertheless retained the characteristic features of the TTSS signal. Nine example frame shifts are given in this study which did not abolish secretion. One *Yersinia* protein (YopQ) could not be predicted as effector by our method and thus represents a false negative prediction. From the remaining six frame shifts in two proteins (YopE and YopN), only the −2 frame shift of the YopN N-terminus did not lead to a loss of the TTSS signal. The same behaviour has been shown for the Salmonella effector InvJ which tolerates +1 and −1 frame shifts [33]. In the case of the +1 frame shift the signal is still revealed by EffectiveT3, whereas no signal can be detected for the −1 frame shift. In order to assess the sensitivity of the TTSS signal towards frame shift mutations in a more systematic manner, we artificially introduced all possible frame shift mutations into the 74 known and positively predicted effectors. As control, we applied the same procedure to a set of 199 randomly selected and negatively predicted control sequences. In 15 cases (10%) of the effector mutants, the signal was preserved (Table S10), in contrast to 31% of the control sequences (data not shown). This unexpectedly high rate of preservation in non-effector mutants results from specific amino acid enrichments and depletions in the mutated sequences, which are very similar to the characteristics of TTSS effectors (data not shown). Surprisingly and in agreement with the mRNA signal hypothesis [23],[24], three effector sequences are resistant to both kinds of shifts, the +1 and +2 mutations (Table S10). Taken together, our data suggests that while some TTSS effectors surprisingly tolerate frame shifts without losing the amino acid secretion signal, most of the known effectors are sensitive towards frame shift mutations.

### A substantial fraction of proteomes is predicted as secreted

To predict type III secreted proteins for whole genomes, we applied our software EffectiveT3 on 739 bacterial and archaeal proteomes. We chose all completely sequenced prokaryotes for which the presence or absence of a type III secretion system could be determined using the KEGG database [34] and for which the cell wall type (Gram-negative vs. Gram-positive) has been unambiguously described (Table S11). In organisms encoding a TTSS, a substantial fraction of proteins is predicted as secreted, varying between 2% and 7% percent with an average of 4% of all proteins. In organisms without a TTSS, the fraction of positive predictions varies widely between different taxonomic groups. *Gammaproteobacteria* without a TTSS mostly contain a less or similar percentage of positives as *Gammaproteobacteria* with a TTSS. Interestingly also *Deinococci* (6%) and the Gram-positive *Actinobacteria* (up to 10%) exhibit high percentages of positives despite the differences in cell wall composition and the absence of a TTSS. Contrarily, *Archaea* and *Firmicutes* exhibit a very low amount of positives with 1%, respectively 2% on average. Between more closely related bacteria, similar percentages of predicted TTSS effectors were found in different strains of e.g. *S. enterica* (on average 3%) and *E. coli* (3%). The amoebae symbiont *Protochlamydia amoebophila* exhibits a slightly higher percentage (6.1%) compared to its chlamydial relatives, which are pathogens of animals and humans (on average 5%).

**Figure 4:**
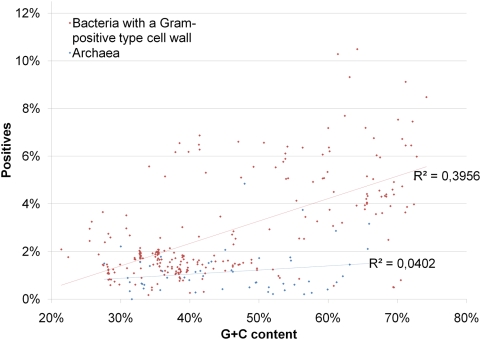
Overview of EffectiveT3 predictions in complete genomes from Gram-positive bacteria and archaea. The figure shows the percentage of positive predictions in proteomes from Gram-positive bacteria and archaea, respectively, depending on the G+C content of the genomes. Linear fits are shown by trend lines in the colours of the respective data sets; attached are the coefficients of determination R^2^ of each fit. The individual results for all proteomes can be found in Table S11.

The surprisingly high number of (false) positives in genomes without TTSS exceeds the expected false positive rate (Table 1) and thus raised questions about their nature. Manual inspection of positive predictions in Gram-positive bacteria revealed many cases of wrongly annotated gene starts (having N-terminal elongations and thus contain fractions of the intergenic space) or questionable genes without any homologs in other genomes (ORFans). Although genome annotation errors have many different reasons, they are more likely in G+C rich genomes due to the long average lengths of open reading frames [35]. When comparing the number of positives with the genomic G+C content, a partially linear relationship can be seen for Gram-positive bacteria (Figure 4; R^2^∼0.4). In agreement with the mutation experiments (see above), which showed similar characteristics of the N-termini from effectors and many nonsense peptides after frame shift mutations, unexpectedly high fractions of positives in Gram-positives are likely to be artefacts from misannotations. To distinguish between wrongly annotated gene starts and ORFans, we assessed the specificity of positive predictions for N-terminal sequences by calculating a genome wide Z-Score (see material and methods). Proteomes with a high Z-Score (>1) are enriched in effector-like sequences in the N-termini. Low Z-Scores indicate the presence of ORFans, which show similar characteristics to type III effectors over their whole length (Table S11).

**Figure 5:**
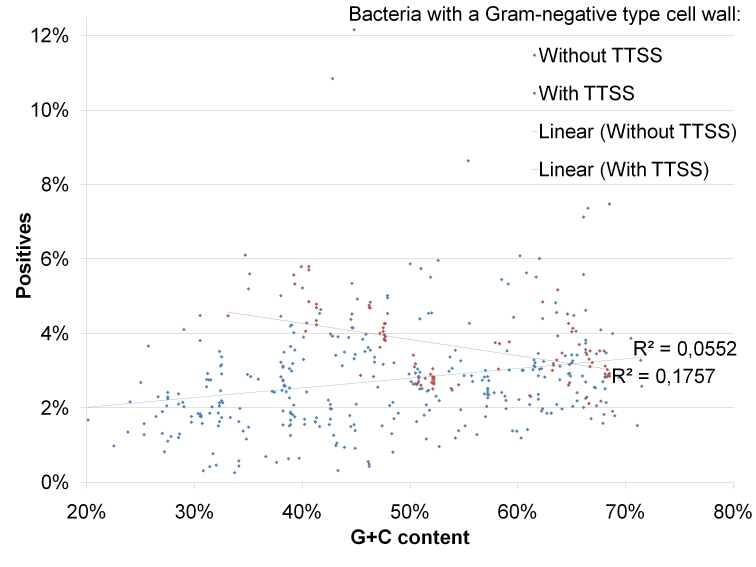
Overview of EffectiveT3 predictions in complete genomes from Gram-negative bacteria with and without TTSS. The figure shows the percentage of positive predictions in proteomes from Gram-negative bacteria with and without TTSS, depending on the G+C content of the genomes. The plot has been scaled as Figure 4 to facilitate comparison. Linear fits are shown by trend lines in the colours of the respective data sets; attached are the coefficients of determination R^2^ of each fit. The individual results for all proteomes can be found in Table S11.

In Gram-negative bacteria, the correlation between the number of positives and the genomic G+C content is much weaker (R^2^∼0.06) than in Gram-positives (Figures 4 and 5). Additive to the expected false positive rate, most proteomes with TTSS encode more putative effectors than their relatives without TTSS. The missing clear difference between Gram-negatives with and without TTSS may be explained by the noise caused by misannotations which seem to be present in all selected genomes (data not shown). Additionally, putative Type III effectors may not be a unique feature of species encoding a TTSS but could be ubiquitous in a broad range of phylogenetically diverse microbes. This finding would be surprising, but could be explained by the absence of evolutionary pressure on N-termini towards not to be secreted in microorganisms without a TTSS. Additionally, effector proteins might be subject of horizontal gene transfers into genomes without TTSS where they neo-functionalize but keep their N-termini.

### Conclusion

The TTSS is a key virulence factor in many important human pathogens, such as *Salmonella* sp., *Yersinia* sp., *Chlamydiae* and *E. coli*. However, the prediction of TTSS effector proteins was possible so far only on a small taxonomic scale, impeding the study of this important group of virulence factors in newly sequenced genomes of organisms without well-studied close relatives. In this study we describe the identification of taxonomically universal features of TTSS effector proteins, which formed the basis of the development of the program EffectiveT3, the first universally applicable *in silico* prediction method for TTSS transported proteins.

The core of our *in silico* prediction method consists of a machine learning approach, which behaves like a black-box in the sense that it does not imitate the unknown biological mechanism itself but models regularities in the N-terminal peptides of TTSS effectors. Since the training set comprised no other common feature beside TTSS mediated transport, EffectiveT3 must capture the sequence related parts of the biological signal. In contrast, it has not been possible to learn on equally sized, randomly selected sequences using the same machine learning protocol. Thus the predictive performance cannot result from a selection bias introduced by small training sets.

EffectiveT3 performs far above random in the cross-validation as well as on data derived from organisms which were not present in the training set. A certain degree of generality of the TTSS substrate recognition process was already suggested by heterologous secretion assays [12]. Our computational model demonstrates that the signal is indeed highly conserved over a broad taxonomic range, facilitating the prediction of plant symbiont effectors using information derived from animal pathogens (and vice versa). This taxonomic universality of the TTSS secretion signal implies a common mechanism of TTSS substrate recognition across phylogenetically diverse bacterial groups.

The great value of the EffectiveT3 method is its independency from sequence similarity to known effectors and the independence of organism specific *a priori* knowledge. It is therefore suited to the application on newly sequenced genomes from bacteria with a Gram-negative type cell wall and for the detection of novel effector families, which could lead to the discovery of so far unrecognized virulence factors and thus improve our understanding of the ways of host cell manipulation by bacterial pathogens. Since the procedure reveals a substantial fraction of false positive predictions and is intrinsically sensitive to misannotations such as wrongly annotated gene starts and ORFans, the current method should be complemented by specific pre- and postprecessing steps:

1. Before applying EffectiveT3, the gene annotations of the analyzed proteins should be verified to remove ORFans and ensure correct translational start sites.
2. An additional protocol to filter and rank the positive predictions by reliability might include the exclusion of already annotated genes, house-keeping genes and proteins with a signal for other transport routes as the SecA pathway.
3. Particularly in genomes which encode TTSS components on plasmids or genomic islands, the genomic proximity of TTSS components might be enriched in effectors and should be analyzed additionally.

The most promising improvement of our computational model would be the consideration of the transcriptional control of effector proteins [36]. It can be expected that genome-wide transcriptional data will become available in the near future for a sufficient number of genomes having known type III effectors.

The EffectiveT3 predictions can be accessed online at http://www.chlamydiaedb.org. The software is freely available from the authors upon request.

## Materials and Methods

### Data sets

The known type-III effector proteins have been collected manually from the literature. Each protein has been included if it has at least one direct evidence for TTSS mediated transport resulting from a single experiment. Not included are proteins, which are part of the TTSS needle complex although some of them are transported by the TTSS and data from large scale screens. By this procedure, we collected a animal pathogen set of 48 proteins comprising the taxa *Chlamydia* (17 sequences), *Salmonella* (9 sequences), *Yersinia* (15 sequences), *Escherichia* (7 sequences). A representation of this set with only one member of each orthologous group has been created separately. The sequences were downloaded from SWISSPROT/UNIPROT [37] (version as downloaded on 07/30/2008) or, if not contained there, downloaded from RefSeq [38] (version as downloaded on 07/30/2008). We retrieved the plant symbiont set consisting of 52 known *Pseudomonas* effector proteins from the *Pseudomonas syringae* Genome Resources database [39] (Hop virulence protein/gene database, downloaded on 07/30/2008). A complete list of used effector sequences is given in the Table S1. All effectors have been examined for correctness of translational start sites by manual inspection of multiple sequence alignments with their homologs.

Negative training sets of non-effectors have been created by randomly choosing proteins from the organisms represented in the animal pathogen and plant symbiont sets devoid of the known effectors. Each negative set is twice as large as its corresponding positive set. This procedure has been repeated five times in order to enable investigations on the influence of the negative set on the prediction.

Protein sequences from completely sequenced genomes of *Yersinia*, *Escherichia*, *Salmonella*, *Pseudomonas*, *Chlamydia* species as well as of gram(+) Bacteria, Archaea and *Gammaproteobacteria* were downloaded from RefSeq (version as downloaded on 07/30/2008) [40]. The data sets were classified into organism with and without TTSS by manual search in the literature for the case of gram(−) bacteria or generally classified as “without TTSS” in the case of gram(+) bacteria and archaea. A complete list of organisms used is given in the Table S11. A list of proteins building the TTSS system has been obtained by full-text searches against the SIMAP [41] databases using the gene-names of the TTSS compounds as given by KEGG [34].

### Grouping of training sets by homology

An all-against-all comparison of the full length-sequences using the Smith-Waterman algorithm [42] as implemented in the Jaligner package was performed [43]. For each pair, a similarity score S_ratio_ by dividing the alignment score by the selfscore is computed and sequences are iteratively grouped if they show a S_ratio_ value greater or equal 0.15. This measure is similar to the measure used by Lerat et al. in a study of genome repertoires in bacteria [44] and has been adjusted to maximal sensitivity in the detection of putative orthologs.

### Secondary structure prediction

To predict secondary structure features we used the PSIpred-software [45]. The prediction has been applied to the whole sequences. PSIpred can be applied using alignments to conserved sequences as extrinsic information using PSI-BLAST [46]. For this purpose, we performed PSI-BLAST searches against SWISSPROT/UNIPROT. For the N-terminal ends of the effectors, we did not receive a sufficient amount of alignments to improve the secondary structure prediction at these positions. As a consequence, we only used the *ab initio* prediction without alignment information. We then counted the fraction for each predicted class in the N-termini as input feature for the prediction pipeline.

### Multiple alignments of N-termini

Multiple alignments have been created using two different methods: ClustalW (Version 2.0.5) [47], and Muscle (Version 3.7) [48] with standard parameters. We randomly chose ten sequences from the sets of known effectors to create multiple alignments and aligned their 10, 20 and 30 first residues. This procedure has been repeated 20 times. We manually checked the alignments for conserved regions similar to a multiple alignment containing a certain domain signature. Example alignments are given in the Figure S1.

### Statistical enrichment analyses

Enrichments and depletions of amino acid properties (frequency, frequency of its representations in a reduced alphabet, frequency of secondary structure properties) have been performed using a one sided Mann Whitney test with p< = 0.5. We used the implementation in the Prompt software (Protein Mapping and Comparison Tool [49], which employs the statistic software R [50].

### Co-evolution of known type-III effectors and TTSS-related sequences

Predicted functional interactions between orthologous groups containing effector sequences and selected TTSS sequences (representing proteins of all orthologous groups taken from Table S1) were obtained from the STRING database [28] (Version 7.1 as downloaded on 10/03/2007). Links from genomic context methods (conserved neighbourhood, gene fusion, phylogenetic profiles) were used, the others were discarded. Links with a confidence score less than 0.5 have been discarded and the connected proteins were grouped.

For the in-depth analysis of conserved genomic proximity, complete genome and proteome data for the known effectors has been downloaded from the KEGG database [34] (release 2009/01/19). Components of the TTSS have been identified by their association to the KEGG Orthologous Groups (KO) belonging to the TTSS reference pathway KO03070 (K03219..K03230). Genomic neighbours of a certain distance to known effectors have been extracted from the KEGG data and grouped by their associated KO.

### Analysis strategy for signal acquisition

To detect regular acquisition by fusion of a signal peptide, we employed an automated alignment pipeline. Orthologous groups have been obtained from the eggNOG database [51] for each effector protein. Proteins from organisms other than *Gammaproteobacteria* have been filtered out. The remaining proteins where labelled as “effector” if in training set, “putative effector” if from an organism with TTSS or “non-effector” if from an organism without TTSS.

We cut every sequence at the start of its first functional domain as detected by Pfam [52] (as contained in InterPro Release 17.0 [53]) and created multiple alignments of the remaining N-terminal fragments. We then checked the alignments for regular N-terminal extensions of effector or putative effector proteins compared with non-effectors by manual inspection in the case of the multiple alignments. We also pair wise aligned effector/non-effector sequences from the same orthologous group and counted elongations (alignment start of the effector greater than of the non-effector) and truncations within one group. If the difference between the alignment starts was smaller than 15 residues, we counted the alignment as having the same length. The same procedure has been repeated without aligning the sequences by just comparing the lengths before the start of the functional domain.

Multiple alignments were built using ClustalW (Version 2.0.5) [47], Muscle (Version 3.7) [48], with standard parameters, pair wise alignments were calculated with the Smith Waterman algorithm as implemented in the Jaligner package using the BLOSUM62 substitution matrix.

### Feature creation

We deduced the frequencies of amino acids as well as frequencies from two reduced alphabets. The reduced alphabets are created by mapping amino acids to amino acid properties and to a hydrophobic/hydrophilic alphabet. Each amino acid is only added to one of the property classes, although some would fit to several classes. In this case, the amino acid has been added to the more specific (smaller) class. The feature mapping is listed in Table 3. We also computed the frequencies of di- and tri-peptides from each of the alphabets. From these features, we discarded all these which did not occur at least two times in either the positive or the negative data set, since these features would lead to the adaptation of the classifiers to individual sequences (over-fitting). This procedure typically reveals ∼70 features, depending on the negative set employed. The frequencies of these features range typically between 2 and 5; we could therefore use them directly as input for the machine learning algorithms without the need of further discretisation. A list of all features is given in Table S12.

**Table 3:** Mapping of amino acids to property alphabets.

Property	Amino Acids
Hydrophobic; 1st alphabet	A, G, I, L, M, V
Hydrophilic; 1st alphabet	P, H, U
Aaromatic	F,W,Y
Polar	N, Q, S, T
Acidic	D, E
Alkaline	K, L, R
Ionisable	C, Y
Hydrophilic; 2nd alphabet	S, F, T, N, K, Y, E, Q, C, W, P, H, D, R, U
Hydrophobic; 2nd alphabet	V, M, L, A, I, G

The mapping of amino acids on the two reduced alphabets (amino acid property alphabet and hydrophobic/hydrophilic alphabet) maps each amino acid to exactly one letter of the respective alphabet.

### Selection of the most discriminating features

To detect the most influential features, we applied two feature selection strategies, a greedy hill-climbing search (the BestFirst algorithm) (parameters: look-up-cache size = 1, 5 iterations) in combination with Correlated Feature Selection [54] (parameters: locally predictive = true, missing values = false) as provided by WEKA (version 3.5.6) [55].

### Learning and testing procedure

We used the implementations of several classification algorithms from the WEKA machine learning package. Each classifier has been tested five times using different negative sets (see used data sets) by a 10-fold cross-validation procedure as provided by WEKA. For cross-validation, the positive and negative sequence sets have been partitioned into 10 subsamples. In each of the 10 passes, a single subsample was retained as validation data for testing the model which has been trained using the remaining 9 subsamples.

Initially, we aligned each N-terminus of the training set with each other using the Smith-Waterman algorithm with a BLOSUM62 substitution matrix. If two sequences showed S_ratio_ (see above)>0.1 over the whole sequence or more than 0.3 in the area of the signal, one of them was discarded from the training set. This has been done to avoid learning protein-families instead of the signal. Sensitivity has been computed as TP/(TP+FN), Selectivity as TN/(TN+FP), with TP = amount true positive predictions, FN = amount false negative predictions, TN = amount true negative predictions, FP = amount false positive predictions. Receiver Operating Statistics to determine the AUC value had been created using the WEKA-toolbox. Precision and Recall are computed separately for both classes, where the AUC describes the overall performance of the classifier. The classification algorithms employed are listed in Table 3.

### Exploration of optimal position and length

To determine the optimal position and length of the signal we applied a sliding window approach varying the start and length of the sequence used for the learning and testing procedure. At each position, the whole procedure of feature selection, removal of similar sequences, training and cross-validation has been repeated. For the position exploration, we used a window of the length 15 which we moved in steps of five residues. The length exploration started with a window of the first ten residues which was elongated by five residues in each round. If a sequence was too short for the range of coordinates in a certain step of this procedure, it has been discarded from the data set. Since we found that the choice of the negative set does not significantly influence the prediction, we used only one negative set in this analysis.

### Signal robustness

The robustness of the signal has been assessed by measuring the fractions of positively predicted instances from the training set after introducing a certain amount of amino acid exchanges in the first 25 residues. We only used these sequences, which are predicted as true positives by the final classification algorithm (full training set, Naïve Bayes algorithm with selective settings [probability for class “secreted” >0.95 using the Naïve Bayesian Classifier]).

We mutated the N-terminal sequences (first 25 residues) by introducing point mutations at random positions into the underlying DNA sequences (T,A,C,G exchanged with equal probability of 1/4) which did not result in stop codons but altered the amino acid sequence. After translating the mutated sequence, we measured the fraction of positively predicted effectors after one, five and ten consecutive mutations. In a second strategy we substituted randomly selected amino acids according to their importance for the TTSS signal peptide. Residues which did not belong to the group of depleted amino acids (leucine, glutamic acid, aspartic acid and alanine) were replaced by a randomly selected member of this group of depleted amino acids. Residues which did belong to the group of enriched amino acids (threonine, serine and proline) were replaced by randomly selected amino acids which did not belong to this group of enriched amino acids (the substitution probabilities for the non-enriched amino acids have been derived from their frequency within the complete proteins without the N-terminal ends).

### The effect of frame shift mutations on the signal

We have used a data set given by Ramamurthi et al. [56] of three Yersinia effector proteins with three frame shift mutants for each. We retrained our classifier using the first 15 amino acids instead of the first 25, since only the first 15 residues of the mutants are given in the paper.

Simulation of frame shifts has been done by shifting the DNA by one (+1) and two (+2) positions. In order to get a sufficient amount of sequences with sufficient length, appearing stop codons have been replaced by methionine. We used only these effectors, which show a positive prediction with restrictive parameters (probability for class “secreted” >0.95 as reported by the Naïve Bayesian Classifier). As control, we used randomly selected sequences from the same organisms which are covered by the positive set and used only these sequences, which were negatively predicted (probability not secreted >0.95 as reported by the Naïve Bayesian Classifier). Signals conserved after frame shift were detected with the same settings as in the selection procedure.

### Taxonomic universality of the signal

Notably, a conclusion about the signal's generality cannot be deduced by the fact that the classifier performs well in the cross-validation procedure, since the algorithm might detect independent features for each taxon in this procedure. In order to test the universality of the signal, we excluded each taxon (*Yersinia*, *Salmonella*, *Escherichia*, *Chlamydia*, *Pseudomonas*) from the training and feature-selection procedure and tested the classifiers performance with this taxon as separate test set. For both sets, negative sets twice as large are randomly created from these organisms, which are also in the respective positive set. The values for the AUC have been computed using the WEKA-toolbox.

### Final training of the classifier for the prediction of secretomes

The final classifier has been obtained using both sets of known effectors and a negative set which was twice as large as the positive set. We used the Naïve Bayes algorithm as it showed the best overall performance in the cross-validation procedure. Again, we excluded similar N-termini and used the first 25 amino acids as primary input. The sequence data of the proteomes has not been pre-filtered or further processed for the prediction of effectors in complete genomes. To investigate the influence of the amino acid frequencies within each proteome, the prediction of effectors has been also performed in pseudo-proteomes, for which all protein sequences have been denaturised by random shuffling. The shuffling process has altered only the order of amino acids within the proteins but not their overall (genome-wide) frequency.

### Implementation of the effectiveT3 software

The EffectiveT3 software is based on the WEKA toolbox and implemented purely in the Java™ programming language. The probability threshold for class “secreted” using the Naïve Bayesian Classifier can be selected by the user in order to adjust the selectivity and sensitivity of the predictions. We offer a web-interface for own predictions at http://www.chlamydiaedb.org.

### Application of effectiveT3 to complete archaeal and bacterial proteomes

Complete genome and proteome data of prokaryotic genomes has been downloaded from the KEGG database [34] (release 2009/01/19). Components of the TTSS have been identified by their association to the KEGG Orthologous Groups (KO) belonging to the TTSS reference pathway KO03070 (K03219..K03230). Genomes in which at least 9 of these 12 KO are present have been considered as genomes with TTSS. Genomes in which less than 6 of these 12 KO are present have been considered as genomes without TTSS. All genomes in which between 6 and 8 of these 12 KO are present have been excluded from this analysis to avoid uncertainty. Additionally, all bacterial genomes have been excluded from this analysis for which no information on cell wall type (Gram-positive vs. Gram-negative) was available at the NCBI Entrez Genome Project Organism Info database [38]. For the remaining 739 proteomes, EffectiveT3 predictions have been calculated using a selective parameter setting (probability for class “secreted” >0.99 using the Naïve Bayesian Classifier).

To estimate the enrichment of TTSS effector-like sequences in the N-termini of the proteomes, a genome-wide Z-Score is calculated for every proteome: Z = (N-A)/SD, whereas N denotes the number of positives in the N-termini of the real proteome. A and SD are derived from 50 repetitions predicting positives in randomly chosen segments of 25 aa length (one segment per protein), whereas A corresponds to the average number of positives in the 50 runs and SD to their standard deviation.

## Supporting Information

Figure S1 Example alignment of N-termini. The first 30 residues of non-homologous effector proteins have been aligned using ClustalX with default parameters.
(4.31 MB TIF)

Figure S2 Example alignments between effector and non-effector orthologs. To investigate the evolutionary acquisition of the signal peptide, a pair wise sequence alignment study counting individual elongations and truncations between effectors and non-effector orthologs has been performed. This figure shows examples of these alignments. A) demonstrates elongation and B) truncation of effector proteins (upper row) aligned with sure non-effector proteins (lower row).
(1.31 MB TIF)

Figure S3 Robustness of the TTSS secretion signal against point mutations. The diagram depicts the percentage of positively predicted TTSS signals after accumulation of point mutations. The non-targeted mutation strategy exchanged residues accumulatively by random. The targeted mutation strategy favoured to exchange these features, which we found to have the strongest influence on the signal. For both experiments all positively predicted proteins from the animal pathogen and plant symbiont training sets have been used.
(0.09 MB TIF)

Table S1 Effector and TTSS sequences used in this study. Effector proteins are listed first, then the sequences of the TTSS system and few examples of TTSS related chaperones. The different sets are denoted as follows: A = animal pathogen set, P = plant symbiont set, T = type III secretion system, C = TTSS related chaperone. For each sequence, the first 25 N-terminal amino-acids are given.
(0.20 MB DOC)

Table S2 Orthologous groups of effector proteins. This table comprises effector proteins with individual experimental evidence for type III mediated transport which can be clustered into orthologous groups (clustered by homology and manual inspection). A sequence is added to a cluster, if it has at least S_ratio_> = 0.15 to one other cluster member. S_ratio_ is computed as alignment-score/selfscore.
(0.08 MB DOC)

Table S3 Groups of co-evolving effector and TTSS proteins and examples of co-localized effector proteins and chaperones based on the STRING database. For each group of co-evolving effector and TTSS proteins, gene names of the members are given. The right column indicates, whether the orthologous group comprises effectors, TTSS proteins or TTSS related chaperones. A gene is added to a cluster, if the score of a genomic context method to another member derived from STRING exceeds 0.5. In the last section, examples of co-localized effectors and chaperones are listed.
(0.05 MB DOC)

Table S4 Number of genomic neighbours of known effectors, number of non-neighbours and their association to the TTSS. For all known effectors from Table S1, genomic neighbours have been determined for a certain distance upstream and downstream on the chromosome or plasmid. These neighbours and the remaining, non-neighboured proteins of the genomes have been distinguished by their association to the TTSS. Components of the TTSS are enriched in the neighbourhood of effectors. The statistical significance of this enrichment has been determined using the t-Test. The most significant enrichment of TTSS components in the genomic neighbourhood of effectors can be observed within the range of 30 neighbours up- and downstream (marked in red).
(0.04 MB DOC)

Table S5 Enrichment of KEGG orthologous groups within the genomic neighbourhood of known effectors. This table lists KEGG orthologous groups (KO), which are significantly enriched (Bonferroni-corrected t-Test p-Value<0.05) within 30 neighbours up- and downstream of known effectors.
(0.03 MB DOC)

Table S6 Known effectors and their genomic neighbourhood to TTSS components. The genomic neighbourhood (30 genes up- and downstream) to TTSS components has been evaluated for all known effectors, except on Yersinia pestis KIM due to the absence of the plasmid pCD1 from the KEGG database. The number of effectors which are neighboured to at least one TTSS component is given in the middle column, the remaining effectors are summarized in the right column.
(0.04 MB DOC)

Table S7 Performance of the classifiers using the C-terminal end. To prove the concept of the N-terminal signal peptide, C-termini should have no predictive power. The performance for several classifiers has been evaluated using exactly the same feature selection, training and test procedure as used for the N-termini. 5 runs with different negative sets have been performed.
(0.03 MB DOC)

Table S8 Prediction results with EffectiveT3 trained without a certain taxonomic sub-set. EffectiveT3 has been trained without the positive and negative samples from the excluded taxonomic groups listed in this table. Testing EffectiveT3 on these effectors (E) and randomly chosen negative samples (R) resulted in true positive (+E), false negative (−E), false positive (+R) and true negative (−R) predictions.
(0.35 MB DOC)

Table S9 Pair wise comparison of orthologous effector and non-effector proteins. Truncations, elongations and conservations of the N-terminal length until the first functional domain are listed according to the effector protein (first column) compared to orthologs from non-TTSS bearing organisms.
(0.05 MB DOC)

Table S10 Effector sequences which tolerate frame shift mutations. The mutations were introduced by either shifting the DNA sequences by one or two bases to the left, stop codons where replaced by Methionine.
(0.04 MB DOC)

Table S11 EffectiveT3 predictions in complete proteomes. EffectiveT3 predictions for complete proteomes have been grouped by Archaea, Gram-positive and Gram-negative bacteria. Within each group, proteomes are sorted by their taxonomic lineage and species names. For each proteome, the absence (−) or presence (+) of a TTSS, the genomic G+C content, the number of annotated proteins, the percentage of EffectiveT3 positive predictions and the genome-wide Z-Score are given. The presence of the TTSS in the proteomes as determined by KEGG and the hosts are coded by the following colors: black = without TTSS or unknown host; red = with TTSS/animal pathogenic; green = with TTSS/plant symbiotic.
(0.98 MB DOC)

Table S12 Input features of the machine learning algorithms after initial feature selection. This table comprises these features, which are selected from all possible feature combinations using three different alphabets (amino acid alphabet, amino acid property alphabet, hydrophobic/hydrophilic alphabet) with a maximal pattern length of three. In order to avoid over-fitting on the data, only features are selected which are not specific to either the positive or the negative set but exists in both.
(0.07 MB DOC)
