# Correction: Sequence-Based Prediction of Type III Secreted Proteins

**DOI:** 10.1371/annotation/78659a32-7869-4b14-91a6-b301a588d937

**Published:** 2009-04-28

**Authors:** Roland Arnold, Stefan Brandmaier, Frederick Kleine, Patrick Tischler, Eva Heinz, Sebastian Behrens, Antti Niinikoski, Hans-Werner Mewes, Matthias Horn, Thomas Rattei

The affiliation for the eighth author, Hans-Werner Mewes, is missing an institution. This author should also be affiliated with affiliation #1: Technische Universität München, Department of Genome Oriented Bioinformatics, Wissenschaftszentrum Weihenstephan, Freising, Germany.

