# Supplementary figures and images for: Sequence-Based Prediction of Type III Secreted Proteins
Source: PLoS Pathog. 2009 Apr 24;5(4):e1000376. doi: 10.1371/journal.ppat.1000376 (PMC2669295; doi:10.1371/journal.ppat.1000376)

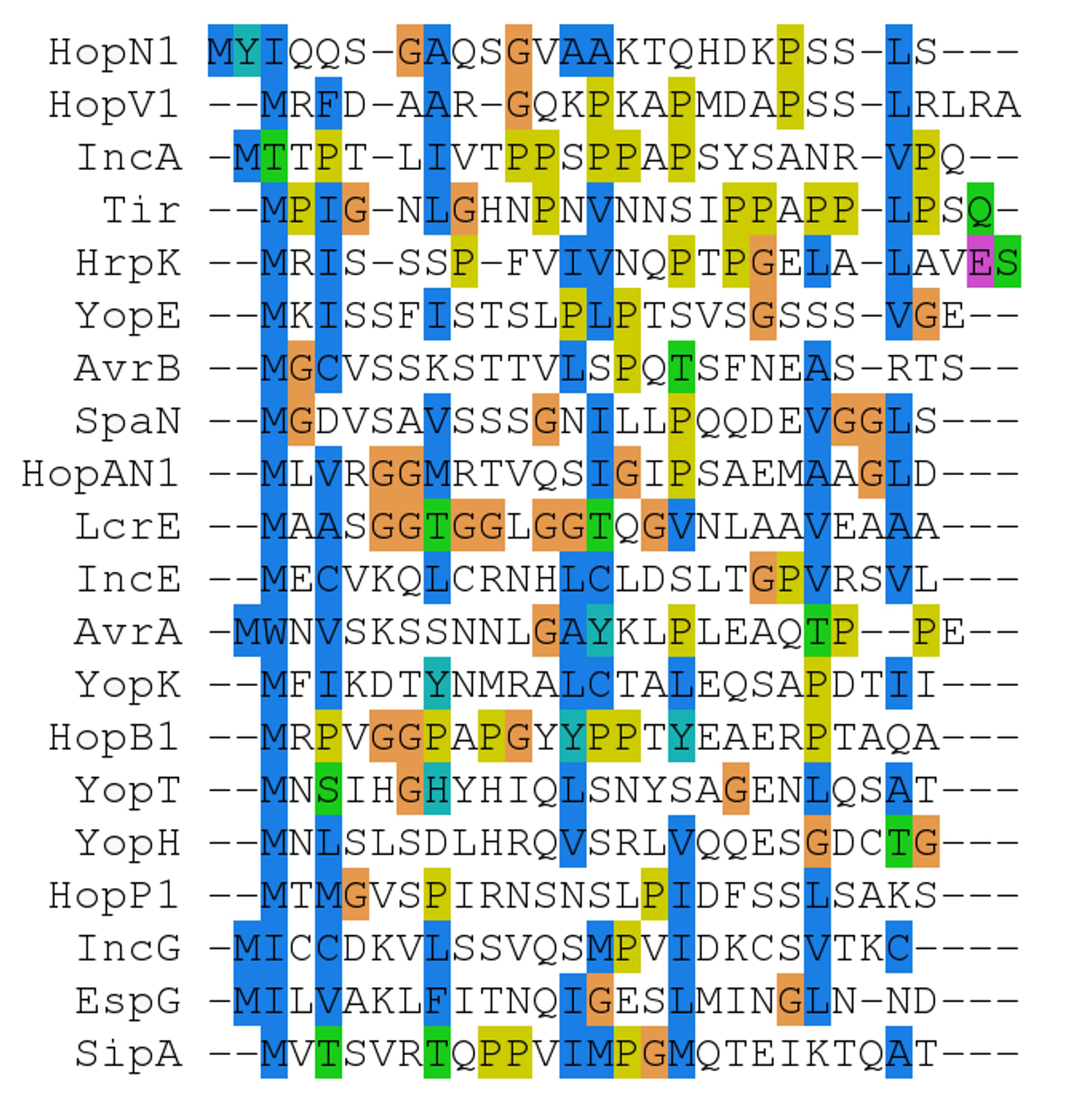

Supplement: Figure S1 — Example alignment of N-termini. The first 30 residues of non-homologous effector proteins have been aligned using ClustalX with default parameters. (4.31 MB TIF) [file ppat.1000376.s001.tif]

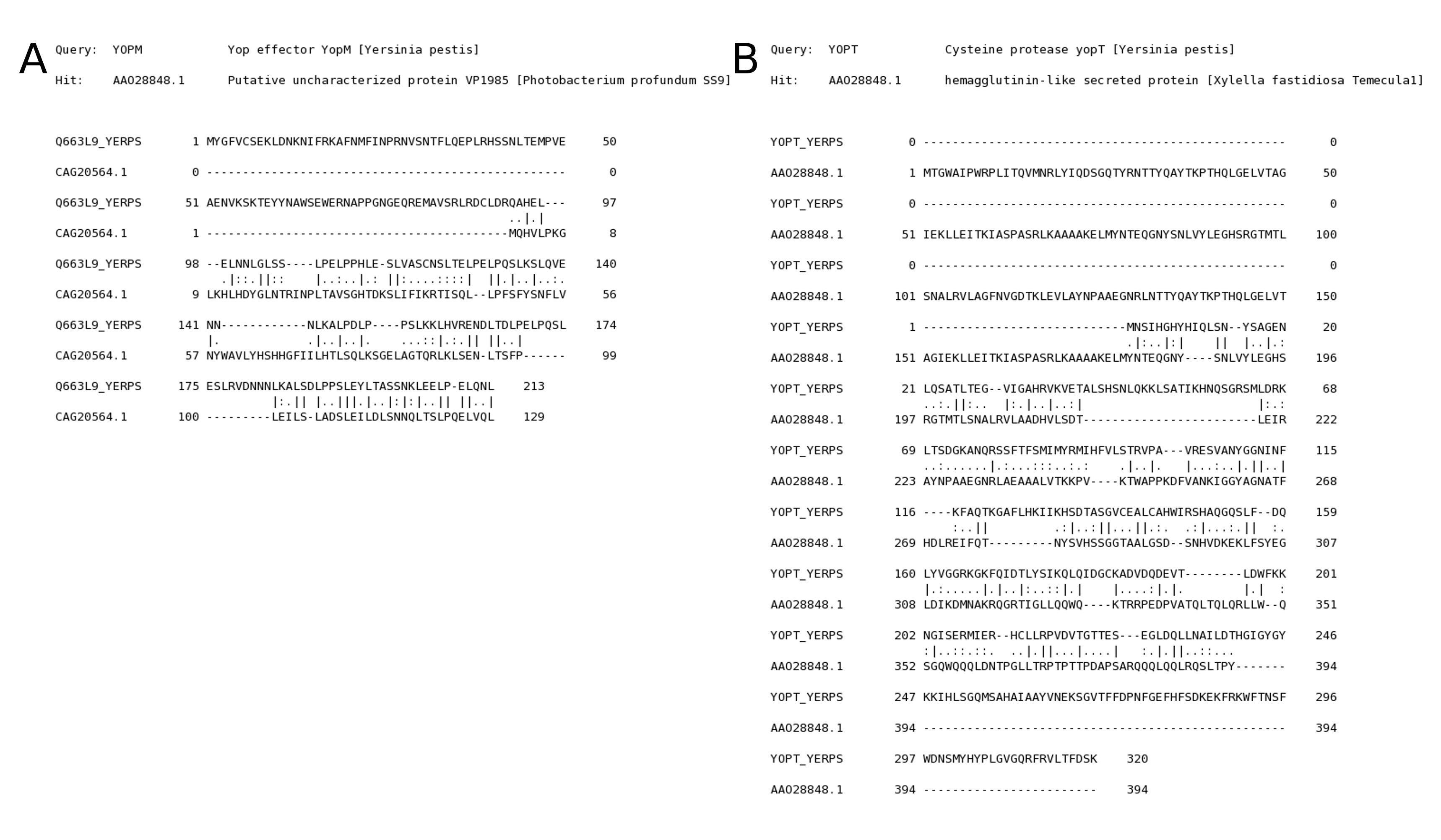

Supplement: Figure S2 — Example alignments between effector and non-effector orthologs. To investigate the evolutionary acquisition of the signal peptide, a pair wise sequence alignment study counting individual elongations and truncations between effectors and non-effector orthologs has been performed. This figure shows examples of these alignments. A) demonstrates elongation and B) truncation of effector proteins (upper row) aligned with sure non-effector proteins (lower row). (1.31 MB TIF) [file ppat.1000376.s002.tif]

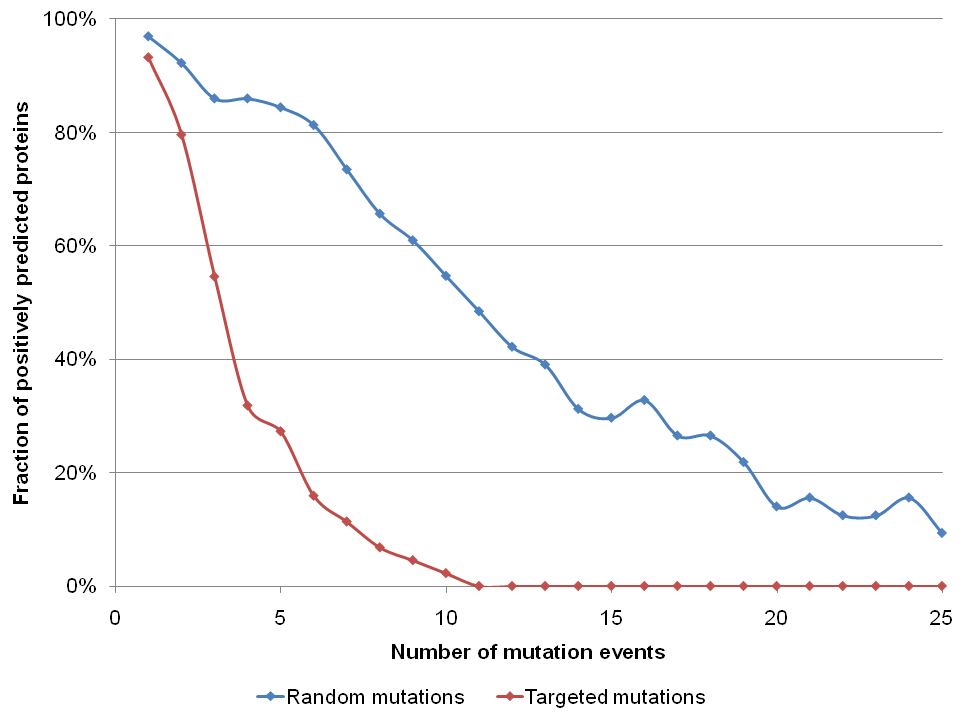

Supplement: Figure S3 — Robustness of the TTSS secretion signal against point mutations. The diagram depicts the percentage of positively predicted TTSS signals after accumulation of point mutations. The non-targeted mutation strategy exchanged residues accumulatively by random. The targeted mutation strategy favoured to exchange these features, which we found to have the strongest influence on the signal. For both experiments all positively predicted proteins from the animal pathogen and plant symbiont training sets have been used. (0.09 MB TIF) [file ppat.1000376.s003.tif]
