# Supplementary material for: Sequence-Based Prediction of Type III Secreted Proteins
Source: PLoS Pathog. 2009 Apr 24;5(4):e1000376. doi: 10.1371/journal.ppat.1000376 (PMC2669295; doi:10.1371/journal.ppat.1000376)
Supplement: Table S1 — Effector and TTSS sequences used in this study. Effector proteins are listed first, then the sequences of the TTSS system and few examples of TTSS related chaperones. The different sets are denoted as follows: A = animal pathogen set, P = plant symbiont set, T = type III secretion system, C = TTSS related chaperone. For each sequence, the first 25 N-terminal amino-acids are given. (0.20 MB DOC) [file ppat.1000376.s004.doc]

Table S1. Effector and TTSS sequences used in this study

Effector proteins are listed first, then the sequences of the TTSS system and few examples of TTSS related chaperones. The different sets are denoted as follows: A=animal pathogen set, P=plant symbiont set, T=type III secretion system, C=TTSS related chaperone. For each sequence, the first 25 N-terminal amino-acids are given.

| **Set** | **Accession** | **Organism** | **Name** | **N-terminal sequence** |
| --- | --- | --- | --- | --- |
| A | A6M3N5 | Yersinia pestis CA88-4125 | YopE | MKISSFISTSLPLPTSVSGSSSVGE |
| A | Q7BRY7 | Yersinia enterocolitica | YopE | MKISSFISTSLPLPASVSGSSSVGE |
| A | A9R9K8 | Yersinia pestis bv. Antiqua (strain Angola) | YopH | MNLSLSDLHRQVSRLVQQESGDCTG |
| A | Q7BRY8 | Yersinia enterocolitica | YopH | MNLSLSDLHRQVSRLVQQESGDCTG |
| A | B0A3S4 | Yersinia pestis biovar Orientalis str. F1991016 | YopK | MFIKDTYNMRALCTALEQSAPDTII |
| A | Q56935 | Yersinia pseudotuberculosis | YopK | MFIKDTYNMRALCTALEQSAPDTII |
| A | Q7BS06 | Yersinia enterocolitica | YopQ | MFIKDAYNMRALCTALEQSAPDTII |
| A | B0HNN9 | Yersinia pestis biovar Antiqua str. B42003004 | YopJ | MIGPISQINISGGLSEKETSSLISN |
| A | Q93KQ5 | Yersinia enterocolitica | YopP | MIGPISQINSPGGLSEKETSSLISN |
| A | A6M3U5 | Yersinia pestis CA88-4125 | YopM | MFINPRNVSNTFLQEPLRHSSNLTE |
| A | Q663L9 | Yersinia pseudotuberculosis | YopM | MYGFVCSEKLDNKNIFRKAFNMFIN |
| A | Q93KU8 | Yersinia enterocolitica | YopM | MFITPRNVSNTFLQEPLRHSSDLTE |
| A | B0A3S3 | Yersinia pestis biovar Orientalis str. F1991016 | YopT | MNSIHGHYHIQLSNYSAGENLQSAT |
| A | Q93RN4 | Yersinia pseudotuberculosis | YopT | MNSIHGHYHIQLSNYSAGENLQSAT |
| A | P27475 | Yersinia enterocolitica | YopT | MDSIHGHYHIQLSNYSAGENLQSAT |
| A | P0C2N1 | Yersinia enterocolitica | YopT1 | MDSIHGHYHIQLSNYSAGENLQSAT |
| A | A9ZFE7 | Yersinia pestis biovar Orientalis str. IP275 | YopO | MKSVKIMGTMPPSISLAKAHERISQ |
| A | Q05608 | Yersinia pseudotuberculosis | YpkA | MKSVKIMGTMPPSISLAKAHERISQ |
| A | Q56921 | Yersinia enterocolitica | YpkA | MKIMGTMSPSISLAKAHERISKHWQ |
| A | Q93KQ5 | Yersinia enterocolitica | YopP | MIGPISQINSPGGLSEKETSSLISN |
| A | O85239 | Yersinia enterocolitica | YopO | MKIMGTMPPSISLAKAHERISQHWQ |
| A | A9ZER0 | Yersinia pestis biovar Orientalis str. IP275 | YscH | MTVTLNRGSITSLMSSSQAVSTLQP |
| A | Q663I2 | Yersinia pseudotuberculosis | YscH | MTVTLNRGSITSLMSSSQAVSTLQP |
| A | Q7BRZ4 | Yersinia enterocolitica | YscH | MTVTLNRGSITSLMSSSQAVSTLQP |
| A | O34020 | Chlamydophila caviae | CopN | MAASGGAGGLGGSQAVDVAQVQAAA |
| A | Q9Z8L4 | Chlamydophila pneumoniae | LcrE | MAASGGTGGLGGTQGVNLAAVEAAA |
| A | Q824H6 | Chlamydophila caviae | TARP | MTSPINNPSTTNVTTTTTSTPVVTT |
| A | O84462 | Chlamydia trachomatis | TARP | MTNSISGYQPTVTTSTSSTTSASGA |
| A | Q9Z7Y1 | Chlamydophila pneumoniae | TARP | MAAPINQPSTTTQITQTGQTTTTTT |
| A | Q46210 | Chlamydophila caviae | IncA | MTVSTDNTSPVISRASSPTFGDHGK |
| A | Q3KMQ0 | Chlamydia trachomatis (strain A/HAR-13 / ATCC VR-571B) | IncA | MTTPTLIVTPPSPPAPSYSANRVPQ |
| A | Q9Z8Z8 | Chlamydophila pneumoniae | IncA | MSSPVNNTPSAPNIPIPAPTTPGIP |
| A | Q46210 | Chlamydophila caviae | IncA | MTVSTDNTSPVISRASSPTFGDHGK |
| A | O84235 | Chlamydia trachomatis | IncB | MVHSVYNSLAPEGFSQVSIQPSQIP |
| A | Q9Z8P7 | Chlamydophila pneumoniae | IncB | MSAPIPTPQELSDQITCLNVQYQQV |
| A | O30783 | Chlamydophila caviae | IncC | MTSVRTDLTPGDTSLQSSLLNPSDL |
| A | O84236 | Chlamydia trachomatis | IncC | MTYSISDIAHKSDISNPTSPAPSRK |
| A | Q9Z8P6 | Chlamydophila pneumoniae | IncC | MTSPIPFQSSGDASFLAEQPQQLPS |
| A | Q9RPQ1 | Chlamydia trachomatis | IncD | MTKVYANSIQQERVVDRIALLERCL |
| A | O84118 | Chlamydia trachomatis | IncE | MECVKQLCRNHLCLDSLTGPVRSVL |
| A | O84119 | Chlamydia trachomatis | IncF | MGDVMIQSVKTESGLVEGHRGICDS |
| A | Q3KMQ1 | Chlamydia trachomatis (strain A/HAR-13 / ATCC VR-571B) | IncG | MICCDKVLSSVQSMPVIDKCSVTKC |
| A | Q9Z9F5 | Chlamydophila pneumoniae |  | MSHLIPSLRNSVTSYFHKPQPIKQA |
| A | Q9Z7W9 | Chlamydophila pneumoniae |  | MATPAQKSPTFQDPSFVRELGSNHP |
| A | P40613 | Salmonella typhimurium | SpaN | MGDVSAVSSSGNILLPQQDEVGGLS |
| A | Q56027 | Salmonella typhimurium | SipA | MVTSVRTQPPVIMPGMQTEIKTQAT |
| A | Q9RPH0 | Salmonella typhimurium | SspH2 | MPFHIGSGCLPATISNRRIYRIAWS |
| A | Q56061 | Salmonella typhimurium | SifA | MPITIGNGFLKSEILTNSPRNTKEA |
| A | Q8ZNR3 | Salmonella typhimurium | SopA | MKISSGAINFSTIPNQVKKLITSIR |
| A | Q57QR2 | Salmonella choleraesuis | SopB | MQIQSFYHSASLKTQEAFKSLQKTL |
| A | P40722 | Salmonella typhimurium | SopD | MPVTLSFGNHQNYTLNESRLAHLLS |
| A | O52623 | Salmonella typhimurium | SopE | MTKITLSPQNFRIQKQETTLLKEKS |
| A | Q7CQD4 | Salmonella typhimurium | SopE2 | MTNITLSTQHYRIHRSDVEPVKEKT |
| A | P74873 | Salmonella typhimurium | SptP | MLKYEERKLNNLTLSSFSKVGVSND |
| A | Q58I88 | Escherichia coli | Tir | MPIGNLGHNPNVNNSIPPAPPLPSQ |
| A | Q47184 | Escherichia coli | EspA | MDTSTTASVASANASTSTSMAYDLG |
| A | Q8XC86 | Escherichia coli O157:H7 | EspB | MNTIDNTQVTMVNSASESTTGASSA |
| A | Q7DB81 | Escherichia coli O157:H7 | EspD | MLNVNNDTLSVTSGVNTASGTSGIT |
| A | Q7DB85 | Escherichia coli O157:H7 | EspF | MLNGISNAASTLGRQLVGIASRVSS |
| A | O85646 | Escherichia coli | EspG | MILVAKLFITNQIGESLMINGLNND |
| A | Q8X2D5 | Escherichia coli O157:H7 | TccP | MINNVSSLFPTVNRNITAVYKKSSF |
| A | A2A0X3 | Escherichia coli O157:H- | TccP2 | MINSINSFFSGLPRSISSAIRSSTF |
| A | B2NN32 | Escherichia coli O157:H7 str. EC4196 | NleB | MLSSLNVLQSSFRGKTALSNSTLLQ |
| A | Q8XBX7 | Escherichia coli | St47 | MINPVTNTQGVSPINTKYAEHVVKN |
| P | P13835 | Pseudomonas syringae pv. glycinea | AvrB | MGCVSSKSTTVLSPQTSFNEASRTS |
| P | Q887D0 | Pseudomonas syringae pv. tomato | HopM1 | MISSRIGGAGGVELSRVNQQHDTVP |
| P | Q888Y7 | Pseudomonas syringae pv. tomato | HopQ1-1 | MHRPITAGHTTSRLILDQSKQISRT |
| P | Q7BE94 | Pseudomonas syringae pv. maculicola | AvrRpm1 | MGCVSSTSRSTGYYSGYENHEEPRV |
| P | Q886L1 | Pseudomonas syringae pv. tomato | HopAF1 | MGLCISKHSGSSYSYSDSDRWQVPA |
| P | Q88BF6 | Pseudomonas syringae pv. tomato | HopY1 | MNITPLTSAAGKGSSAQGTDKISIP |
| P | Q889A9 | Pseudomonas syringae pv. tomato | HopAJ1 | MRSRVITTSLVVIMLSCASAAPACF |
| P | Q87V79 | Pseudomonas syringae pv. tomato | HopAN1 | MLVRGGMRTVQSIGIPSAEMAAGLD |
| P | Q882F0 | Pseudomonas syringae pv. tomato | HopP1 | MTMGVSPIRNSNSLPIDFSSLSAKS |
| P | Q8RP03 | Pseudomonas syringae pv. maculicola | HopPtoA1Pma | MYINRSISSQSSIGTESFHSAQSVA |
| P | Q888Y1 | Pseudomonas syringae pv. tomato | HopR1 | MVKVTSSGFTANPLSHHADSVSPAN |
| P | Q87W07 | Pseudomonas syringae pv. tomato | HopI1 | MINLTHIASSLARAALSDSTKPKME |
| P | Q08370 | Pseudomonas syringae pv. syringae | HrmA | MNPIHARFSSVEALRHSNVDIQAIK |
| P | Q87WF7 | Pseudomonas syringae pv. tomato | HopT1-2 | MIKTVSDNSIPGTYGIAFTRVDTAA |
| P | Q87X57 | Pseudomonas syringae pv. tomato | HopE1 | MNRVSGSSSATWQAVNDLVEQVSER |
| P | Q87W42 | Pseudomonas syringae pv. tomato | HopG1 | MQIKNSHLYSASRMVQNTFNASPKM |
| P | Q88A09 | Pseudomonas syringae pv. tomato | HopH1 | MITPSRYPGIYIAPLSNEPTAAHTF |
| P | Q881L7 | Pseudomonas syringae pv. tomato | HopL1 | MTTLTTRQIQLAHAWTSVHTGAGLA |
| P | Q9K2L5 | Pseudomonas syringae pv. phaseolicola |  | MGNICNSGGVSRTYSPPTSPVYGSG |
| P | Q87W46 | Pseudomonas syringae pv. tomato | HopV1 | MRFDAARGQKPKAPMDAPSSLRLRA |
| P | Q88AB8 | Pseudomonas syringae pv. tomato | HopAS1 | MTLRINTRSATPVVPLETGSTSQPT |
| P | Q7PC62 | Pseudomonas syringae pv. syringae (strain B728a) | HopAE1 | MMPSQITRSSHSSLPEVAPASGDAA |
| P | Q7PC42 | Pseudomonas syringae pv. syringae (strain B728a) | HopAC1 | MTQTPPSLDFNLSTPSPVPMTPSDT |
| P | Q52530 | Pseudomonas syringae pv. phaseolicola | AvrD | MQDLSFSTIENHLGPAKDRFFGDGF |
| P | Q9L6W4 | Pseudomonas syringae pv. tomato | HopB1 | MRPVGGPAPGYYPPTYEAERPTAQA |
| P | Q9F3T4 | Pseudomonas syringae pv. pisi | AvrPpiC2 | MTIVSGHIGKHPSLTTVQAGSSASV |
| P | Q52394 | Pseudomonas syringae pv. phaseolicola | AvrPphE | MRIHSAGHSLPAPGPSVETTEKAVQ |
| P | Q48B61 | Pseudomonas syringae pv. phaseolicola (strain 1448A / Race 6) | HopAB1 | MPGINGAGPSNFFWQWRTDGEPVTE |
| P | Q888W0 | Pseudomonas syringae pv. tomato | HopAI1 | MLALKLNTSIAQAPLKKNAEAELRH |
| P | Q7PC45 | Pseudomonas syringae pv. syringae (strain B728a) | HopAG1 | MFIASPKTNVQECLMINPVKHNFSH |
| P | P11437 | Pseudomonas syringae pv. glycinea | AvrA | MWNVSKSSNNLGAYKLPLEAQTPPE |
| P | Q52432 | Pseudomonas syringae | AvrRps4 | MTRISTSSVNSSFSYSAPAEEAQNR |
| P | Q48BE0 | Pseudomonas syringae pv. phaseolicola (strain 1448A / Race 6) | HopD1 | MNPLRSIQHNITTPPISGGQPLDAV |
| P | Q52389 | Pseudomonas syringae |  | MQSPSIHRNTGSIIQPTVTPDARAA |
| P | Q9JP32 | Pseudomonas syringae pv. tomato | HopN1 | MYIQQSGAQSGVAAKTQHDKPSSLS |
| P | Q87W65 | Pseudomonas syringae pv. tomato | HopAD1 | MLIGHSLHHMRPTAVDSSLPTSATS |
| P | Q87XS5 | Pseudomonas syringae pv. tomato | HopAK1 | MNTINRNIYPVSGISAQDAPVQTDQ |
| P | Q9L6W3 | Pseudomonas syringae pv. tomato | HrpK | MRISSSPFVIVNQPTPGELALAVES |
| T | Q663I2 | Yersinia pseudotuberculosis | YscH | MTVTLNRGSITSLMSSSQAVSTLQP |
| T | A6M3R1 | Yersinia pestis CA88-4125 | YscL | MSQTCQTGYAYMQPFVQIIPSNLSL |
| T/C | P94517 | Bacillus subtilis | YscB | MNKLIQLALFFTLMLTGCSNSSTSS |
| T | B0HZP5 | Yersinia pestis biovar Antiqua str. E1979001 | YscR | MIQLPDEINLIIVLSLLTLLPLISV |
| T | A6M3R2 | Yersinia pestis CA88-4125 | YscK | MMENYITSFQLRFCPAAYLHLEQLP |
| T | B0HZP9 | Yersinia pestis biovar Antiqua str. E1979001 | YscN | MLSLDQIPHHIRHGIVGSRLIQIRG |
| T | P94518 | Bacillus subtilis | YscA | MLLLVIYIQFNNYSTQYRIFSQGRL |
| T | A6M3T7 | Yersinia pestis CA88-4125 | YscY | MNITLTKRQQEFLLLNGWLQLQCGH |
| T | A9K514 | Burkholderia mallei ATCC 10399 | BsaW | MANNEIALIVLLTAATLVPFVVAAG |
| T | A9R9K1 | Yersinia pestis bv. Antiqua (strain Angola) | YscH | MTVTLNRGSITSLMSSSQAVSTLQP |
| T | B0HZP3 | Yersinia pestis biovar Antiqua str. E1979001 | YscT | MIADLIQRPLLTYTLLLPRFMACFV |
| T | A9K4S2 | Burkholderia mallei ATCC 10399 | SctR | MVQFNDITGLLIAVLVMSMVPFIAM |
| T | B4TH61 | Salmonella enterica subsp. enterica serovar Heidelberg str. SL476 | EpaP | MSLPDSPLQLIGILFLLSILPLIIV |
| T | A9R9K6 | Yersinia pestis bv. Antiqua (strain Angola) | YscM | MKINTLQSLINQQITQVGHGGQAGR |
| T | Q1MQX2 | Lawsonia intracellularis (strain PHE/MN1-00) | YscU | MSGDSGDKTEPPTPKKLREAREQGD |
| T | B0HZN2 | Yersinia pestis biovar Antiqua str. E1979001 | YscJ | MKVKTSLSTLILILFLTGCKVDLYT |
| T | Q2SC30 | Hahella chejuensis (strain KCTC 2396) | YscT | MMTHLMSDTLTLFMFTVPRLLAAMS |
| T | B0HNK1 | Yersinia pestis biovar Antiqua str. B42003004 | YscU | MSGEKTEQPTPKKIRDARKKGQVAK |
| T | Q2SC35 | Hahella chejuensis (strain KCTC 2396) | YscO | MLSALADIKKLRERNAQAEALRKRQ |
| T | B0HZP6 | Yersinia pestis biovar Antiqua str. E1979001 | YscQ | MSLLTLPQAKLSELSLRQRLSHYQQ |
| T | Q2SC33 | Hahella chejuensis (strain KCTC 2396) | YscQ | MTLDNLICARRRPFALQLPEHIAQF |
| T | B0HZP7 | Yersinia pestis biovar Antiqua str. E1979001 | YscP | MNKITTRSPLEPEYQPLGKPHHALQ |
| T | Q2SC31 | Hahella chejuensis (strain KCTC 2396) | YscS | MSEAEVIHYASQLLMLVLVLSMPTV |
| T | A6M3T7 | Yersinia pestis CA88-4125 | YscY | MNITLTKRQQEFLLLNGWLQLQCGH |
| T | A6M3R1 | Yersinia pestis CA88-4125 | YscL | MSQTCQTGYAYMQPFVQIIPSNLSL |
| T | A3NKX4 | Burkholderia pseudomallei (strain 668) | YscR | MVQFSDITGLLLVVIAISLLPFIAM |
| T/C | B0HZP0 | Yersinia pestis biovar Antiqua str. E1979001 | YscB | MQNLLKNLAASLGRKPFVADKQGVY |
| T | B0HNJ2 | Yersinia pestis biovar Antiqua str. B42003004 | YscI | MPNIEIAQADEVIITTLEELGPAEP |
| T | Q1MQX3 | Lawsonia intracellularis (strain PHE/MN1-00) | YscT | MNYDQTIGALGVYDHFIAFLIGTPR |
| T | B0HZP4 | Yersinia pestis biovar Antiqua str. E1979001 | YscS | MSQGDIIHFTSQALWLVLVLSMPPV |
| T | Q1MQX4 | Lawsonia intracellularis (strain PHE/MN1-00) | YscS | METTTMTYTAKALYLVLVLSMPPIL |
| T | Q254G9 | Chlamydophila felis (strain Fe/C-56) | YscU | MGEKTEKATPKRLRDARKKGQVAKS |
| T | B0HZP4 | Yersinia pestis biovar Antiqua str. E1979001 | YscS | MSQGDIIHFTSQALWLVLVLSMPPV |
| T | Q2T727 | Burkholderia thailandensis (strain E264 / ATCC 700388 / DSM 13276 / CIP 106301) | YscF | MSNPPTPLLTDYEWSGYLTGIGRAF |
| T | Q1MQX5 | Lawsonia intracellularis (strain PHE/MN1-00) | YscV | MSLFAKAQSTVGVITRNNDITMVLL |
| T | B0HNJ2 | Yersinia pestis biovar Antiqua str. B42003004 | YscI | MPNIEIAQADEVIITTLEELGPAEP |
| T | Q2SC29 | Hahella chejuensis (strain KCTC 2396) | YscU | MSDEKTEKPTPKKLRDARKKGQVAH |
| T | A9R9K6 | Yersinia pestis bv. Antiqua (strain Angola) | YscM | MKINTLQSLINQQITQVGHGGQAGR |
| T | B0HZP7 | Yersinia pestis biovar Antiqua str. E1979001 | YscP | MNKITTRSPLEPEYQPLGKPHHALQ |
| T | Q252Q1 | Chlamydophila felis (strain Fe/C-56) | YscC | MKIVTSNIGRKILQVINKKKGKIGI |
| T | B0HZP6 | Yersinia pestis biovar Antiqua str. E1979001 | YscQ | MSLLTLPQAKLSELSLRQRLSHYQQ |
| T | B0HZP3 | Yersinia pestis biovar Antiqua str. E1979001 | YscT | MIADLIQRPLLTYTLLLPRFMACFV |
| T/C | B0HZP0 | Yersinia pestis biovar Antiqua str. E1979001 | YscB | MQNLLKNLAASLGRKPFVADKQGVY |
| T | B0HNK1 | Yersinia pestis biovar Antiqua str. B42003004 | YscU | MSGEKTEQPTPKKIRDARKKGQVAK |
| T | B0HNK1 | Yersinia pestis biovar Antiqua str. B42003004 | YscU | MSGEKTEQPTPKKIRDARKKGQVAK |
| T | B0HZN2 | Yersinia pestis biovar Antiqua str. E1979001 | YscJ | MKVKTSLSTLILILFLTGCKVDLYT |
| T | B0HNK7 | Yersinia pestis biovar Antiqua str. B42003004 | YscO | MIRRLHRVKVLRVERAEKAIKTQQA |
| T | B0HZP5 | Yersinia pestis biovar Antiqua str. E1979001 | YscR | MIQLPDEINLIIVLSLLTLLPLISV |
| T | A9R9K6 | Yersinia pestis bv. Antiqua (strain Angola) | YscM | MKINTLQSLINQQITQVGHGGQAGR |
| T | B0HZN2 | Yersinia pestis biovar Antiqua str. E1979001 | YscJ | MKVKTSLSTLILILFLTGCKVDLYT |
| T | B0HZP5 | Yersinia pestis biovar Antiqua str. E1979001 | YscR | MIQLPDEINLIIVLSLLTLLPLISV |
| T | P40295 | Yersinia pestis | YscP | MNKITTRSPLEPEYQPLGKPHHALQ |
| T | B0HZQ3 | Yersinia pestis biovar Antiqua str. E1979001 | YscX | MSRIITAPHIGIEKLSAISLEELSC |
| T | Q2SC32 | Hahella chejuensis (strain KCTC 2396) | YscR | MTDIQAIPLIGTLVLLSLIPFIAIM |
| T | A6M3R1 | Yersinia pestis CA88-4125 | YscL | MSQTCQTGYAYMQPFVQIIPSNLSL |
| T | A6M3R2 | Yersinia pestis CA88-4125 | YscK | MMENYITSFQLRFCPAAYLHLEQLP |
| T | A9R9K1 | Yersinia pestis bv. Antiqua (strain Angola) | YscH | MTVTLNRGSITSLMSSSQAVSTLQP |
| T | B0HZP3 | Yersinia pestis biovar Antiqua str. E1979001 | YscT | MIADLIQRPLLTYTLLLPRFMACFV |
| T | B0HZP4 | Yersinia pestis biovar Antiqua str. E1979001 | YscS | MSQGDIIHFTSQALWLVLVLSMPPV |
| T | B0HZP6 | Yersinia pestis biovar Antiqua str. E1979001 | YscQ | MSLLTLPQAKLSELSLRQRLSHYQQ |
| T | B0HNJ2 | Yersinia pestis biovar Antiqua str. B42003004 | YscI | MPNIEIAQADEVIITTLEELGPAEP |
| T | Q2SC36 | Hahella chejuensis (strain KCTC 2396) | YscN | MSQLSHVLSDLRSAIAEVQPVGLRG |
| C | P61380 | Yersinia pestis | SycN | MSWIEPIISHFCQDLGVPTSSPLSP |
| C | Q9ZGW6 | Yersinia pestis | SycT | MQTTFTELMQQLFLKLGLNH QVNE |
| C | O34021 | Chlamydophila caviae | SycE | MQNQFEQLLESLGTKLNTSLVPDKN |
