# Supplementary material for: Sequence-Based Prediction of Type III Secreted Proteins
Source: PLoS Pathog. 2009 Apr 24;5(4):e1000376. doi: 10.1371/journal.ppat.1000376 (PMC2669295; doi:10.1371/journal.ppat.1000376)
Supplement: Table S2 — Orthologous groups of effector proteins. This table comprises effector proteins with individual experimental evidence for type III mediated transport which can be clustered into orthologous groups (clustered by homology and manual inspection). A sequence is added to a cluster, if it has at least Sratio> = 0.15 to one other cluster member. Sratio is computed as alignment-score/selfscore. (0.08 MB DOC) [file ppat.1000376.s005.doc]

Table S2. Orthologous groups of effector proteins

This table comprises effector proteins with individual experimental evidence for type III mediated transport which can be clustered into orthologous groups (clustered by homology and manual inspection). A sequence is added to a cluster, if it has at least S_ratio_>=0.15 to one other cluster member. S_ratio_ is computed as alignment-score/selfscore.

| **Cluster Name** | **Accession** | **Organism** | **Name** |
| --- | --- | --- | --- |
|  |  |  |  |
| YopE  (2 members) | A6M3N5* | Yersinia pestis CA88-4125 | YopE |
|  | Q7BRY7 | Yersinia enterocolitica | YopE |
|  |  |  |  |
| YopH  (2 members) | A9R9K8 | Yersinia pestis bv. Antiqua (strain Angola) | YopH |
|  | Q7BRY8* | Yersinia enterocolitica | YopH |
|  |  |  |  |
| YopK  (3 members) | B0A3S4 | Yersinia pestis biovar Orientalis str. F1991016 | YopK |
|  | Q56935* | Yersinia pseudotuberculosis | YopK |
|  | Q7BS06 | Yersinia enterocolitica | YopQ |
|  |  |  |  |
| YopJ  (2 members) | B0HNN9* | Yersinia pestis biovar Antiqua str. B42003004 | YopJ |
|  | Q93KQ5 | Yersinia enterocolitica | YopP |
|  |  |  |  |
| YopM  (4 members) | A6M3U5* | Yersinia pestis CA88-4125 | YopM |
|  | Q663L9 | Yersinia pseudotuberculosis | YopM |
|  | Q93KU8 | Yersinia enterocolitica | YopM |
|  | Q9RPH0 | Salmonella typhimurium | SspH2 |
|  |  |  |  |
| YopT  (4 members) | B0A3S3 | Yersinia pestis biovar Orientalis str. F1991016 | YopT |
|  | Q93RN4* | Yersinia pseudotuberculosis | YopT |
|  | P27475 | Yersinia enterocolitica | YopT |
|  | P0C2N1 | Yersinia enterocolitica | YopT1 |
|  |  |  |  |
| YopO/YpkA  (4 members) | A9ZFE7 | Yersinia pestis biovar Orientalis str. IP275 | YopO |
|  | Q05608* | Yersinia pseudotuberculosis | YpkA |
|  | Q56921 | Yersinia enterocolitica | YpkA |
|  | O85239 | Yersinia enterocolitica | YopO |
|  |  |  |  |
| YscH  (3 members) | A9ZER0 | Yersinia pestis biovar Orientalis str. IP275 | YscH |
|  | Q663I2 | Yersinia pseudotuberculosis | YscH |
|  | Q7BRZ4* | Yersinia enterocolitica | YscH |
|  |  |  |  |
| CopN  (2 members) | O34020* | Chlamydophila caviae | CopN |
|  | Q9Z8L4 | Chlamydophila pneumoniae | LcrE |
|  |  |  |  |
| TARP  (3 members) | Q824H6* | Chlamydophila caviae | TARP |
|  | O84462 | Chlamydia trachomatis | TARP |
|  | Q9Z7Y1 | Chlamydophila pneumoniae | TARP |
|  |  |  |  |
| IncB  (2 members) | O84235* | Chlamydia trachomatis | IncB |
|  | Q9Z8P7 | Chlamydophila pneumoniae | IncB |
|  |  |  |  |
| IncC  (5 members) | O30783* | Chlamydophila caviae | IncC |
|  | O84236 | Chlamydia trachomatis | IncC |
|  | Q9Z8P6 | Chlamydophila pneumoniae | IncC |
|  | O84119 | Chlamydia trachomatis | IncF |
|  | Q3KMQ1 | Chlamydia trachomatis (strain A/HAR-13 / ATCC VR-571B) | IncG |
|  |  |  |  |
| SopE  (2 members) | O52623* | Salmonella typhimurium | SopE |
|  | Q7CQD4 | Salmonella typhimurium | SopE2 |
|  |  |  |  |
| EspF/TccP  (3 members) | Q7DB85* | Escherichia coli O157:H7 | EspF |
|  | Q8X2D5 | Escherichia coli O157:H7 | TccP |
|  | A2A0X3 | Escherichia coli O157:H- | TccP2 |

* Protein which represents the orthologous group for training and testing purposes
