# Supplementary material for: Sequence-Based Prediction of Type III Secreted Proteins
Source: PLoS Pathog. 2009 Apr 24;5(4):e1000376. doi: 10.1371/journal.ppat.1000376 (PMC2669295; doi:10.1371/journal.ppat.1000376)
Supplement: Table S3 — Groups of co-evolving effector and TTSS proteins and examples of co-localized effector proteins and chaperones based on the STRING database. For each group of co-evolving effector and TTSS proteins, gene names of the members are given. The right column indicates, whether the orthologous group comprises effectors, TTSS proteins or TTSS related chaperones. A gene is added to a cluster, if the score of a genomic context method to another member derived from STRING exceeds 0.5. In the last section, examples of co-localized effectors and chaperones are listed. (0.05 MB DOC) [file ppat.1000376.s006.doc]

Table S3. Groups of co-evolving effector and TTSS proteins and examples of co-localized effector proteins and chaperones based on the STRING database

For each group of co-evolving effector and TTSS proteins, gene names of the members are given. The right column indicates, whether the orthologous group comprises effectors, TTSS proteins or TTSS related chaperones. A gene is added to a cluster, if the score of a genomic context method to another member derived from STRING exceeds 0.5. In the last section, examples of co-localized effectors and chaperones are listed.

| **Group of co-evolving proteins** | **Effector or TTSS** |
| --- | --- |
| **Group I**  (Evidence exclusively by fusion events) | |
| YpkA | Effector |
| SopA | Effector |
| YopT | Effector |
| SspH2 | Effector |
| **Group II**  (Evidence by conserved neighbourhood for all interactions, supported by phylogenetic profile in some cases) | |
| EspD | Effector |
| Span | Effector |
| SopB | Effector |
| SipA | Effector |
| YscH | Effector |
| YscN | TTSS |
| YscL | TTSS |
| YscU | TTSS |
| YscC | TTSS |
| YscQ | TTSS |
| YscJ | TTSS |
| YscV | TTSS |
| YscR | TTSS |
| YscT | TTSS |
| YscU | TTSS |
| YscS | TTSS |
| YscB | TTSS/Chaperone |
| YscI | TTSS |
| YscF | TTSS |
| YscY | TTSS |

| **Examples of effectors which are co-localized with TTSS chaperones**  Evidence exclusively by conserved neighbourhood in all examples | |
| --- | --- |
| **Effector** | **Chaperone** |
| YopN | SycN |
| YopT | SycT |
| CopN | SycE |
