# Supplementary material for: Sequence-Based Prediction of Type III Secreted Proteins
Source: PLoS Pathog. 2009 Apr 24;5(4):e1000376. doi: 10.1371/journal.ppat.1000376 (PMC2669295; doi:10.1371/journal.ppat.1000376)
Supplement: Table S4 — Number of genomic neighbours of known effectors, number of non-neighbours and their association to the TTSS. For all known effectors from Table S1, genomic neighbours have been determined for a certain distance upstream and downstream on the chromosome or plasmid. These neighbours and the remaining, non-neighboured proteins of the genomes have been distinguished by their association to the TTSS. Components of the TTSS are enriched in the neighbourhood of effectors. The statistical significance of this enrichment has been determined using the t-Test. The most significant enrichment of TTSS components in the genomic neighbourhood of effectors can be observed within the range of 30 neighbours up- and downstream (marked in red). (0.04 MB DOC) [file ppat.1000376.s007.doc]

Table S4. Number of genomic neighbours of known effectors, number of non-neighbours and their association to the TTSS

For all known effectors from Table S1, genomic neighbours have been determined for a certain distance upstream and downstream on the chromosome or plasmid. These neighbours and the remaining, non-neighboured proteins of the genomes have been distinguished by their association to the TTSS. Components of the TTSS are enriched in the neighbourhood of effectors. The statistical significance of this enrichment has been determined using the t-Test. The most significant enrichment of TTSS components in the genomic neighbourhood of effectors can be observed within the range of 30 neighbours up- and downstream (marked in red).

| Number of neighbours in each direction | Number of neighbours which are TTSS components | Number of neighbours which are no TTSS components | Number of non-neighbours which are TTSS components | Number of non-neighbours which are no TTSS components | p-Value (t-Test) |
| --- | --- | --- | --- | --- | --- |
| 10 | 51 | 1264 | 211 | 59799 | 3.04E-033 |
| 20 | 79 | 2210 | 183 | 58853 | 1.31E-048 |
| 30 | 94 | 3046 | 168 | 58017 | 2.44E-053 |
| 40 | 96 | 3844 | 166 | 57219 | 8.31E-047 |
| 50 | 96 | 4626 | 166 | 56437 | 3.75E-040 |
| 60 | 96 | 5354 | 166 | 55709 | 4.86E-035 |
| 70 | 96 | 6054 | 166 | 55009 | 7.39E-031 |
| 80 | 96 | 6754 | 166 | 54309 | 3.07E-027 |
| 90 | 96 | 7407 | 166 | 53656 | 2.83E-024 |
