# Supplementary material for: Sequence-Based Prediction of Type III Secreted Proteins
Source: PLoS Pathog. 2009 Apr 24;5(4):e1000376. doi: 10.1371/journal.ppat.1000376 (PMC2669295; doi:10.1371/journal.ppat.1000376)
Supplement: Table S5 — Enrichment of KEGG orthologous groups within the genomic neighbourhood of known effectors. This table lists KEGG orthologous groups (KO), which are significantly enriched (Bonferroni-corrected t-Test p-Value<0.05) within 30 neighbours up- and downstream of known effectors. (0.03 MB DOC) [file ppat.1000376.s008.doc]

Table S5. Enrichment of KEGG orthologous groups within the genomic neighbourhood of known effectors

This table lists KEGG orthologous groups (KO), which are significantly enriched (Bonferroni-corrected t-Test p-Value < 0.05) within 30 neighbours up- and downstream of known effectors.

| KEGG orthologous group (KO) | Definition | Adjusted p-Value for the enrichment of the KO within 30 neighbours up- and downstream of known effectors |
| --- | --- | --- |
| K03229 | type III secretion protein SctU | 2.11E-005 |
| K03230 | type III secretion protein SctV | 3.60E-005 |
| K03225 | type III secretion protein SctQ | 3.12E-004 |
| K04058 | type III secretion protein SctW | 1.04E-002 |
| K03220 | type III secretion protein SctD | 2.86E-002 |
| K03227 | type III secretion protein SctS | 2.90E-002 |
| K03228 | type III secretion protein SctT | 4.15E-002 |
