# Supplementary material for: Sequence-Based Prediction of Type III Secreted Proteins
Source: PLoS Pathog. 2009 Apr 24;5(4):e1000376. doi: 10.1371/journal.ppat.1000376 (PMC2669295; doi:10.1371/journal.ppat.1000376)
Supplement: Table S6 — Known effectors and their genomic neighbourhood to TTSS components. The genomic neighbourhood (30 genes up- and downstream) to TTSS components has been evaluated for all known effectors, except on Yersinia pestis KIM due to the absence of the plasmid pCD1 from the KEGG database. The number of effectors which are neighboured to at least one TTSS component is given in the middle column, the remaining effectors are summarized in the right column. (0.04 MB DOC) [file ppat.1000376.s009.doc]

Table S6. Known effectors and their genomic neighbourhood to TTSS components

The genomic neighbourhood (30 genes up- and downstream) to TTSS components has been evaluated for all known effectors, except on Yersinia pestis KIM due to the absence of the plasmid pCD1 from the KEGG database. The number of effectors which are neighboured to at least one TTSS component is given in the middle column, the remaining effectors are summarized in the right column.

| Genome | Number of effectors in the genomic neighbourhood of TTSS components | Number of effectors not in the genomic neighbourhood of TTSS components |
| --- | --- | --- |
| Chlamydophila caviae GPIC | 2 | 3 |
| Chlamydophila pneumoniae CWL029 | 2 | 5 |
| Chlamydia trachomatis A/HAR-13 (serovar A) | 2 | 0 |
| Chlamydia trachomatis D/UW-3/CX (serovar D) | 3 | 3 |
| Escherichia coli O157:H7 EDL933 (EHEC) | 5 | 2 |
| Escherichia coli O127:H6 E2348/69 | 1 | 0 |
| Escherichia coli O157:H7 Sakai (EHEC) | 0 | 2 |
| Pseudomonas syringae pv. syringae B728a | 0 | 3 |
| Pseudomonas syringae pv. phaseolicola 1448A | 1 | 4 |
| Pseudomonas syringae pv. tomato DC3000 | 5 | 19 |
| Salmonella enterica subsp. enterica serovar Choleraesuis | 0 | 1 |
| Salmonella enterica subsp. enterica serovar Schwarzengrund CVM19633 | 0 | 1 |
| Salmonella typhimurium LT2 | 3 | 5 |
| Yersinia enterocolitica subsp. enterocolitica 8081 | 9 | 0 |
| Yersinia pestis Antiqua | 1 | 0 |
| Yersinia pseudotuberculosis IP32953 | 8 | 0 |
