# Supplementary material for: Sequence-Based Prediction of Type III Secreted Proteins
Source: PLoS Pathog. 2009 Apr 24;5(4):e1000376. doi: 10.1371/journal.ppat.1000376 (PMC2669295; doi:10.1371/journal.ppat.1000376)
Supplement: Table S7 — Performance of the classifiers using the C-terminal end. To prove the concept of the N-terminal signal peptide, C-termini should have no predictive power. The performance for several classifiers has been evaluated using exactly the same feature selection, training and test procedure as used for the N-termini. 5 runs with different negative sets have been performed. (0.03 MB DOC) [file ppat.1000376.s010.doc]

Table S7. Performance of the classifiers using the C-terminal end

To prove the concept of the N-terminal signal peptide, C-termini should have no predictive power. The performance for several classifiers has been evaluated using exactly the same feature selection, training and test procedure as used for the N-termini. 5 runs with different negative sets have been performed.

| **Algorithm** | **AUC** | **Standard Deviation** |
| --- | --- | --- |
| Perceptron | 0.54 | 0.04 |
| 1 Nearest Neighbour | 0.48 | 0.02 |
| Logistic Regression | 0.52 | 0.02 |
| Support Vector machine | 0.49 | 0.02 |
| Naïve Bayes. Multinomial | 0.55 | 0.03 |
| Naïve Bayes Complement | 0.53 | 0.03 |
| Naïve Bayes | 0.52 | 0.04 |
