# Supplementary material for: Sequence-Based Prediction of Type III Secreted Proteins
Source: PLoS Pathog. 2009 Apr 24;5(4):e1000376. doi: 10.1371/journal.ppat.1000376 (PMC2669295; doi:10.1371/journal.ppat.1000376)
Supplement: Table S8 — Prediction results with EffectiveT3 trained without a certain taxonomic sub-set. EffectiveT3 has been trained without the positive and negative samples from the excluded taxonomic groups listed in this table. Testing EffectiveT3 on these effectors (E) and randomly chosen negative samples (R) resulted in true positive (+E), false negative (−E), false positive (+R) and true negative (−R) predictions. (0.35 MB DOC) [file ppat.1000376.s011.doc]

Table S8. Prediction results with EffectiveT3 trained without a certain taxonomic sub-set

EffectiveT3 has been trained without the positive and negative samples from the excluded taxonomic groups listed in this table. Testing EffectiveT3 on these effectors (E) and randomly chosen negative samples (R) resulted in true positive (+E), false negative (-E), false positive (+R) and true negative (-R) predictions.

| **Protein** | **Genome** | **Prediction** | **Effector / non-effector** |
| --- | --- | --- | --- |
| **Excluded group: Chlamydia** | | | |
| tarP | Chlamydophila caviae | + | E |
| incA | Chlamydophila caviae | + | E |
| incA | Chlamydia trachomatis (strain A/HAR-13 / ATCC VR-571B) | + | E |
| incB | Chlamydia trachomatis | + | E |
| IncC | Chlamydophila caviae | + | E |
| Q9Z9F5 | Chlamydophila pneumoniae | + | E |
| Q9Z7W9 | Chlamydophila pneumoniae | + | E |
| incA | Chlamydophila pneumoniae | + | E |
| copN | Chlamydophila caviae | - | E |
| incD | Chlamydia trachomatis | - | E |
| incE | Chlamydia trachomatis | - | E |
| Q255I6 | Chlamydophila felis Fe/C-56 | + | R |
| Q9JQE1 | Chlamydophila pneumoniae | + | R |
| Q9Z9F6 | Chlamydophila pneumoniae | + | R |
| Q9Z5Q4 | Chlamydophila pneumoniae | + | R |
| Q255M0 | Chlamydophila felis Fe/C-56 | + | R |
| Q5L6M8 | Chlamydophila abortus | + | R |
| O84340 | Chlamydia trachomatis | - | R |
| Q3KMC6 | Chlamydia trachomatis A/HAR-13 | - | R |
| Q823K0 | Chlamydophila caviae | - | R |
| O84402 | Chlamydia trachomatis | - | R |
| Q822Y3 | Chlamydophila caviae | - | R |
| Q5L681 | Chlamydophila abortus | - | R |
| O84859 | Chlamydia trachomatis | - | R |
| Q9Z913 | Chlamydophila pneumoniae | - | R |
| Q9K2D4 | Chlamydophila pneumoniae | - | R |
| Q3KLB6 | Chlamydia trachomatis A/HAR-13 | - | R |
| O84835 | Chlamydia trachomatis | - | R |
| Q3KKY9 | Chlamydia trachomatis A/HAR-13 | - | R |
| Q9JQ87 | Chlamydophila pneumoniae | - | R |
| Q7DET0 | Chlamydophila pneumoniae | - | R |
| Q9Z8L9 | Chlamydophila pneumoniae | - | R |
| Q3KLV9 | Chlamydia trachomatis A/HAR-13 | - | R |
| Q252X3 | Chlamydophila felis Fe/C-56 | - | R |
| Q821V0 | Chlamydophila caviae | - | R |
| Q9JQI0 | Chlamydophila pneumoniae | - | R |
| Q9Z6M5 | Chlamydophila pneumoniae | - | R |
| Q252M1 | Chlamydophila felis Fe/C-56 | - | R |
| Q9Z7P3 | Chlamydophila pneumoniae | - | R |
| Q824A6 | Chlamydophila caviae | - | R |
| Q3KL78 | Chlamydia trachomatis A/HAR-13 | - | R |
| Q5L593 | Chlamydophila abortus | - | R |
| O84860 | Chlamydia trachomatis | - | R |
| **Excluded group: Escherichia** | | | |
| tir | Escherichia coli | + | E |
| espA | Escherichia coli | + | E |
| espD | Escherichia coli O157:H7 | + | E |
| tccP | Escherichia coli O157:H7 | + | E |
| NleB | Escherichia coli O157:H7 str. EC4196 | + | E |
| st47 | Escherichia coli | + | E |
| espF | Escherichia coli O157:H7 | - | E |
| EspG | Escherichia coli | - | E |
| P78279 | Escherichia coli K-12 | + | R |
| Q2M7J9 | Escherichia coli K-12 | + | R |
| A7ZW83 | Escherichia coli HS | + | R |
| B1IY66 | Escherichia coli ATCC 8739 | - | R |
| P78264 | Escherichia coli O6 | - | R |
| Q7ABY5 | Escherichia coli O157:H7 | - | R |
| A8A7Q7 | Escherichia coli HS | - | R |
| A7ZL97 | Escherichia coli E24377A | - | R |
| A7ZVR5 | Escherichia coli E24377A | - | R |
| A8A5C6 | Escherichia coli HS | - | R |
| P78231 | Escherichia coli O157:H7 | - | R |
| Q8FAX1 | Escherichia coli O6 | - | R |
| Q2M7N9 | Escherichia coli K-12 | - | R |
| Q2MA14 | Escherichia coli K-12 | - | R |
| Q2M7G4 | Escherichia coli K-12 | - | R |
| B1LN92 | Escherichia coli SMS-3-5 | - | R |
| **Excluded group: Salmonella** | | | |
| sipA | Salmonella typhimurium | + | E |
| sspH2 | Salmonella typhimurium | + | E |
| sifA | Salmonella typhimurium | + | E |
| sopA | Salmonella typhimurium | + | E |
| sopB | Salmonella choleraesuis | + | E |
| sopD | Salmonella typhimurium | + | E |
| spaN | Salmonella typhimurium | - | E |
| sopE | Salmonella typhimurium | - | E |
| sptP | Salmonella typhimurium | - | E |
| P26155 | Salmonella typhi | + | R |
| A9MPF3 | Salmonella enterica subsp. arizonae serovar 62:z4,z23:-- | + | R |
| Q8Z9D3 | Salmonella typhi | - | R |
| Q56120 | Salmonella typhimurium | - | R |
| Q8Z116 | Salmonella typhi | - | R |
| Q5PJE8 | Salmonella enterica subsp. enterica serovar Paratyphi A | - | R |
| Q5PDA1 | Salmonella enterica subsp. enterica serovar Paratyphi A | - | R |
| Q9CL31 | Yersinia pestis | - | R |
| A9MNX0 | Salmonella enterica subsp. arizonae serovar 62:z4,z23:-- | - | R |
| A9MHF5 | Salmonella enterica subsp. arizonae serovar 62:z4,z23:-- | - | R |
| Q9L6P6 | Salmonella typhi | - | R |
| Q9L4I4 | Salmonella typhimurium | - | R |
| Q7C7H4 | Salmonella typhi | - | R |
| Q5PLD3 | Salmonella enterica subsp. enterica serovar Paratyphi A | - | R |
| Q5PK71 | Salmonella enterica subsp. enterica serovar Paratyphi A | - | R |
| Q5PJG1 | Salmonella enterica subsp. enterica serovar Paratyphi A | - | R |
| Q8Z9L4 | Salmonella typhi | - | R |
| Q8Z3A4 | Salmonella typhi | - | R |
| **Excluded group: Yersinia** | | | |
| yopE | Yersinia pestis CA88-4125 | + | E |
| yopH | Yersinia enterocolitica | + | E |
| yopJ | Yersinia pestis biovar Antiqua str. B42003004 | + | E |
| yopM | Yersinia pestis CA88-4125 | + | E |
| yopT | Yersinia pseudotuberculosis | + | E |
| yscH | Yersinia pestis bv. Antiqua (strain Angola) | + | E |
| yopK | Yersinia pseudotuberculosis | - | E |
| ypkA | Yersinia pseudotuberculosis | - | E |
| Q0WCE9 | Yersinia pestis | + | R |
| Q0WH55 | Yersinia pestis | + | R |
| A1JS52 | Yersinia enterocolitica subsp. enterocolitica 8081 | - | R |
| Q0WJV3 | Yersinia pestis | - | R |
| Q1C3K9 | Yersinia pestis Antiqua | - | R |
| Q667Q0 | Yersinia pseudotuberculosis | - | R |
| Q667Y4 | Yersinia pseudotuberculosis | - | R |
| Q1CNM8 | Yersinia pestis Nepal516 | - | R |
| A7FNN8 | Yersinia pseudotuberculosis IP 31758 | - | R |
| Q0WJM6 | Yersinia pestis | - | R |
| A1JIH6 | Yersinia enterocolitica subsp. enterocolitica 8081 | - | R |
| Q1C133 | Yersinia pestis Antiqua | - | R |
| A7FH41 | Yersinia pseudotuberculosis IP 31758 | - | R |
| Q93KR4 | Yersinia enterocolitica subsp. enterocolitica 8081 | - | R |
| Q1CCU3 | Yersinia pestis Nepal516 | - | R |
| Q8D055 | Yersinia pestis | - | R |
| Q664V8 | Yersinia pseudotuberculosis | - | R |
| Q1C8B0 | Yersinia pestis Antiqua | - | R |
| **Excluded group: Pseudomonas (plant symbiont set)** | | | |
| avrB | Pseudomonas syringae pv. glycinea | + | E |
| hopQ1-1 | Pseudomonas syringae pv. tomato | + | E |
| avrRpm1 | Pseudomonas syringae pv. maculicola | + | E |
| hopAF1 | Pseudomonas syringae pv. tomato | + | E |
| hopY1 | Pseudomonas syringae pv. tomato | + | E |
| hopP1 | Pseudomonas syringae pv. tomato | + | E |
| HopPtoA1Pma | Pseudomonas syringae pv. maculicola | + | E |
| hopR1 | Pseudomonas syringae pv. tomato | + | E |
| hrmA | Pseudomonas syringae pv. syringae | + | E |
| hopT1-2 | Pseudomonas syringae pv. tomato | + | E |
| hopE1 | Pseudomonas syringae pv. tomato | + | E |
| hopG1 | Pseudomonas syringae pv. tomato | + | E |
| hopH1 | Pseudomonas syringae pv. tomato | + | E |
| hopL1 | Pseudomonas syringae pv. tomato | + | E |
| Q9K2L5 | Pseudomonas syringae pv. phaseolicola | + | E |
| hopAS1 | Pseudomonas syringae pv. tomato | + | E |
| hopAE1 | Pseudomonas syringae pv. syringae (strain B728a) | + | E |
| HopAC1 | Pseudomonas syringae pv. syringae (strain B728a) | + | E |
| hopB1 | Pseudomonas syringae pv. tomato | + | E |
| avrPpiC2 | Pseudomonas syringae pv. pisi | + | E |
| avrPphE | Pseudomonas syringae pv. phaseolicola | + | E |
| HopAG1 | Pseudomonas syringae pv. syringae (strain B728a) | + | E |
| avrA | Pseudomonas syringae pv. glycinea | + | E |
| avrRps4 | Pseudomonas syringae | + | E |
| hopD1 | Pseudomonas syringae pv. phaseolicola (strain 1448A / Race 6) | + | E |
| Q52389 | Pseudomonas syringae | + | E |
| hopN1 | Pseudomonas syringae pv. tomato | + | E |
| hopAD1 | Pseudomonas syringae pv. tomato | + | E |
| hopAK1 | Pseudomonas syringae pv. tomato | + | E |
| hrpK | Pseudomonas syringae pv. tomato | + | E |
| hopAJ1 | Pseudomonas syringae pv. tomato | - | E |
| hopAN1 | Pseudomonas syringae pv. tomato | - | E |
| hopI1 | Pseudomonas syringae pv. tomato | - | E |
| hopV1 | Pseudomonas syringae pv. tomato | - | E |
| avrD | Pseudomonas syringae pv. phaseolicola | - | E |
| hopAB1 | Pseudomonas syringae pv. phaseolicola (strain 1448A / Race 6) | - | E |
| hopAI1 | Pseudomonas syringae pv. tomato | - | E |
| Q87ZE0 | Pseudomonas syringae pv. tomato | + | R |
| Q4ZRE3 | Pseudomonas syringae pv. syringae B728a | + | R |
| Q87Y31 | Pseudomonas syringae pv. tomato | + | R |
| Q87WL6 | Pseudomonas syringae pv. tomato | + | R |
| Q4ZQC3 | Pseudomonas syringae pv. syringae B728a | + | R |
| Q48F62 | Pseudomonas syringae pv. phaseolicola 1448A | + | R |
| Q48FC1 | Pseudomonas syringae pv. phaseolicola 1448A | + | R |
| Q48NX0 | Pseudomonas syringae pv. phaseolicola 1448A | + | R |
| Q48QH6 | Pseudomonas syringae pv. phaseolicola 1448A | - | R |
| Q4ZR01 | Pseudomonas syringae pv. syringae B728a | - | R |
| Q48E57 | Pseudomonas syringae pv. phaseolicola 1448A | - | R |
| Q87VP0 | Pseudomonas syringae pv. tomato | - | R |
| Q48CL6 | Pseudomonas syringae pv. phaseolicola 1448A | - | R |
| Q4ZLP3 | Pseudomonas syringae pv. syringae B728a | - | R |
| Q4ZLW0 | Pseudomonas syringae pv. syringae B728a | - | R |
| Q87TS1 | Pseudomonas syringae pv. tomato | - | R |
| Q889X7 | Pseudomonas syringae pv. tomato | - | R |
| Q48H73 | Pseudomonas syringae pv. phaseolicola 1448A | - | R |
| Q4ZNH2 | Pseudomonas syringae pv. syringae B728a | - | R |
| Q4ZY49 | Pseudomonas syringae pv. syringae B728a | - | R |
| Q887Q8 | Pseudomonas syringae pv. tomato | - | R |
| Q4ZM52 | Pseudomonas syringae pv. syringae B728a | - | R |
| Q886L7 | Pseudomonas syringae pv. tomato | - | R |
| Q48DE6 | Pseudomonas syringae pv. phaseolicola 1448A | - | R |
| Q4ZNX2 | Pseudomonas syringae pv. syringae B728a | - | R |
| Q48PA6 | Pseudomonas syringae pv. phaseolicola 1448A | - | R |
| Q48PV6 | Pseudomonas syringae pv. phaseolicola 1448A | - | R |
| Q500N6 | Pseudomonas syringae pv. syringae B728a | - | R |
| Q885U7 | Pseudomonas syringae pv. tomato | - | R |
| Q87V73 | Pseudomonas syringae pv. tomato | - | R |
| Q886N7 | Pseudomonas syringae pv. tomato | - | R |
| Q885W4 | Pseudomonas syringae pv. tomato | - | R |
| Q4ZRL1 | Pseudomonas syringae pv. syringae B728a | - | R |
| Q87YF7 | Pseudomonas syringae pv. tomato | - | R |
| Q889Y2 | Pseudomonas syringae pv. tomato | - | R |
| Q48N66 | Pseudomonas syringae pv. phaseolicola 1448A | - | R |
| Q889C2 | Pseudomonas syringae pv. tomato | - | R |
| Q48E80 | Pseudomonas syringae pv. phaseolicola 1448A | - | R |
| Q884C0 | Pseudomonas syringae pv. tomato | - | R |
| Q500B6 | Pseudomonas syringae pv. syringae B728a | - | R |
| Q48EF6 | Pseudomonas syringae pv. phaseolicola 1448A | - | R |
| Q4ZMP8 | Pseudomonas syringae pv. syringae B728a | - | R |
| Q4ZNI5 | Pseudomonas syringae pv. syringae B728a | - | R |
| Q87VF4 | Pseudomonas syringae pv. tomato | - | R |
| Q885W6 | Pseudomonas syringae pv. tomato | - | R |
| Q4ZNR0 | Pseudomonas syringae pv. syringae B728a | - | R |
| Q87VJ8 | Pseudomonas syringae pv. tomato | - | R |
| Q48EV4 | Pseudomonas syringae pv. phaseolicola 1448A | - | R |
| Q4ZM56 | Pseudomonas syringae pv. syringae B728a | - | R |
| Q48GE1 | Pseudomonas syringae pv. phaseolicola 1448A | - | R |
| Q48NZ3 | Pseudomonas syringae pv. phaseolicola 1448A | - | R |
| Q48L46 | Pseudomonas syringae pv. phaseolicola 1448A | - | R |
| Q4ZVY2 | Pseudomonas syringae pv. syringae B728a | - | R |
| Q48PV0 | Pseudomonas syringae pv. phaseolicola 1448A | - | R |
| Q882K4 | Pseudomonas syringae pv. tomato | - | R |
| Q87VM6 | Pseudomonas syringae pv. tomato | - | R |
| Q48L54 | Pseudomonas syringae pv. phaseolicola 1448A | - | R |
| Q4ZX02 | Pseudomonas syringae pv. syringae B728a | - | R |
| Q48EF0 | Pseudomonas syringae pv. phaseolicola 1448A | - | R |
| Q889F3 | Pseudomonas syringae pv. tomato | - | R |
| Q888C7 | Pseudomonas syringae pv. tomato | - | R |
| Q4ZM61 | Pseudomonas syringae pv. syringae B728a | - | R |
| Q87Z79 | Pseudomonas syringae pv. tomato | - | R |
| Q88AK3 | Pseudomonas syringae pv. tomato | - | R |
| Q4ZN82 | Pseudomonas syringae pv. syringae B728a | - | R |
| Q48NK9 | Pseudomonas syringae pv. phaseolicola 1448A | - | R |
| Q889Q3 | Pseudomonas syringae pv. tomato | - | R |
| Q48JS1 | Pseudomonas syringae pv. phaseolicola 1448A | - | R |
| Q48FX8 | Pseudomonas syringae pv. phaseolicola 1448A | - | R |
| Q87XG5 | Pseudomonas syringae pv. tomato | - | R |
| Q887Q7 | Pseudomonas syringae pv. tomato | - | R |
| Q4ZZ15 | Pseudomonas syringae pv. syringae B728a | - | R |
| Q48EC9 | Pseudomonas syringae pv. phaseolicola 1448A | - | R |
| Q4ZVM6 | Pseudomonas syringae pv. syringae B728a | - | R |
| Q48L09 | Pseudomonas syringae pv. phaseolicola 1448A | - | R |
| P59572 | Pseudomonas syringae pv. tomato | - | R |
| Q889E3 | Pseudomonas syringae pv. tomato | - | R |
| Q48KA5 | Pseudomonas syringae pv. phaseolicola 1448A | - | R |
| Q4ZMQ0 | Pseudomonas syringae pv. syringae B728a | - | R |
| Q48CK1 | Pseudomonas syringae pv. phaseolicola 1448A | - | R |
| **Excluded group: Chlamydia, Escherichia, Salmonella, Yersinia (animal pathogen set)** | | | |
| tir | Escherichia coli | + | E |
| espA | Escherichia coli | + | E |
| espD | Escherichia coli O157:H7 | + | E |
| tccP | Escherichia coli O157:H7 | + | E |
| st47 | Escherichia coli | + | E |
| sipA | Salmonella typhimurium | + | E |
| sspH2 | Salmonella typhimurium | + | E |
| sifA | Salmonella typhimurium | + | E |
| sopA | Salmonella typhimurium | + | E |
| sopB | Salmonella choleraesuis | + | E |
| sopE | Salmonella typhimurium | + | E |
| yopE | Yersinia pestis CA88-4125 | + | E |
| yopJ | Yersinia pestis biovar Antiqua str. B42003004 | + | E |
| yopM | Yersinia pestis CA88-4125 | + | E |
| yopT | Yersinia pseudotuberculosis | + | E |
| ypkA | Yersinia pseudotuberculosis | + | E |
| yscH | Yersinia pestis bv. Antiqua (strain Angola) | + | E |
| tarP | Chlamydophila caviae | + | E |
| tarP | Chlamydia trachomatis | + | E |
| incA | Chlamydophila caviae | + | E |
| incA | Chlamydia trachomatis (strain A/HAR-13 / ATCC VR-571B) | + | E |
| incB | Chlamydia trachomatis | + | E |
| Q9Z9F5 | Chlamydophila pneumoniae | + | E |
| Q9Z7W9 | Chlamydophila pneumoniae | + | E |
| incA | Chlamydophila pneumoniae | + | E |
| espF | Escherichia coli O157:H7 | - | E |
| EspG | Escherichia coli | - | E |
| NleB | Escherichia coli O157:H7 str. EC4196 | - | E |
| spaN | Salmonella typhimurium | - | E |
| sopD | Salmonella typhimurium | - | E |
| sptP | Salmonella typhimurium | - | E |
| yopH | Yersinia enterocolitica | - | E |
| yopK | Yersinia pseudotuberculosis | - | E |
| yopM | Yersinia pseudotuberculosis | - | E |
| copN | Chlamydophila caviae | - | E |
| IncC | Chlamydophila caviae | - | E |
| incD | Chlamydia trachomatis | - | E |
| incE | Chlamydia trachomatis | - | E |
| P78279 | Escherichia coli K-12 | + | R |
| Q2M7J9 | Escherichia coli K-12 | + | R |
| A7ZW83 | Escherichia coli HS | + | R |
| P26155 | Salmonella typhi | + | R |
| A1JS52 | Yersinia enterocolitica subsp. enterocolitica 8081 | + | R |
| Q0WCE9 | Yersinia pestis | + | R |
| A7FNN8 | Yersinia pseudotuberculosis IP 31758 | + | R |
| Q0WH55 | Yersinia pestis | + | R |
| Q93KR4 | Yersinia enterocolitica subsp. enterocolitica 8081 | + | R |
| O84859 | Chlamydia trachomatis | + | R |
| Q9JQE1 | Chlamydophila pneumoniae | + | R |
| Q9Z9F6 | Chlamydophila pneumoniae | + | R |
| Q9Z5Q4 | Chlamydophila pneumoniae | + | R |
| Q9JQI0 | Chlamydophila pneumoniae | + | R |
| Q9Z6M5 | Chlamydophila pneumoniae | + | R |
| Q3KL78 | Chlamydia trachomatis A/HAR-13 | + | R |
| Q5L593 | Chlamydophila abortus | + | R |
| Q5L6M8 | Chlamydophila abortus | + | R |
| B1IY66 | Escherichia coli ATCC 8739 | - | R |
| P78264 | Escherichia coli O6 | - | R |
| Q7ABY5 | Escherichia coli O157:H7 | - | R |
| A8A7Q7 | Escherichia coli HS | - | R |
| A7ZL97 | Escherichia coli E24377A | - | R |
| A7ZVR5 | Escherichia coli E24377A | - | R |
| A8A5C6 | Escherichia coli HS | - | R |
| P78231 | Escherichia coli O157:H7 | - | R |
| Q8FAX1 | Escherichia coli O6 | - | R |
| Q2M7N9 | Escherichia coli K-12 | - | R |
| Q2MA14 | Escherichia coli K-12 | - | R |
| Q2M7G4 | Escherichia coli K-12 | - | R |
| B1LN92 | Escherichia coli SMS-3-5 | - | R |
| Q8Z9D3 | Salmonella typhi | - | R |
| Q56120 | Salmonella typhimurium | - | R |
| Q8Z116 | Salmonella typhi | - | R |
| Q5PJE8 | Salmonella enterica subsp. enterica serovar Paratyphi A | - | R |
| Q5PDA1 | Salmonella enterica subsp. enterica serovar Paratyphi A | - | R |
| Q9CL31 | Yersinia pestis | - | R |
| A9MNX0 | Salmonella enterica subsp. arizonae serovar 62:z4,z23:-- | - | R |
| A9MHF5 | Salmonella enterica subsp. arizonae serovar 62:z4,z23:-- | - | R |
| Q9L6P6 | Salmonella typhi | - | R |
| Q9L4I4 | Salmonella typhimurium | - | R |
| Q7C7H4 | Salmonella typhi | - | R |
| Q5PLD3 | Salmonella enterica subsp. enterica serovar Paratyphi A | - | R |
| Q5PK71 | Salmonella enterica subsp. enterica serovar Paratyphi A | - | R |
| A9MPF3 | Salmonella enterica subsp. arizonae serovar 62:z4,z23:-- | - | R |
| Q5PJG1 | Salmonella enterica subsp. enterica serovar Paratyphi A | - | R |
| Q8Z9L4 | Salmonella typhi | - | R |
| Q8Z3A4 | Salmonella typhi | - | R |
| Q0WJV3 | Yersinia pestis | - | R |
| Q1C3K9 | Yersinia pestis Antiqua | - | R |
| Q667Q0 | Yersinia pseudotuberculosis | - | R |
| Q667Y4 | Yersinia pseudotuberculosis | - | R |
| Q1CNM8 | Yersinia pestis Nepal516 | - | R |
| Q0WJM6 | Yersinia pestis | - | R |
| A1JIH6 | Yersinia enterocolitica subsp. enterocolitica 8081 | - | R |
| Q1C133 | Yersinia pestis Antiqua | - | R |
| A7FH41 | Yersinia pseudotuberculosis IP 31758 | - | R |
| Q1CCU3 | Yersinia pestis Nepal516 | - | R |
| Q8D055 | Yersinia pestis | - | R |
| Q664V8 | Yersinia pseudotuberculosis | - | R |
| Q1C8B0 | Yersinia pestis Antiqua | - | R |
| O84340 | Chlamydia trachomatis | - | R |
| Q3KMC6 | Chlamydia trachomatis A/HAR-13 | - | R |
| Q823K0 | Chlamydophila caviae | - | R |
| O84402 | Chlamydia trachomatis | - | R |
| Q822Y3 | Chlamydophila caviae | - | R |
| Q5L681 | Chlamydophila abortus | - | R |
| Q9Z913 | Chlamydophila pneumoniae | - | R |
| Q255I6 | Chlamydophila felis Fe/C-56 | - | R |
| Q9K2D4 | Chlamydophila pneumoniae | - | R |
| Q3KLB6 | Chlamydia trachomatis A/HAR-13 | - | R |
| O84835 | Chlamydia trachomatis | - | R |
| Q3KKY9 | Chlamydia trachomatis A/HAR-13 | - | R |
| Q9JQ87 | Chlamydophila pneumoniae | - | R |
| Q7DET0 | Chlamydophila pneumoniae | - | R |
| Q9Z8L9 | Chlamydophila pneumoniae | - | R |
| Q3KLV9 | Chlamydia trachomatis A/HAR-13 | - | R |
| Q252X3 | Chlamydophila felis Fe/C-56 | - | R |
| Q821V0 | Chlamydophila caviae | - | R |
| Q255M0 | Chlamydophila felis Fe/C-56 | - | R |
| Q252M1 | Chlamydophila felis Fe/C-56 | - | R |
| Q9Z7P3 | Chlamydophila pneumoniae | - | R |
| Q824A6 | Chlamydophila caviae | - | R |
| O84860 | Chlamydia trachomatis | - | R |
