# Supplementary material for: Sequence-Based Prediction of Type III Secreted Proteins
Source: PLoS Pathog. 2009 Apr 24;5(4):e1000376. doi: 10.1371/journal.ppat.1000376 (PMC2669295; doi:10.1371/journal.ppat.1000376)
Supplement: Table S9 — Pair wise comparison of orthologous effector and non-effector proteins. Truncations, elongations and conservations of the N-terminal length until the first functional domain are listed according to the effector protein (first column) compared to orthologs from non-TTSS bearing organisms. (0.05 MB DOC) [file ppat.1000376.s012.doc]

Table S9. Pair wise comparison of orthologous effector and non-effector proteins

Truncations, elongations and conservations of the N-terminal length until the first functional domain are listed according to the effector protein (first column) compared to orthologs from non-TTSS bearing organisms.

| **Accession** | **Organism** | **Name** | **Elongations of effectors** | | **Truncations of effectors** | | **Equal length** | |
| --- | --- | --- | --- | --- | --- | --- | --- | --- |
| Q87XS5 | Pseudomonas syringae pv. tomato | HopAK1 | Q21F26_SACD2Q21M75_SACD2 Q21M74_SACD2 | 75% | Q21DU3_SACD2 | 25% |  | 0% |
| Q889A9 | Pseudomonas syringae pv. tomato | HopAJ1 | Q5E449_VIBF1 Q3J7V9_NITOC Q3ICF8_PSEHT | 10% | Q65U52_MANSM Q606A8_METCA Q1QD08_PSYCK Q12MA3_SHEDO | 14% | Q6FA58_ACIAD Q6F6N1_ACIAD Q9CNB9_PASMU Q9PBG0_XYLFA Q7MMK8_VIBVY Q8DFS6_VIBVU Q87C60_XYLFT Q21FD5_SACD2 Q4FQA8_PSYA2 Q6LT84_PHOPR Q31IC9_THICR Q5QWP7_IDILO Q5QYD6_IDILO Q1Q8I0_PSYCK Q1QZ47_CHRSD Q1QXA8_CHRSD Q47YD0_COLP3 Q486F6_COLP3 Q4FTV8_PSYA2 Q12QY5_SHEDO Q12NR1_SHEDO AAO54387 | 75% |
| Q05608 | Yersinia pseudotuberculosis | YpkA | gi|28057198|gb|AAO29063.1| Q7MQF4_VIBVY Q7MDP0_VIBVY Q8DDB0_VIBVU Q8D6S9_VIBVU Q8D4Y2_VIBVU Q7VM92_HAEDU Q21G42_SACD2 Q21KJ0_SACD2 Q21HY5_SACD2 Q6FAX3_ACIAD YP_089156 Q6LUE7_PHOPR Q5E1V5_VIBF1 Q5E661_VIBF1 Q12RU5_SHEDO Q487L7_COLP3 Q4FRH1_PSYA2 | 50% | Q21DN0_SACD2 Q21LQ1_SACD2 Q21GU7_SACD2 Q21IE0_SACD2 Q6LP74_PHOPR Q6LQ62_PHOPR Q60B87_METCA Q5QY67_IDILO Q5DZ80_VIBF1 Q3IL57_PSEHT Q1QB51_PSYCK Q1QA08_PSYCK Q12HU0_SHEDO Q1QV78_CHRSD Q47UD5_COLP3 Q47XY2_COLP3 Q48AJ6_COLP3 Q12NP0_SHEDO | 50% |  | 0% |
| Q93RN4 | Yersinia pseudotuberculosis | YopT | Q87AN6_XYLFT Q879T4_XYLFT Q879S8_XYLFT | 11% | Q6FDL5_ACIAD Q9CPI1_PASMU Q9CPH9_PASMU Q9PEY9_XYLFA Q9PBE8_XYLFA Q9P9U8_XYLFA Q9ZHL0_HAEDU Q87CS7_XYLFT Q87C53_XYLFT Q87AN3_XYLFT Q87AN1_XYLFT Q879S6_XYLFT Q7VLT6_HAEDU Q7VLE8_HAEDU Q4FQS9_PSYA2 Q6FDM3_ACIAD Q6F8T8_ACIAD Q65TD6_MANSM Q65TD9_MANSM Q605Q2_METCA Q1QSR3_CHRSD Q21PQ9_SACD2 | 81% | Q9PBF2_XYLFA Q65TE0_MANSM | 7% |
| Q9Z7W9 | Chlamydophila pneumoniae |  | gi_59713616 | 12% | NP_245546 gi|37199230|dbj|BAC95062.1| NP_761003 NP_873974 YP_088276 Q6LPL5_PHOPR Q5E0E4_VIBF1 | 87% |  | 0% |
| Q9RPH0 | Salmonella typhimurium | SspH2 |  | 0% | Q7MF19_VIBVY Q8D3K7_VIBVU | 100% |  | 0% |
| A6M3U5 | Yersinia pestis CA88-4125 | YopM | Q6LKB3_PHOPR Q6LQ62_PHOPR Q3IL57_PSEHT Q1QC84_PSYCK Q1QC76_PSYCK Q48AJ6_COLP3 Q12NP0_SHEDO | 58% | Q7MF19_VIBVY Q8D3K7_VIBVU Q1Q867_PSYCK Q47XC6_COLP3 Q482I4_COLP3 | 41% |  | 0% |
| B0HNP1 | Yersinia pestis biovar Antiqua str. B42003004 | YopO | gi|28057198|gb|AAO29063.1| Q7MQF4_VIBVY Q7MDP0_VIBVY Q8DDB0_VIBVU Q8D6S9_VIBVU Q8D4Y2_VIBVU Q7VM92_HAEDU Q21G42_SACD2 Q21KJ0_SACD2 Q21HY5_SACD2 Q6FAX3_ACIAD YP_089156 Q6LUE7_PHOPR Q5E1V5_VIBF1 Q5E661_VIBF1 Q12RU5_SHEDO Q487L7_COLP3 Q4FRH1_PSYA2 | 50% | Q21DN0_SACD2 Q21LQ1_SACD2 Q21GU7_SACD2 Q21IE0_SACD2 Q6LP74_PHOPR Q6LQ62_PHOPR Q60B87_METCA Q5QY67_IDILO Q5DZ80_VIBF1 Q3IL57_PSEHT Q1QB51_PSYCK Q1QA08_PSYCK Q12HU0_SHEDO Q1QV78_CHRSD Q47UD5_COLP3 Q47XY2_COLP3 Q48AJ6_COLP3 Q12NP0_SHEDO | 50% |  | 0% |
| B0HNS6 | Yersinia pestis biovar Antiqua str. B42003004 | YopT | Q87AN6_XYLFT Q879T4_XYLFT Q879S8_XYLFT | 11% | Q6FDL5_ACIAD Q9CPI1_PASMU Q9CPH9_PASMU Q9PEY9_XYLFA Q9PBE8_XYLFA Q9P9U8_XYLFA Q9ZHL0_HAEDU Q87CS7_XYLFT Q87C53_XYLFT Q87AN3_XYLFT Q87AN1_XYLFT Q879S6_XYLFT Q7VLT6_HAEDU Q7VLE8_HAEDU Q4FQS9_PSYA2 Q6FDM3_ACIAD Q6F8T8_ACIAD Q65TD6_MANSM Q65TD9_MANSM Q605Q2_METCA Q1QSR3_CHRSD Q21PQ9_SACD2 | 81% | Q9PBF2_XYLFA Q65TE0_MANSM | 7% |
| Q87V79 | Pseudomonas syringae pv. tomato | HopAN1 |  | 0% | Q9CL77_PASMU Q65R23_MANSM Q65VM1_MANSM Q65WI8_MANSM Q1QSJ7_CHRSD Q1QSN5_CHRSD | 100% |  | 0% |
| O84462 | Chlamydia trachomatis | TARP |  | 0% | Q21N17_SACD2 | 100% |  | 0% |
| Q663L9 | Yersinia pseudotuberculosis | YopM | Q1QC76_PSYCK | 16% | Q7MF19_VIBVY Q8D3K7_VIBVU Q47XC6_COLP3 Q482I4_COLP3 | 66% | Q1Q867_PSYCK | 16% |
| **Sum** | | | **57** | **30%** | **110** | **57%** | **26** | **13%** |
