# Supplementary material for: Sequence-Based Prediction of Type III Secreted Proteins
Source: PLoS Pathog. 2009 Apr 24;5(4):e1000376. doi: 10.1371/journal.ppat.1000376 (PMC2669295; doi:10.1371/journal.ppat.1000376)
Supplement: Table S10 — Effector sequences which tolerate frame shift mutations. The mutations were introduced by either shifting the DNA sequences by one or two bases to the left, stop codons where replaced by Methionine. (0.04 MB DOC) [file ppat.1000376.s013.doc]

Table S10. Effector sequences which tolerate frame shift mutations

The mutations were introduced by either shifting the DNA sequences by one or two bases to the left, stop codons where replaced by Methionine.

| **Accession** | **Organism** | **Name** | **Tolerated Mutation** |
| --- | --- | --- | --- |
| Q9Z8P7 | Chlamydophila pneumoniae | IncB | +1,+2 |
| Q9Z8P6 | Chlamydophila pneumoniae | IncC | +1 |
| Q3KMQ0 | Chlamydia trachomatis (strain A/HAR-13 / ATCC VR-571B) | IncA | +2 |
| Q9Z7Y1 | Chlamydophila pneumoniae | TARP | +2 |
| A9ZFE7 | Yersinia pestis biovar Orientalis str. IP275 | YpkA | +2 |
| Q05608 | Yersinia pseudotuberculosis | YpkA | +2 |
| Q88A09 | Pseudomonas syringae pv. tomato | HopH1 | +1,+2 |
| Q888Y7 | Pseudomonas syringae pv. tomato | HopQ1-1 | +1,+2 |
| Q9JP32 | Pseudomonas syringae pv. tomato | HopN1 | +1 |
| Q48BE0 | Pseudomonas syringae pv. phaseolicola (strain 1448A / Race 6) | HopD1 | +1,+2 |
| Q48B61 | Pseudomonas syringae pv. tomato | HopAB1 | +1 |
