# Supplementary material for: Sequence-Based Prediction of Type III Secreted Proteins
Source: PLoS Pathog. 2009 Apr 24;5(4):e1000376. doi: 10.1371/journal.ppat.1000376 (PMC2669295; doi:10.1371/journal.ppat.1000376)
Supplement: Table S11 — EffectiveT3 predictions in complete proteomes. EffectiveT3 predictions for complete proteomes have been grouped by Archaea, Gram-positive and Gram-negative bacteria. Within each group, proteomes are sorted by their taxonomic lineage and species names. For each proteome, the absence (−) or presence (+) of a TTSS, the genomic G+C content, the number of annotated proteins, the percentage of EffectiveT3 positive predictions and the genome-wide Z-Score are given. The presence of the TTSS in the proteomes as determined by KEGG and the hosts are coded by the following colors: black = without TTSS or unknown host; red = with TTSS/animal pathogenic; green = with TTSS/plant symbiotic. (0.98 MB DOC) [file ppat.1000376.s014.doc]

Table S11. EffectiveT3 predictions in complete proteomes

EffectiveT3 predictions for complete proteomes have been grouped by Archaea, Gram-positive and Gram-negative bacteria. Within each group, proteomes are sorted by their taxonomic lineage and species names. For each proteome, the absence (-) or presence (+) of a TTSS, the genomic G+C content, the number of annotated proteins, the percentage of EffectiveT3 positive predictions and the genome-wide Z-Score are given. The presence of the TTSS in the proteomes as determined by KEGG and the hosts are coded by the following colors: black=without TTSS or unknown host; red=with TTSS/animal pathogenic; green=with TTSS/plant symbiotic.

| Archaea | Lineage | TTSS status | G+C content | Number of proteins | Positives | Z-Score |
| --- | --- | --- | --- | --- | --- | --- |
| Aeropyrum pernix K1 | Crenarchaeota | - | 56.3% | 1841 | 3.7% | 0.6 |
| Caldivirga maquilingensis IC-167 | Crenarchaeota | - | 43.1% | 1963 | 1.6% | -2.3 |
| Hyperthermus butylicus DSM 5456 | Crenarchaeota | - | 53.7% | 1602 | 0.4% | -1.7 |
| Ignicoccus hospitalis KIN4/I | Crenarchaeota | - | 56.5% | 1434 | 0.2% | -2.4 |
| Metallosphaera sedula DSM 5348 | Crenarchaeota | - | 46.2% | 2256 | 1.2% | -4.1 |
| Nitrosopumilus maritimus SCM1 | Crenarchaeota | - | 34.2% | 1795 | 1.4% | -2.3 |
| Pyrobaculum aerophilum str. IM2 | Crenarchaeota | - | 51.4% | 2598 | 0.5% | -3.4 |
| Pyrobaculum arsenaticum DSM 13514 | Crenarchaeota | - | 55.1% | 2299 | 0.6% | -1.1 |
| Pyrobaculum calidifontis JCM 11548 | Crenarchaeota | - | 57.2% | 2149 | 0.7% | -0.8 |
| Pyrobaculum islandicum DSM 4184 | Crenarchaeota | - | 49.6% | 1978 | 0.7% | -1.2 |
| Staphylothermus marinus F1 | Crenarchaeota | - | 35.7% | 1570 | 0.4% | -3.8 |
| Sulfolobus acidocaldarius DSM 639 | Crenarchaeota | - | 36.7% | 2223 | 0.9% | -4.8 |
| Sulfolobus solfataricus P2 | Crenarchaeota | - | 35.8% | 2977 | 1.8% | -0.6 |
| Sulfolobus tokodaii str. 7 | Crenarchaeota | - | 32.8% | 2825 | 1.6% | -3.6 |
| Thermofilum pendens Hrk 5 | Crenarchaeota | - | 57.6% | 1876 | 0.3% | -2.6 |
| Thermoproteus neutrophilus V24Sta | Crenarchaeota | - | 59.9% | 1966 | 0.4% | -1.7 |
| Archaeoglobus fulgidus DSM 4304 | Euryarchaeota | - | 48.6% | 2419 | 0.5% | -0.7 |
| Candidatus Methanoregula boonei 6A8 | Euryarchaeota | - | 54.5% | 2450 | 1.8% | -7.2 |
| Haloarcula marismortui ATCC 43049 | Euryarchaeota | - | 61.1% | 3940 | 2.9% | 3.6 |
| Halobacterium salinarum R1 | Euryarchaeota | - | 65.7% | 2748 | 2.1% | 1.1 |
| Halobacterium sp. NRC-1 | Euryarchaeota | - | 65.9% | 2622 | 3.2% | 4.9 |
| Haloquadratum walsbyi DSM 16790 | Euryarchaeota | - | 47.9% | 2640 | 4.8% | 3.2 |
| Methanobrevibacter smithii ATCC 35061 | Euryarchaeota | - | 31.0% | 1793 | 0.9% | -4.5 |
| Methanocaldococcus jannaschii DSM 2661 | Euryarchaeota | - | 31.3% | 1785 | 0.3% | -2.3 |
| Methanococcoides burtonii DSM 6242 | Euryarchaeota | - | 40.8% | 2273 | 1.1% | -2.5 |
| Methanococcus aeolicus Nankai-3 | Euryarchaeota | - | 30.0% | 1490 | 2.2% | 1.4 |
| Methanococcus maripaludis C5 | Euryarchaeota | - | 33.0% | 1822 | 0.7% | -2.0 |
| Methanococcus maripaludis C6 | Euryarchaeota | - | 33.4% | 1826 | 0.6% | -2.5 |
| Methanococcus maripaludis C7 | Euryarchaeota | - | 33.3% | 1788 | 0.4% | -3.0 |
| Methanococcus maripaludis S2 | Euryarchaeota | - | 33.1% | 1722 | 0.6% | -2.3 |
| Methanococcus vannielii SB | Euryarchaeota | - | 31.3% | 1678 | 0.7% | -2.4 |
| Methanocorpusculum labreanum Z | Euryarchaeota | - | 50.0% | 1739 | 1.7% | -0.1 |
| Methanoculleus marisnigri JR1 | Euryarchaeota | - | 62.1% | 2489 | 1.0% | -2.0 |
| Methanopyrus kandleri AV19 | Euryarchaeota | - | 61.2% | 1687 | 0.7% | 0.3 |
| Methanosaeta thermophila PT | Euryarchaeota | - | 53.5% | 1696 | 0.6% | -2.5 |
| Methanosarcina acetivorans C2A | Euryarchaeota | - | 42.7% | 4540 | 1.5% | -2.5 |
| Methanosarcina barkeri str. Fusaro | Euryarchaeota | - | 39.2% | 3624 | 0.9% | -6.9 |
| Methanosarcina mazei Go1 | Euryarchaeota | - | 41.5% | 3368 | 1.2% | -2.8 |
| Methanosphaera stadtmanae DSM 3091 | Euryarchaeota | - | 27.6% | 1534 | 1.5% | -5.8 |
| Methanospirillum hungatei JF-1 | Euryarchaeota | - | 45.1% | 3139 | 2.1% | -1.3 |
| Methanothermobacter thermautotrophicus str. Delta H | Euryarchaeota | - | 49.5% | 1873 | 0.7% | -3.0 |
| Natronomonas pharaonis DSM 2160 | Euryarchaeota | - | 63.1% | 2822 | 1.5% | 2.8 |
| Picrophilus torridus DSM 9790 | Euryarchaeota | - | 36.0% | 1534 | 1.2% | -2.0 |
| Pyrococcus abyssi GE5 | Euryarchaeota | - | 44.7% | 1897 | 0.4% | -1.4 |
| Pyrococcus furiosus DSM 3638 | Euryarchaeota | - | 40.8% | 2124 | 0.3% | -2.6 |
| Pyrococcus horikoshii OT3 | Euryarchaeota | - | 41.9% | 1955 | 1.0% | -1.6 |
| Thermococcus kodakarensis KOD1 | Euryarchaeota | - | 52.0% | 2306 | 0.2% | -3.6 |
| Thermococcus onnurineus NA1 | Euryarchaeota | - | 51.3% | 1976 | 0.3% | -2.6 |
| Thermoplasma acidophilum DSM 1728 | Euryarchaeota | - | 46.0% | 1481 | 1.2% | -2.4 |
| Thermoplasma volcanium GSS1 | Euryarchaeota | - | 39.9% | 1499 | 0.8% | -2.9 |
| uncultured methanogenic archaeon RC-I | Euryarchaeota | - | 54.6% | 3085 | 1.6% | -5.4 |
| Nanoarchaeum equitans Kin4-M | Nanoarchaeota | - | 31.6% | 536 | 0.0% | -2.4 |
| Gram-positive bacteria | Lineage | TTSS status | G+C content | Number of proteins | Positives | Z-Score |
| Acidobacteria bacterium Ellin345 | Acidobacteria | - | 58.4% | 4777 | 3.3% | -1.0 |
| Acidothermus cellulolyticus 11B | Actinobacteria | - | 66.9% | 2157 | 3.6% | 1.1 |
| Arthrobacter aurescens TC1 | Actinobacteria | - | 62.4% | 4587 | 7.7% | 18.9 |
| Arthrobacter sp. FB24 | Actinobacteria | - | 65.4% | 4506 | 7.2% | 20.7 |
| Bifidobacterium adolescentis ATCC 15703 | Actinobacteria | - | 59.2% | 1631 | 6.1% | 6.9 |
| Bifidobacterium longum DJO10A | Actinobacteria | - | 60.2% | 1998 | 6.2% | 8.8 |
| Bifidobacterium longum NCC2705 | Actinobacteria | - | 60.1% | 1728 | 6.4% | 6.2 |
| Bifidobacterium longum subsp. infantis ATCC 15697 | Actinobacteria | - | 59.9% | 2416 | 5.3% | 7.3 |
| Clavibacter michiganensis subsp. michiganensis NCPPB 382 | Actinobacteria | - | 72.5% | 3079 | 5.0% | 12.6 |
| Clavibacter michiganensis subsp. sepedonicus | Actinobacteria | - | 72.4% | 2940 | 4.0% | 8.0 |
| Corynebacterium diphtheriae NCTC 13129 | Actinobacteria | - | 53.5% | 2272 | 5.8% | 8.5 |
| Corynebacterium efficiens YS-314 | Actinobacteria | - | 63.1% | 2950 | 9.3% | 19.4 |
| Corynebacterium glutamicum ATCC 13032 | Actinobacteria | - | 53.8% | 3056 | 5.9% | 9.5 |
| Corynebacterium glutamicum ATCC 13032 | Actinobacteria | - | 53.8% | 2993 | 6.1% | 10.0 |
| Corynebacterium glutamicum R | Actinobacteria | - | 54.1% | 3076 | 6.4% | 11.9 |
| Corynebacterium jeikeium K411 | Actinobacteria | - | 61.4% | 2119 | 10.3% | 21.1 |
| Corynebacterium urealyticum DSM 7109 | Actinobacteria | - | 64.2% | 2020 | 10.5% | 21.9 |
| Frankia alni ACN14a | Actinobacteria | - | 72.8% | 6684 | 6.0% | 11.3 |
| Frankia sp. CcI3 | Actinobacteria | - | 70.1% | 4499 | 7.5% | 15.7 |
| Frankia sp. EAN1pec | Actinobacteria | - | 71.2% | 10571 | 6.5% | 15.0 |
| Kineococcus radiotolerans SRS30216 | Actinobacteria | - | 74.2% | 4681 | 8.5% | 26.5 |
| Kocuria rhizophila DC2201 | Actinobacteria | - | 71.2% | 2357 | 9.1% | 22.6 |
| Mycobacterium abscessus | Actinobacteria | - | 64.1% | 4941 | 3.9% | 4.0 |
| Mycobacterium avium 104 | Actinobacteria | - | 69.0% | 5120 | 3.9% | 4.5 |
| Mycobacterium avium subsp. paratuberculosis K-10 | Actinobacteria | - | 69.3% | 4350 | 4.6% | 6.8 |
| Mycobacterium bovis AF2122/97 | Actinobacteria | - | 65.6% | 3920 | 4.1% | 2.6 |
| Mycobacterium bovis BCG str. Pasteur 1173P2 | Actinobacteria | - | 65.6% | 3951 | 4.0% | 2.3 |
| Mycobacterium gilvum PYR-GCK | Actinobacteria | - | 67.7% | 5579 | 4.3% | 7.4 |
| Mycobacterium leprae TN | Actinobacteria | - | 57.8% | 1605 | 4.9% | 3.4 |
| Mycobacterium marinum M | Actinobacteria | - | 65.7% | 5452 | 4.3% | 1.8 |
| Mycobacterium smegmatis str. MC2 155 | Actinobacteria | - | 67.4% | 6715 | 4.6% | 10.5 |
| Mycobacterium sp. JLS | Actinobacteria | - | 68.4% | 5739 | 4.3% | 10.7 |
| Mycobacterium sp. KMS | Actinobacteria | - | 68.2% | 5975 | 4.3% | 9.3 |
| Mycobacterium sp. MCS | Actinobacteria | - | 68.4% | 5615 | 4.4% | 10.4 |
| Mycobacterium tuberculosis CDC1551 | Actinobacteria | - | 65.6% | 4189 | 5.2% | 5.9 |
| Mycobacterium tuberculosis F11 | Actinobacteria | - | 65.6% | 3941 | 4.4% | 3.6 |
| Mycobacterium tuberculosis H37Ra | Actinobacteria | - | 65.3% | 4034 | 3.9% | 2.2 |
| Mycobacterium tuberculosis H37Rv | Actinobacteria | - | 65.6% | 3989 | 4.0% | 2.5 |
| Mycobacterium ulcerans Agy99 | Actinobacteria | - | 65.5% | 4160 | 4.2% | 4.5 |
| Mycobacterium vanbaalenii PYR-1 | Actinobacteria | - | 67.8% | 5979 | 3.6% | 5.5 |
| Nocardia farcinica IFM 10152 | Actinobacteria | - | 70.7% | 5936 | 4.7% | 11.8 |
| Nocardioides sp. JS614 | Actinobacteria | - | 71.4% | 4909 | 3.9% | 11.5 |
| Propionibacterium acnes KPA171202 | Actinobacteria | - | 60.0% | 2297 | 7.2% | 10.5 |
| Renibacterium salmoninarum ATCC 33209 | Actinobacteria | - | 56.3% | 3506 | 6.4% | 10.0 |
| Rhodococcus jostii RHA1 | Actinobacteria | - | 67.0% | 9144 | 6.0% | 18.4 |
| Rubrobacter xylanophilus DSM 9941 | Actinobacteria | - | 70.5% | 3140 | 0.8% | 0.8 |
| Saccharopolyspora erythraea NRRL 2338 | Actinobacteria | - | 71.2% | 7197 | 3.6% | 9.8 |
| Salinispora arenicola CNS-205 | Actinobacteria | - | 69.5% | 4917 | 4.4% | 8.0 |
| Salinispora tropica CNB-440 | Actinobacteria | - | 69.5% | 4536 | 4.9% | 9.8 |
| Streptomyces avermitilis MA-4680 | Actinobacteria | - | 70.7% | 7673 | 6.7% | 19.4 |
| Streptomyces coelicolor A3(2) | Actinobacteria | - | 72.0% | 8152 | 6.5% | 23.4 |
| Streptomyces griseus subsp. griseus NBRC 13350 | Actinobacteria | - | 72.2% | 7136 | 7.5% | 31.5 |
| Thermobifida fusca YX | Actinobacteria | - | 67.5% | 3110 | 5.9% | 13.2 |
| Tropheryma whipplei str. Twist | Actinobacteria | - | 46.3% | 808 | 2.8% | -0.6 |
| Tropheryma whipplei TW08/27 | Actinobacteria | - | 46.3% | 783 | 2.3% | -1.6 |
| Bacteroides vulgatus ATCC 8482 | Bacteroidetes/Chlorobi | - | 42.2% | 4065 | 1.1% | -4.1 |
| Candidatus Azobacteroides pseudotrichonymphae genomovar. CFP2 | Bacteroidetes/Chlorobi | - | 32.9% | 852 | 1.3% | -0.7 |
| Candidatus Sulcia muelleri GWSS | Bacteroidetes/Chlorobi | - | 22.4% | 227 | 1.8% | 0.0 |
| Chlorobaculum parvum NCIB 8327 | Bacteroidetes/Chlorobi | - | 55.8% | 2043 | 2.5% | 4.0 |
| Chlorobium tepidum TLS | Bacteroidetes/Chlorobi | - | 56.5% | 2252 | 2.5% | 3.2 |
| Parabacteroides distasonis ATCC 8503 | Bacteroidetes/Chlorobi | - | 45.1% | 3850 | 1.2% | -3.7 |
| Chloroflexus aurantiacus J-10-fl | Chloroflexi | - | 56.7% | 3853 | 3.5% | 2.5 |
| Dehalococcoides ethenogenes 195 | Chloroflexi | - | 48.9% | 1580 | 1.5% | -0.9 |
| Dehalococcoides sp. BAV1 | Chloroflexi | - | 47.2% | 1371 | 1.5% | -1.1 |
| Dehalococcoides sp. CBDB1 | Chloroflexi | - | 47.0% | 1458 | 1.4% | -1.2 |
| Roseiflexus castenholzii DSM 13941 | Chloroflexi | - | 60.7% | 4330 | 3.1% | 3.0 |
| Acaryochloris marina MBIC11017 | Cyanobacteria | - | 47.0% | 8383 | 6.6% | 10.9 |
| Anabaena variabilis ATCC 29413 | Cyanobacteria | - | 41.4% | 5661 | 6.9% | 10.8 |
| Cyanothece sp. ATCC 51142 | Cyanobacteria | - | 37.9% | 5298 | 6.2% | 10.9 |
| Cyanothece sp. PCC 7424 | Cyanobacteria | - | 38.5% | 5710 | 6.5% | 12.7 |
| Cyanothece sp. PCC 8801 | Cyanobacteria | - | 39.8% | 4367 | 6.2% | 7.7 |
| Gloeobacter violaceus PCC 7421 | Cyanobacteria | - | 62.0% | 4430 | 3.2% | 6.0 |
| Microcystis aeruginosa NIES-843 | Cyanobacteria | - | 42.3% | 6312 | 5.3% | 8.4 |
| Nostoc punctiforme PCC 73102 | Cyanobacteria | - | 41.4% | 6689 | 6.3% | 8.9 |
| Nostoc sp. PCC 7120 | Cyanobacteria | - | 41.3% | 6123 | 6.5% | 9.2 |
| Prochlorococcus marinus str. MIT 9301 | Cyanobacteria | - | 31.3% | 1907 | 2.7% | 2.3 |
| Prochlorococcus marinus str. MIT 9313 | Cyanobacteria | - | 50.7% | 2269 | 6.6% | 11.3 |
| Prochlorococcus marinus str. MIT 9515 | Cyanobacteria | - | 30.8% | 1905 | 3.5% | 4.5 |
| Prochlorococcus marinus subsp. marinus str. CCMP1375 | Cyanobacteria | - | 36.4% | 1883 | 5.2% | 7.6 |
| Prochlorococcus marinus subsp. pastoris str. CCMP1986 | Cyanobacteria | - | 30.8% | 1717 | 3.0% | 2.3 |
| Synechococcus elongatus PCC 7942 | Cyanobacteria | - | 55.4% | 2662 | 4.1% | 3.5 |
| Synechococcus sp. CC9311 | Cyanobacteria | - | 52.4% | 2892 | 5.5% | 9.3 |
| Synechococcus sp. CC9605 | Cyanobacteria | - | 59.2% | 2645 | 5.1% | 9.4 |
| Synechococcus sp. CC9902 | Cyanobacteria | - | 54.2% | 2306 | 5.1% | 8.7 |
| Synechococcus sp. PCC 7002 | Cyanobacteria | - | 49.2% | 3186 | 5.6% | 7.7 |
| Synechococcus sp. RCC307 | Cyanobacteria | - | 60.8% | 2533 | 4.5% | 6.3 |
| Synechococcus sp. WH 7803 | Cyanobacteria | - | 60.2% | 2533 | 3.8% | 4.9 |
| Synechococcus sp. WH 8102 | Cyanobacteria | - | 59.4% | 2519 | 5.1% | 8.8 |
| Synechocystis sp. PCC 6803 | Cyanobacteria | - | 47.4% | 3527 | 5.1% | 7.2 |
| Thermosynechococcus elongatus BP-1 | Cyanobacteria | - | 53.9% | 2476 | 5.0% | 7.3 |
| Trichodesmium erythraeum IMS101 | Cyanobacteria | - | 34.1% | 4451 | 5.6% | 9.0 |
| Deinococcus geothermalis DSM 11300 | Deinococcus-Thermus | - | 66.5% | 2857 | 5.0% | 11.1 |
| Deinococcus radiodurans R1 | Deinococcus-Thermus | - | 66.6% | 3181 | 6.8% | 12.4 |
| Thermus thermophilus HB27 | Deinococcus-Thermus | - | 69.4% | 1982 | 0.5% | -0.4 |
| Thermus thermophilus HB8 | Deinococcus-Thermus | - | 69.5% | 2235 | 0.5% | -1.5 |
| Acholeplasma laidlawii PG-8A | Firmicutes | - | 31.9% | 1379 | 1.1% | -1.3 |
| Alkaliphilus metalliredigens QYMF | Firmicutes | - | 36.8% | 4625 | 1.4% | -1.3 |
| Alkaliphilus oremlandii OhILAs | Firmicutes | - | 36.3% | 2836 | 1.0% | -2.0 |
| Anoxybacillus flavithermus WK1 | Firmicutes | - | 41.8% | 2830 | 1.4% | 0.1 |
| Aster yellows witches'-broom phytoplasma AYWB | Firmicutes | - | 26.8% | 693 | 1.9% | -2.2 |
| Bacillus amyloliquefaciens FZB42 | Firmicutes | - | 46.5% | 3693 | 1.5% | -1.4 |
| Bacillus anthracis str. Ames | Firmicutes | - | 35.4% | 5311 | 2.0% | -0.2 |
| Bacillus anthracis str. 'Ames Ancestor' | Firmicutes |  | 35.2% | 5617 | 2.0% | -0.1 |
| Bacillus anthracis str. Sterne | Firmicutes | - | 35.4% | 5287 | 2.1% | 0.3 |
| Bacillus cereus AH187 | Firmicutes | - | 35.5% | 5758 | 2.0% | -1.3 |
| Bacillus cereus ATCC 10987 | Firmicutes | - | 35.5% | 5844 | 1.9% | -1.0 |
| Bacillus cereus ATCC 14579 | Firmicutes | - | 35.3% | 5254 | 2.0% | -1.6 |
| Bacillus cereus B4264 | Firmicutes | - | 35.3% | 5408 | 2.0% | -1.3 |
| Bacillus cereus E33L | Firmicutes | - | 35.1% | 5641 | 2.0% | -0.9 |
| Bacillus cereus subsp. cytotoxis NVH 391-98 | Firmicutes | - | 35.9% | 3844 | 1.7% | -1.5 |
| Bacillus clausii KSM-K16 | Firmicutes | - | 44.8% | 4096 | 1.6% | 1.1 |
| Bacillus halodurans C-125 | Firmicutes | - | 43.7% | 4044 | 1.6% | 0.7 |
| Bacillus licheniformis ATCC 14580 | Firmicutes | - | 46.2% | 4152 | 1.4% | -1.1 |
| Bacillus licheniformis ATCC 14580 | Firmicutes | - | 46.2% | 4195 | 1.4% | -1.5 |
| Bacillus pumilus SAFR-032 | Firmicutes | - | 41.3% | 3678 | 1.6% | -1.8 |
| Bacillus subtilis subsp. subtilis str. 168 | Firmicutes | - | 43.5% | 4100 | 1.8% | -0.3 |
| Bacillus thuringiensis serovar konkukian str. 97-27 | Firmicutes | - | 35.4% | 5197 | 1.9% | -1.8 |
| Bacillus thuringiensis str. Al Hakam | Firmicutes | - | 35.4% | 4798 | 1.7% | -2.5 |
| Bacillus weihenstephanensis KBAB4 | Firmicutes | - | 35.5% | 5653 | 2.1% | -1.2 |
| Candidatus Desulforudis audaxviator MP104C | Firmicutes | - | 60.8% | 2157 | 0.6% | -0.6 |
| Candidatus Phytoplasma australiense | Firmicutes | - | 27.4% | 684 | 3.7% | 1.8 |
| Carboxydothermus hydrogenoformans Z-2901 | Firmicutes | - | 42.0% | 2620 | 0.3% | -3.6 |
| Clostridium acetobutylicum ATCC 824 | Firmicutes | - | 30.9% | 3848 | 1.3% | -5.3 |
| Clostridium beijerinckii NCIMB 8052 | Firmicutes | - | 29.9% | 5020 | 1.5% | -4.7 |
| Clostridium botulinum A str. ATCC 19397 | Firmicutes | - | 28.2% | 3551 | 1.1% | -3.3 |
| Clostridium botulinum A str. ATCC 3502 | Firmicutes | - | 28.2% | 1565 | 1.2% | -1.6 |
| Clostridium botulinum A str. Hall | Firmicutes | - | 28.2% | 3403 | 1.1% | -2.4 |
| Clostridium botulinum A3 str. Loch Maree | Firmicutes | - | 28.1% | 3984 | 1.4% | -2.3 |
| Clostridium botulinum B str. Eklund 17B | Firmicutes | - | 27.5% | 3527 | 1.4% | -2.8 |
| Clostridium botulinum B1 str. Okra | Firmicutes | - | 28.2% | 3852 | 1.2% | -1.9 |
| Clostridium botulinum E3 str. Alaska E43 | Firmicutes | - | 27.4% | 3256 | 1.4% | -3.2 |
| Clostridium botulinum F str. Langeland | Firmicutes | - | 28.3% | 3659 | 1.1% | -3.0 |
| Clostridium difficile 630 | Firmicutes | - | 29.1% | 3750 | 1.1% | -2.8 |
| Clostridium kluyveri DSM 555 | Firmicutes | - | 32.0% | 6732 | 1.7% | -2.8 |
| Clostridium novyi NT | Firmicutes | - | 28.9% | 2325 | 1.2% | -1.6 |
| Clostridium perfringens ATCC 13124 | Firmicutes | - | 28.4% | 2876 | 0.8% | -3.0 |
| Clostridium perfringens SM101 | Firmicutes | - | 28.2% | 2578 | 0.8% | -2.5 |
| Clostridium perfringens str. 13 | Firmicutes | - | 28.5% | 2723 | 0.6% | -3.4 |
| Clostridium phytofermentans ISDg | Firmicutes | - | 35.3% | 3902 | 1.6% | -3.0 |
| Clostridium tetani E88 | Firmicutes | - | 28.6% | 2432 | 0.8% | -3.4 |
| Clostridium thermocellum ATCC 27405 | Firmicutes | - | 39.0% | 3189 | 0.8% | -4.1 |
| Desulfotomaculum reducens MI-1 | Firmicutes | - | 42.3% | 4065 | 1.3% | -1.9 |
| Enterococcus faecalis V583 | Firmicutes | - | 37.4% | 3248 | 1.4% | -3.8 |
| Exiguobacterium sibiricum 255-15 | Firmicutes | - | 47.7% | 3015 | 1.5% | 0.1 |
| Finegoldia magna ATCC 29328 | Firmicutes | - | 32.1% | 1812 | 0.6% | -2.9 |
| Geobacillus kaustophilus HTA426 | Firmicutes | - | 52.0% | 3539 | 1.2% | -0.3 |
| Geobacillus thermodenitrificans NG80-2 | Firmicutes | - | 48.9% | 3445 | 1.7% | 1.7 |
| Lactobacillus acidophilus NCFM | Firmicutes | - | 34.7% | 1864 | 1.4% | -4.3 |
| Lactobacillus brevis ATCC 367 | Firmicutes | - | 46.1% | 2214 | 2.7% | -3.6 |
| Lactobacillus casei ATCC 334 | Firmicutes | - | 46.6% | 2758 | 2.5% | -1.0 |
| Lactobacillus delbrueckii subsp. bulgaricus ATCC 11842 | Firmicutes | - | 49.7% | 1556 | 1.2% | -2.7 |
| Lactobacillus delbrueckii subsp. bulgaricus ATCC BAA-365 | Firmicutes | - | 49.7% | 1704 | 1.3% | -2.5 |
| Lactobacillus fermentum IFO 3956 | Firmicutes | - | 51.5% | 1843 | 2.9% | -0.3 |
| Lactobacillus gasseri ATCC 33323 | Firmicutes | - | 35.3% | 1755 | 1.5% | -3.1 |
| Lactobacillus helveticus DPC 4571 | Firmicutes | - | 37.1% | 1610 | 1.6% | -2.1 |
| Lactobacillus johnsonii NCC 533 | Firmicutes | - | 34.6% | 1821 | 1.9% | -2.6 |
| Lactobacillus plantarum WCFS1 | Firmicutes | - | 44.4% | 3058 | 3.5% | -3.4 |
| Lactobacillus reuteri F275 | Firmicutes | - | 38.9% | 1900 | 1.8% | -3.5 |
| Lactobacillus reuteri F275 | Firmicutes | - | 38.9% | 1820 | 1.8% | -3.5 |
| Lactobacillus sakei subsp. sakei 23K | Firmicutes | - | 41.3% | 1867 | 2.6% | -2.4 |
| Lactobacillus salivarius UCC118 | Firmicutes | - | 33.0% | 2017 | 0.9% | -4.9 |
| Lactococcus lactis subsp. cremoris MG1363 | Firmicutes | - | 35.7% | 2433 | 1.5% | -4.3 |
| Lactococcus lactis subsp. cremoris SK11 | Firmicutes | - | 35.8% | 2498 | 1.2% | -5.5 |
| Lactococcus lactis subsp. lactis Il1403 | Firmicutes | - | 35.3% | 2321 | 1.9% | -4.0 |
| Leuconostoc citreum KM20 | Firmicutes | - | 38.9% | 1820 | 3.0% | -0.9 |
| Leuconostoc mesenteroides subsp. mesenteroides ATCC 8293 | Firmicutes | - | 37.7% | 2001 | 1.9% | -4.8 |
| Listeria innocua Clip11262 | Firmicutes | - | 37.4% | 2968 | 1.3% | -2.3 |
| Listeria monocytogenes EGD-e | Firmicutes | - | 38.0% | 2846 | 1.1% | -3.5 |
| Listeria monocytogenes HCC23 | Firmicutes | - | 38.2% | 2974 | 1.3% | -1.8 |
| Listeria monocytogenes str. 4b F2365 | Firmicutes | - | 38.0% | 2821 | 1.2% | -2.8 |
| Listeria welshimeri serovar 6b str. SLCC5334 | Firmicutes | - | 36.4% | 2774 | 1.2% | -2.2 |
| Lysinibacillus sphaericus C3-41 | Firmicutes | - | 37.1% | 4771 | 2.1% | -0.5 |
| Moorella thermoacetica ATCC 39073 | Firmicutes | - | 55.8% | 2465 | 0.9% | -1.9 |
| Natranaerobius thermophilus JW/NM-WN-LF | Firmicutes | - | 36.3% | 2906 | 2.0% | 2.9 |
| Oceanobacillus iheyensis HTE831 | Firmicutes | - | 35.7% | 3500 | 2.6% | 2.2 |
| Oenococcus oeni PSU-1 | Firmicutes | - | 37.9% | 1687 | 1.7% | -2.6 |
| Onion yellows phytoplasma OY-M | Firmicutes | - | 27.7% | 754 | 2.4% | -2.3 |
| Pediococcus pentosaceus ATCC 25745 | Firmicutes | - | 37.4% | 1748 | 1.7% | -3.6 |
| Pelotomaculum thermopropionicum SI | Firmicutes | - | 53.0% | 2920 | 0.9% | -0.6 |
| Staphylococcus aureus RF122 | Firmicutes | - | 32.8% | 2515 | 2.1% | -1.6 |
| Staphylococcus aureus subsp. aureus COL | Firmicutes | - | 32.8% | 2617 | 2.0% | -2.5 |
| Staphylococcus aureus subsp. aureus JH1 | Firmicutes | - | 32.9% | 2780 | 1.8% | -3.6 |
| Staphylococcus aureus subsp. aureus JH9 | Firmicutes | - | 32.9% | 2726 | 1.8% | -3.1 |
| Staphylococcus aureus subsp. aureus MRSA252 | Firmicutes | - | 32.8% | 2653 | 2.0% | -2.1 |
| Staphylococcus aureus subsp. aureus MSSA476 | Firmicutes | - | 32.8% | 2594 | 1.9% | -2.8 |
| Staphylococcus aureus subsp. aureus Mu3 | Firmicutes | - | 32.9% | 2695 | 1.9% | -2.6 |
| Staphylococcus aureus subsp. aureus Mu50 | Firmicutes | - | 32.8% | 829 | 1.6% | -1.5 |
| Staphylococcus aureus subsp. aureus MW2 | Firmicutes | - | 32.8% | 2629 | 1.7% | -3.0 |
| Staphylococcus aureus subsp. aureus N315 | Firmicutes | - | 32.8% | 2052 | 1.7% | -2.2 |
| Staphylococcus aureus subsp. aureus NCTC 8325 | Firmicutes | - | 32.9% | 2892 | 1.9% | -3.2 |
| Staphylococcus aureus subsp. aureus str. Newman | Firmicutes | - | 32.9% | 2611 | 1.7% | -3.6 |
| Staphylococcus aureus subsp. aureus USA300 | Firmicutes | - | 32.7% | 2560 | 2.0% | -2.3 |
| Staphylococcus aureus subsp. aureus USA300_TCH1516 | Firmicutes | - | 32.7% | 2678 | 1.9% | -2.6 |
| Staphylococcus epidermidis ATCC 12228 | Firmicutes | - | 32.0% | 2484 | 1.7% | -3.2 |
| Staphylococcus epidermidis RP62A | Firmicutes | - | 32.1% | 2520 | 1.7% | -3.4 |
| Staphylococcus haemolyticus JCSC1435 | Firmicutes | - | 32.8% | 2658 | 1.8% | -2.3 |
| Staphylococcus saprophyticus subsp. saprophyticus ATCC 15305 | Firmicutes | - | 33.2% | 2509 | 1.7% | -2.0 |
| Streptococcus agalactiae 2603V/R | Firmicutes | - | 35.6% | 2123 | 1.4% | -3.2 |
| Streptococcus agalactiae A909 | Firmicutes | - | 35.6% | 1996 | 1.7% | -1.9 |
| Streptococcus agalactiae NEM316 | Firmicutes | - | 35.6% | 2094 | 1.4% | -3.3 |
| Streptococcus equi subsp. zooepidemicus MGCS10565 | Firmicutes | - | 41.8% | 1893 | 0.8% | -3.7 |
| Streptococcus gordonii str. Challis substr. CH1 | Firmicutes | - | 40.5% | 2051 | 1.7% | -1.8 |
| Streptococcus mutans UA159 | Firmicutes | - | 36.8% | 1959 | 1.1% | -3.4 |
| Streptococcus pneumoniae CGSP14 | Firmicutes | - | 39.5% | 2206 | 1.1% | -2.5 |
| Streptococcus pneumoniae D39 | Firmicutes | - | 39.7% | 1914 | 1.2% | -1.9 |
| Streptococcus pneumoniae G54 | Firmicutes | - | 39.6% | 2115 | 1.2% | -2.5 |
| Streptococcus pneumoniae Hungary19A-6 | Firmicutes | - | 39.6% | 2155 | 1.5% | -1.8 |
| Streptococcus pneumoniae R6 | Firmicutes | - | 39.7% | 2042 | 1.3% | -2.1 |
| Streptococcus pneumoniae TIGR4 | Firmicutes | - | 39.6% | 2105 | 1.2% | -2.3 |
| Streptococcus pyogenes M1 GAS | Firmicutes | - | 38.5% | 1697 | 0.8% | -3.5 |
| Streptococcus pyogenes MGAS10270 | Firmicutes | - | 38.4% | 1987 | 1.3% | -2.7 |
| Streptococcus pyogenes MGAS10394 | Firmicutes | - | 38.7% | 1886 | 1.2% | -2.6 |
| Streptococcus pyogenes MGAS10750 | Firmicutes | - | 38.3% | 1979 | 1.1% | -2.8 |
| Streptococcus pyogenes MGAS2096 | Firmicutes | - | 38.7% | 1898 | 1.6% | -1.4 |
| Streptococcus pyogenes MGAS315 | Firmicutes | - | 38.6% | 1865 | 1.4% | -1.7 |
| Streptococcus pyogenes MGAS5005 | Firmicutes | - | 38.5% | 1865 | 0.9% | -3.4 |
| Streptococcus pyogenes MGAS6180 | Firmicutes | - | 38.4% | 1894 | 1.1% | -3.1 |
| Streptococcus pyogenes MGAS8232 | Firmicutes | - | 38.5% | 1845 | 0.9% | -3.5 |
| Streptococcus pyogenes MGAS9429 | Firmicutes | - | 38.5% | 1876 | 1.3% | -1.9 |
| Streptococcus pyogenes NZ131 | Firmicutes | - | 38.6% | 1700 | 1.1% | -2.5 |
| Streptococcus pyogenes SSI-1 | Firmicutes | - | 38.6% | 1861 | 1.2% | -2.4 |
| Streptococcus pyogenes str. Manfredo | Firmicutes | - | 38.6% | 1745 | 0.9% | -3.2 |
| Streptococcus sanguinis SK36 | Firmicutes | - | 43.4% | 2270 | 1.3% | -3.3 |
| Streptococcus suis 05ZYH33 | Firmicutes | - | 41.1% | 2186 | 1.5% | -1.4 |
| Streptococcus suis 98HAH33 | Firmicutes | - | 41.1% | 2185 | 1.6% | -1.3 |
| Streptococcus thermophilus CNRZ1066 | Firmicutes | - | 39.1% | 1913 | 1.7% | -1.4 |
| Streptococcus thermophilus LMD-9 | Firmicutes | - | 39.1% | 1704 | 1.8% | -1.1 |
| Streptococcus thermophilus LMG 18311 | Firmicutes | - | 39.1% | 1889 | 1.8% | -0.8 |
| Symbiobacterium thermophilum IAM 14863 | Firmicutes | - | 68.7% | 3337 | 2.5% | 5.4 |
| Thermoanaerobacter pseudethanolicus ATCC 33223 | Firmicutes | - | 34.5% | 2243 | 0.5% | -3.2 |
| Thermoanaerobacter sp. X514 | Firmicutes | - | 34.5% | 2349 | 0.4% | -3.4 |
| Ureaplasma parvum serovar 3 str. ATCC 27815 | Firmicutes | - | 25.5% | 609 | 3.0% | 1.1 |
| Ureaplasma parvum serovar 3 str. ATCC 700970 | Firmicutes | - | 25.5% | 614 | 2.4% | 0.0 |
| Caldicellulosiruptor saccharolyticus DSM 8903 | Other Bacteria | - | 35.3% | 2679 | 0.6% | -3.1 |
| Candidatus Phytoplasma mali | Other Bacteria | - | 21.4% | 479 | 2.1% | -0.6 |
| Dictyoglomus turgidum DSM 6724 | Other Bacteria | - | 34.0% | 1744 | 0.2% | -3.3 |
| Elusimicrobium minutum Pei191 | Other Bacteria | - | 40.0% | 1529 | 0.3% | -4.7 |
| Methylacidiphilum infernorum V4 | Other Bacteria | - | 45.5% | 2472 | 1.6% | 0.7 |
| Opitutus terrae PB90-1 | Other Bacteria | - | 65.3% | 4612 | 3.8% | 8.0 |
| Ureaplasma urealyticum serovar 10 str. ATCC 33699 | Other Bacteria | - | 25.8% | 646 | 3.3% | 0.4 |
| Borrelia burgdorferi B31 | Spirochaetes | - | 28.2% | 1640 | 2.7% | 2.1 |
| Borrelia burgdorferi ZS7 | Spirochaetes | - | 28.5% | 1043 | 2.6% | 2.3 |
| Borrelia duttonii Ly | Spirochaetes | - | 28.0% | 1305 | 1.5% | -1.0 |
| Borrelia garinii PBi | Spirochaetes | - | 28.1% | 925 | 1.1% | -1.7 |
| Borrelia hermsii DAH | Spirochaetes | - | 29.8% | 819 | 0.6% | -1.5 |
| Borrelia turicatae 91E135 | Spirochaetes | - | 29.1% | 818 | 0.7% | -1.5 |
| Leptospira interrogans serovar Copenhageni str. Fiocruz L1-130 | Spirochaetes | - | 35.0% | 3658 | 1.9% | -2.2 |
| Leptospira interrogans serovar Lai str. 56601 | Spirochaetes | - | 35.0% | 4727 | 1.8% | -2.1 |
| Treponema denticola ATCC 35405 | Spirochaetes | - | 37.9% | 2767 | 1.0% | -1.4 |
| Treponema pallidum subsp. pallidum SS14 | Spirochaetes | - | 52.8% | 1027 | 1.0% | -2.2 |
| Gram-negative bacteria | Lineage | TTSS status | G+C content | Number of proteins | Positives | Z-Score |
| Solibacter usitatus Ellin6076 | Acidobacteria | - | 61.9% | 7826 | 2.2% | -5.2 |
| Leifsonia xyli subsp. xyli str. CTCB07 | Actinobacteria | - | 67.7% | 2025 | 4.1% | 4.6 |
| Acidiphilium cryptum JF-5 | Alphaproteobacteria | - | 67.1% | 3559 | 2.6% | 1.9 |
| Agrobacterium tumefaciens str. C58 | Alphaproteobacteria | - | 59.0% | 5288 | 3.5% | 10.5 |
| Anaplasma phagocytophilum HZ | Alphaproteobacteria | - | 41.6% | 1264 | 1.5% | -1.6 |
| Bartonella bacilliformis KC583 | Alphaproteobacteria | - | 38.2% | 1283 | 2.7% | 0.9 |
| Bartonella henselae str. Houston-1 | Alphaproteobacteria | - | 38.2% | 1488 | 2.8% | 0.4 |
| Bartonella quintana str. Toulouse | Alphaproteobacteria | - | 38.8% | 1142 | 2.5% | 0.7 |
| Bartonella tribocorum CIP 105476 | Alphaproteobacteria | - | 38.8% | 2092 | 3.1% | 2.7 |
| Beijerinckia indica subsp. indica ATCC 9039 | Alphaproteobacteria | - | 57.0% | 3784 | 3.0% | 3.3 |
| Bradyrhizobium japonicum USDA 110 | Alphaproteobacteria | + | 64.1% | 8317 | 3.5% | 7.1 |
| Bradyrhizobium sp. BTAi1 | Alphaproteobacteria | - | 64.8% | 7612 | 3.2% | 6.6 |
| Brucella abortus bv. 1 str. 9-941 | Alphaproteobacteria | - | 57.2% | 3085 | 2.4% | 4.0 |
| Brucella abortus S19 | Alphaproteobacteria | - | 57.2% | 3000 | 2.4% | 3.9 |
| Brucella canis ATCC 23365 | Alphaproteobacteria | - | 57.2% | 3249 | 2.4% | 3.7 |
| Brucella melitensis 16M | Alphaproteobacteria | - | 57.2% | 3198 | 2.8% | 5.9 |
| Brucella melitensis biovar Abortus 2308 | Alphaproteobacteria | - | 57.2% | 3034 | 2.3% | 3.8 |
| Brucella ovis ATCC 25840 | Alphaproteobacteria | - | 57.2% | 2888 | 2.5% | 4.6 |
| Brucella suis 1330 | Alphaproteobacteria | - | 57.3% | 3271 | 2.4% | 3.4 |
| Brucella suis ATCC 23445 | Alphaproteobacteria | - | 57.2% | 3240 | 2.5% | 4.4 |
| Candidatus Pelagibacter ubique HTCC1062 | Alphaproteobacteria | - | 29.7% | 1354 | 1.8% | -0.1 |
| Caulobacter crescentus CB15 | Alphaproteobacteria | - | 67.2% | 3737 | 2.5% | 3.9 |
| Caulobacter sp. K31 | Alphaproteobacteria | - | 67.3% | 5438 | 2.1% | 1.4 |
| Dinoroseobacter shibae DFL 12 | Alphaproteobacteria | - | 65.5% | 4187 | 2.4% | 6.8 |
| Ehrlichia canis str. Jake | Alphaproteobacteria | - | 29.0% | 925 | 4.1% | 0.3 |
| Ehrlichia ruminantium str. Gardel | Alphaproteobacteria | - | 27.5% | 950 | 2.4% | -2.7 |
| Ehrlichia ruminantium str. Welgevonden | Alphaproteobacteria | - | 27.5% | 958 | 2.3% | -3.4 |
| Ehrlichia ruminantium str. Welgevonden | Alphaproteobacteria | - | 27.5% | 888 | 2.3% | -3.0 |
| Erythrobacter litoralis HTCC2594 | Alphaproteobacteria | - | 63.1% | 3007 | 1.9% | 1.3 |
| Gluconacetobacter diazotrophicus PAl 5 | Alphaproteobacteria | - | 66.3% | 3849 | 3.7% | 4.4 |
| Gluconacetobacter diazotrophicus PAl 5 | Alphaproteobacteria | - | 66.3% | 3501 | 3.6% | 3.6 |
| Gluconobacter oxydans 621H | Alphaproteobacteria | - | 60.8% | 2664 | 5.6% | 6.2 |
| Granulibacter bethesdensis CGDNIH1 | Alphaproteobacteria | - | 59.1% | 2437 | 5.3% | 8.4 |
| Hyphomonas neptunium ATCC 15444 | Alphaproteobacteria | - | 61.9% | 3505 | 2.4% | 2.9 |
| Jannaschia sp. CCS1 | Alphaproteobacteria | - | 62.2% | 4283 | 3.1% | 7.1 |
| Magnetospirillum magneticum AMB-1 | Alphaproteobacteria | - | 65.1% | 4559 | 3.3% | 6.0 |
| Maricaulis maris MCS10 | Alphaproteobacteria | - | 62.7% | 3063 | 1.9% | 1.8 |
| Mesorhizobium loti MAFF303099 | Alphaproteobacteria | + | 62.5% | 7272 | 2.8% | 6.1 |
| Mesorhizobium sp. BNC1 | Alphaproteobacteria | + | 61.1% | 4543 | 2.3% | 5.2 |
| Methylobacterium chloromethanicum CM4 | Alphaproteobacteria | - | 68.1% | 5516 | 2.9% | 5.5 |
| Methylobacterium extorquens PA1 | Alphaproteobacteria | - | 68.2% | 4829 | 3.5% | 9.3 |
| Methylobacterium populi BJ001 | Alphaproteobacteria | - | 69.4% | 5365 | 2.8% | 5.8 |
| Methylobacterium sp. 4-46 | Alphaproteobacteria | - | 71.5% | 6692 | 2.6% | 5.8 |
| Methylocella silvestris BL2 | Alphaproteobacteria | - | 63.1% | 3818 | 1.9% | 0.1 |
| Nitrobacter hamburgensis X14 | Alphaproteobacteria | - | 61.6% | 4326 | 3.2% | 3.8 |
| Nitrobacter winogradskyi Nb-255 | Alphaproteobacteria | - | 62.0% | 3122 | 3.0% | 4.0 |
| Novosphingobium aromaticivorans DSM 12444 | Alphaproteobacteria | - | 65.1% | 3324 | 2.0% | 0.8 |
| Oligotropha carboxidovorans OM5 | Alphaproteobacteria | - | 62.4% | 3722 | 3.3% | 5.4 |
| Orientia tsutsugamushi str. Boryong | Alphaproteobacteria | - | 30.5% | 1182 | 4.5% | 1.4 |
| Orientia tsutsugamushi str. Ikeda | Alphaproteobacteria | - | 30.5% | 1967 | 3.8% | 0.5 |
| Paracoccus denitrificans PD1222 | Alphaproteobacteria | - | 66.8% | 5077 | 1.7% | 2.9 |
| Parvibaculum lavamentivorans DS-1 | Alphaproteobacteria | - | 62.3% | 3636 | 2.0% | 4.6 |
| Phenylobacterium zucineum HLK1 | Alphaproteobacteria | - | 71.1% | 3854 | 1.5% | 1.2 |
| Rhizobium leguminosarum bv. trifolii WSM2304 | Alphaproteobacteria | - | 61.2% | 6415 | 2.3% | 3.7 |
| Rhizobium leguminosarum bv. viciae 3841 | Alphaproteobacteria | - | 55.0% | 7149 | 2.9% | 8.3 |
| Rhodobacter sphaeroides 2.4.1 | Alphaproteobacteria | - | 68.8% | 4241 | 1.9% | 4.7 |
| Rhodobacter sphaeroides ATCC 17025 | Alphaproteobacteria | - | 68.2% | 4333 | 2.1% | 5.8 |
| Rhodobacter sphaeroides ATCC 17029 | Alphaproteobacteria | - | 69.0% | 4132 | 1.8% | 4.9 |
| Rhodopseudomonas palustris BisA53 | Alphaproteobacteria | - | 64.4% | 4878 | 3.4% | 5.7 |
| Rhodopseudomonas palustris BisB18 | Alphaproteobacteria | - | 65.0% | 4886 | 3.2% | 5.0 |
| Rhodopseudomonas palustris BisB5 | Alphaproteobacteria | - | 64.8% | 4397 | 2.9% | 4.1 |
| Rhodopseudomonas palustris CGA009 | Alphaproteobacteria | - | 65.0% | 4812 | 3.4% | 5.0 |
| Rhodopseudomonas palustris HaA2 | Alphaproteobacteria | - | 66.0% | 4683 | 2.9% | 4.1 |
| Rhodopseudomonas palustris TIE-1 | Alphaproteobacteria | - | 64.9% | 5246 | 2.9% | 3.6 |
| Rhodospirillum centenum SW | Alphaproteobacteria | - | 70.5% | 4002 | 3.9% | 8.6 |
| Rhodospirillum rubrum ATCC 11170 | Alphaproteobacteria | - | 65.4% | 3840 | 4.1% | 13.3 |
| Rickettsia akari str. Hartford | Alphaproteobacteria | - | 32.3% | 1250 | 3.5% | 1.1 |
| Rickettsia bellii RML369-C | Alphaproteobacteria | - | 31.6% | 1429 | 2.0% | -1.7 |
| Rickettsia canadensis str. McKiel | Alphaproteobacteria | - | 31.1% | 1084 | 2.8% | -0.3 |
| Rickettsia conorii str. Malish 7 | Alphaproteobacteria | - | 32.4% | 1374 | 3.1% | 0.2 |
| Rickettsia felis URRWXCal2 | Alphaproteobacteria | - | 32.5% | 1512 | 3.4% | 1.5 |
| Rickettsia massiliae MTU5 | Alphaproteobacteria | - | 32.5% | 980 | 3.2% | 1.6 |
| Rickettsia prowazekii str. Madrid E | Alphaproteobacteria | - | 29.0% | 835 | 2.0% | -0.9 |
| Rickettsia rickettsii str. Iowa | Alphaproteobacteria | - | 32.4% | 1380 | 2.5% | -1.2 |
| Rickettsia rickettsii str. 'Sheila Smith' | Alphaproteobacteria | - | 32.5% | 1337 | 2.9% | 0.1 |
| Rickettsia typhi str. Wilmington | Alphaproteobacteria | - | 28.9% | 837 | 2.2% | -0.8 |
| Roseobacter denitrificans OCh 114 | Alphaproteobacteria | - | 58.9% | 4129 | 2.2% | 4.1 |
| Silicibacter pomeroyi DSS-3 | Alphaproteobacteria | - | 64.1% | 4252 | 2.0% | 5.0 |
| Silicibacter sp. TM1040 | Alphaproteobacteria | - | 60.1% | 3864 | 2.2% | 4.0 |
| Sinorhizobium medicae WSM419 | Alphaproteobacteria | - | 61.1% | 6213 | 2.1% | 4.4 |
| Sinorhizobium meliloti 1021 | Alphaproteobacteria | - | 62.2% | 6205 | 1.9% | 3.6 |
| Sphingomonas wittichii RW1 | Alphaproteobacteria | - | 67.9% | 5345 | 1.6% | 1.3 |
| Sphingopyxis alaskensis RB2256 | Alphaproteobacteria | - | 65.5% | 3195 | 2.2% | 4.3 |
| Wolbachia endosymbiont of Drosophila melanogaster | Alphaproteobacteria | - | 35.2% | 1195 | 2.8% | 0.8 |
| Wolbachia endosymbiont strain TRS of Brugia malayi | Alphaproteobacteria | - | 34.2% | 805 | 2.0% | -0.3 |
| Wolbachia pipientis | Alphaproteobacteria | - | 34.9% | 1275 | 2.9% | 3.2 |
| Xanthobacter autotrophicus Py2 | Alphaproteobacteria | - | 67.3% | 5035 | 3.5% | 10.1 |
| Zymomonas mobilis subsp. mobilis ZM4 | Alphaproteobacteria | - | 46.3% | 1998 | 3.6% | 2.3 |
| Aquifex aeolicus VF5 | Aquificae | - | 43.3% | 1560 | 0.3% | -0.5 |
| Hydrogenobaculum sp. Y04AAS1 | Aquificae | - | 34.8% | 1629 | 1.2% | -3.4 |
| Sulfurihydrogenibium sp. YO3AOP1 | Aquificae | - | 32.0% | 1721 | 0.5% | -2.5 |
| Bacteroides fragilis NCTC 9343 | Bacteroidetes/Chlorobi | - | 43.1% | 4231 | 1.0% | -4.6 |
| Bacteroides fragilis YCH46 | Bacteroidetes/Chlorobi | - | 43.2% | 4625 | 1.1% | -4.8 |
| Bacteroides thetaiotaomicron VPI-5482 | Bacteroidetes/Chlorobi | - | 42.9% | 4816 | 1.3% | -4.9 |
| Chlorobium chlorochromatii CaD3 | Bacteroidetes/Chlorobi | - | 44.3% | 2002 | 3.9% | 4.7 |
| Chlorobium limicola DSM 245 | Bacteroidetes/Chlorobi | - | 51.3% | 2434 | 1.8% | 0.5 |
| Chlorobium phaeobacteroides BS1 | Bacteroidetes/Chlorobi | - | 48.9% | 2469 | 1.9% | 2.3 |
| Chlorobium phaeobacteroides DSM 266 | Bacteroidetes/Chlorobi | - | 48.4% | 2650 | 2.2% | 1.3 |
| Chloroherpeton thalassium ATCC 35110 | Bacteroidetes/Chlorobi | - | 45.0% | 2710 | 2.1% | 2.0 |
| Cytophaga hutchinsonii ATCC 33406 | Bacteroidetes/Chlorobi | - | 38.8% | 3785 | 2.3% | -4.6 |
| Flavobacterium johnsoniae UW101 | Bacteroidetes/Chlorobi | - | 34.1% | 5017 | 1.6% | -5.7 |
| Flavobacterium psychrophilum JIP02/86 | Bacteroidetes/Chlorobi | - | 32.5% | 2412 | 2.2% | -3.3 |
| Gramella forsetii KT0803 | Bacteroidetes/Chlorobi | - | 36.6% | 3584 | 0.7% | -4.6 |
| Pelodictyon luteolum DSM 273 | Bacteroidetes/Chlorobi | - | 57.3% | 2083 | 2.7% | 5.1 |
| Pelodictyon phaeoclathratiforme BU-1 | Bacteroidetes/Chlorobi | - | 48.1% | 2707 | 2.1% | 2.1 |
| Porphyromonas gingivalis ATCC 33277 | Bacteroidetes/Chlorobi | - | 48.4% | 2089 | 0.8% | -2.4 |
| Porphyromonas gingivalis W83 | Bacteroidetes/Chlorobi | - | 48.3% | 1909 | 1.3% | -0.7 |
| Prosthecochloris aestuarii DSM 271 | Bacteroidetes/Chlorobi | - | 50.1% | 2327 | 2.7% | 3.2 |
| Salinibacter ruber DSM 13855 | Bacteroidetes/Chlorobi | - | 66.1% | 2833 | 7.1% | 16.8 |
| Acidovorax avenae subsp. citrulli AAC00-1 | Betaproteobacteria | + | 68.5% | 4709 | 7.5% | 23.4 |
| Acidovorax sp. JS42 | Betaproteobacteria | - | 66.1% | 4154 | 5.6% | 15.8 |
| Aromatoleum aromaticum EbN1 | Betaproteobacteria | - | 64.7% | 4596 | 2.9% | 4.0 |
| Azoarcus sp. BH72 | Betaproteobacteria | - | 67.9% | 3989 | 2.7% | 9.4 |
| Bordetella avium 197N | Betaproteobacteria | - | 61.6% | 3380 | 3.1% | 5.3 |
| Bordetella bronchiseptica RB50 | Betaproteobacteria | + | 68.1% | 4994 | 2.8% | 8.8 |
| Bordetella parapertussis 12822 | Betaproteobacteria | + | 68.1% | 4185 | 2.8% | 7.4 |
| Bordetella pertussis Tohama I | Betaproteobacteria | + | 67.7% | 3436 | 2.2% | 4.5 |
| Bordetella petrii DSM 12804 | Betaproteobacteria | - | 65.5% | 5027 | 3.4% | 9.8 |
| Burkholderia ambifaria MC40-6 | Betaproteobacteria | + | 66.4% | 6697 | 3.4% | 6.0 |
| Burkholderia cenocepacia J2315 | Betaproteobacteria | + | 66.9% | 7114 | 3.2% | 7.0 |
| Burkholderia cenocepacia MC0-3 | Betaproteobacteria | + | 66.6% | 7008 | 3.3% | 8.4 |
| Burkholderia mallei ATCC 23344 | Betaproteobacteria | + | 68.5% | 5024 | 2.9% | 4.2 |
| Burkholderia mallei NCTC 10229 | Betaproteobacteria | + | 68.5% | 5509 | 2.9% | 4.1 |
| Burkholderia mallei NCTC 10247 | Betaproteobacteria | + | 68.5% | 5852 | 2.9% | 4.6 |
| Burkholderia mallei SAVP1 | Betaproteobacteria | - | 68.4% | 5189 | 2.9% | 4.3 |
| Burkholderia multivorans ATCC 17616 | Betaproteobacteria | + | 66.7% | 6259 | 3.5% | 9.8 |
| Burkholderia multivorans ATCC 17616 | Betaproteobacteria | + | 66.7% | 6111 | 3.1% | 7.0 |
| Burkholderia phymatum STM815 | Betaproteobacteria | - | 62.3% | 7496 | 4.4% | 11.2 |
| Burkholderia phytofirmans PsJN | Betaproteobacteria | + | 62.3% | 7241 | 4.8% | 11.4 |
| Burkholderia pseudomallei 1106a | Betaproteobacteria | + | 68.3% | 7183 | 2.8% | 3.0 |
| Burkholderia pseudomallei 1710b | Betaproteobacteria | + | 68.0% | 6347 | 3.5% | 6.1 |
| Burkholderia pseudomallei 668 | Betaproteobacteria | + | 68.3% | 7230 | 3.0% | 4.0 |
| Burkholderia pseudomallei K96243 | Betaproteobacteria | + | 68.1% | 5728 | 3.1% | 5.6 |
| Burkholderia sp. 383 | Betaproteobacteria | - | 66.3% | 7717 | 3.8% | 10.0 |
| Burkholderia thailandensis E264 | Betaproteobacteria | + | 67.6% | 5634 | 3.5% | 7.3 |
| Burkholderia vietnamiensis G4 | Betaproteobacteria | + | 65.7% | 7617 | 3.7% | 9.8 |
| Chromobacterium violaceum ATCC 12472 | Betaproteobacteria | + | 64.8% | 4407 | 2.7% | 1.5 |
| Dechloromonas aromatica RCB | Betaproteobacteria | - | 59.2% | 4171 | 3.0% | 5.9 |
| Delftia acidovorans SPH-1 | Betaproteobacteria | - | 66.5% | 6040 | 7.4% | 23.2 |
| Methylibium petroleiphilum PM1 | Betaproteobacteria | - | 68.8% | 4449 | 4.0% | 13.3 |
| Methylobacillus flagellatus KT | Betaproteobacteria | - | 55.7% | 2753 | 2.9% | 2.6 |
| Neisseria gonorrhoeae FA 1090 | Betaproteobacteria | - | 52.7% | 2002 | 2.2% | 2.4 |
| Neisseria gonorrhoeae NCCP11945 | Betaproteobacteria | - | 52.4% | 2674 | 2.3% | 1.5 |
| Neisseria meningitidis 053442 | Betaproteobacteria | - | 51.7% | 2020 | 2.5% | 3.1 |
| Neisseria meningitidis FAM18 | Betaproteobacteria | - | 51.6% | 1917 | 2.5% | 3.0 |
| Neisseria meningitidis MC58 | Betaproteobacteria | - | 51.5% | 2062 | 2.1% | 0.4 |
| Neisseria meningitidis Z2491 | Betaproteobacteria | - | 51.8% | 2062 | 1.9% | 0.7 |
| Nitrosomonas europaea ATCC 19718 | Betaproteobacteria | - | 50.7% | 2461 | 3.0% | 3.6 |
| Nitrosomonas eutropha C91 | Betaproteobacteria | - | 48.5% | 2550 | 3.2% | 3.7 |
| Nitrosospira multiformis ATCC 25196 | Betaproteobacteria | - | 53.9% | 2805 | 3.1% | 3.2 |
| Polaromonas naphthalenivorans CJ2 | Betaproteobacteria | - | 61.7% | 4929 | 5.5% | 15.5 |
| Polaromonas sp. JS666 | Betaproteobacteria | - | 62.0% | 5453 | 6.0% | 22.0 |
| Polynucleobacter necessarius subsp. asymbioticus QLW-P1DMWA-1 | Betaproteobacteria | - | 44.8% | 2077 | 3.3% | 2.6 |
| Polynucleobacter necessarius subsp. necessarius STIR1 | Betaproteobacteria | - | 45.6% | 1508 | 3.8% | 3.1 |
| Ralstonia eutropha H16 | Betaproteobacteria | - | 66.3% | 6206 | 4.6% | 17.1 |
| Ralstonia metallidurans CH34 | Betaproteobacteria | - | 63.5% | 6319 | 4.8% | 12.7 |
| Ralstonia pickettii 12J | Betaproteobacteria | - | 63.6% | 4950 | 4.6% | 9.7 |
| Rhodoferax ferrireducens T118 | Betaproteobacteria | - | 59.6% | 4418 | 4.4% | 10.6 |
| Thauera sp. MZ1T | Betaproteobacteria | - | 68.1% | 2343 | 2.5% | 7.6 |
| Thiobacillus denitrificans ATCC 25259 | Betaproteobacteria | - | 66.1% | 2827 | 2.0% | 3.3 |
| Verminephrobacter eiseniae EF01-2 | Betaproteobacteria | - | 65.2% | 4947 | 4.5% | 12.5 |
| Candidatus Protochlamydia amoebophila UWE25 | Chlamydiae/Verrucomicrobia | + | 34.7% | 2031 | 6.1% | 12.4 |
| Chlamydia muridarum Nigg | Chlamydiae/Verrucomicrobia | + | 40.3% | 911 | 4.1% | 2.2 |
| Chlamydia trachomatis 434/Bu | Chlamydiae/Verrucomicrobia | + | 41.3% | 874 | 4.2% | 2.9 |
| Chlamydia trachomatis A/HAR-13 | Chlamydiae/Verrucomicrobia | + | 41.3% | 919 | 4.8% | 3.8 |
| Chlamydia trachomatis D/UW-3/CX | Chlamydiae/Verrucomicrobia | + | 41.3% | 895 | 4.7% | 3.7 |
| Chlamydia trachomatis L2b/UCH-1/proctitis | Chlamydiae/Verrucomicrobia | + | 41.3% | 874 | 4.3% | 3.5 |
| Chlamydophila abortus S26/3 | Chlamydiae/Verrucomicrobia | + | 39.9% | 932 | 5.8% | 5.6 |
| Chlamydophila caviae GPIC | Chlamydiae/Verrucomicrobia | + | 39.2% | 1005 | 5.6% | 5.8 |
| Chlamydophila felis Fe/C-56 | Chlamydiae/Verrucomicrobia | + | 39.3% | 1013 | 5.3% | 3.9 |
| Chlamydophila pneumoniae AR39 | Chlamydiae/Verrucomicrobia | + | 40.6% | 1112 | 4.9% | 4.0 |
| Chlamydophila pneumoniae CWL029 | Chlamydiae/Verrucomicrobia | + | 40.6% | 1052 | 5.8% | 5.7 |
| Chlamydophila pneumoniae J138 | Chlamydiae/Verrucomicrobia | + | 40.6% | 1069 | 5.7% | 5.2 |
| Chlamydophila pneumoniae TW-183 | Chlamydiae/Verrucomicrobia | + | 40.6% | 1113 | 4.9% | 3.6 |
| Herpetosiphon aurantiacus ATCC 23779 | Chloroflexi | - | 50.9% | 5278 | 4.4% | -2.8 |
| Roseiflexus sp. RS-1 | Chloroflexi | - | 60.4% | 4517 | 3.0% | 2.3 |
| Prochlorococcus marinus str. AS9601 | Cyanobacteria | - | 31.3% | 1921 | 2.8% | 2.8 |
| Prochlorococcus marinus str. MIT 9211 | Cyanobacteria | - | 38.0% | 1854 | 5.0% | 8.0 |
| Prochlorococcus marinus str. MIT 9215 | Cyanobacteria | - | 31.1% | 1982 | 2.6% | 2.3 |
| Prochlorococcus marinus str. MIT 9303 | Cyanobacteria | - | 50.0% | 2997 | 5.9% | 8.4 |
| Prochlorococcus marinus str. MIT 9312 | Cyanobacteria | - | 31.2% | 1810 | 2.9% | 2.6 |
| Prochlorococcus marinus str. NATL1A | Cyanobacteria | - | 35.0% | 2193 | 5.2% | 10.2 |
| Prochlorococcus marinus str. NATL2A | Cyanobacteria | - | 35.1% | 1892 | 5.6% | 9.4 |
| Synechococcus elongatus PCC 6301 | Cyanobacteria | - | 55.5% | 2526 | 4.3% | 4.0 |
| Synechococcus sp. JA-2-3B'a(2-13) | Cyanobacteria | - | 58.5% | 2862 | 5.5% | 8.4 |
| Synechococcus sp. JA-3-3Ab | Cyanobacteria | - | 60.2% | 2760 | 6.1% | 12.6 |
| Bdellovibrio bacteriovorus HD100 | Deltaproteobacteria | - | 50.6% | 2446 | 1.6% | -3.8 |
| Desulfatibacillum alkenivorans AK-01 | Deltaproteobacteria | - | 54.5% | 5252 | 1.4% | 0.1 |
| Desulfococcus oleovorans Hxd3 | Deltaproteobacteria | - | 56.2% | 3265 | 1.3% | 0.8 |
| Desulfotalea psychrophila LSv54 | Deltaproteobacteria | - | 46.6% | 3234 | 2.8% | 3.3 |
| Desulfovibrio desulfuricans subsp. desulfuricans str. G20 | Deltaproteobacteria | - | 57.8% | 3775 | 2.4% | 4.1 |
| Desulfovibrio vulgaris DP4 | Deltaproteobacteria | + | 63.2% | 3091 | 3.0% | 5.8 |
| Desulfovibrio vulgaris str. Hildenborough | Deltaproteobacteria | + | 63.3% | 3529 | 3.1% | 5.4 |
| Geobacter bemidjiensis Bem | Deltaproteobacteria | - | 60.3% | 4018 | 1.2% | -3.5 |
| Geobacter lovleyi SZ | Deltaproteobacteria | - | 54.7% | 3685 | 2.0% | -1.3 |
| Geobacter metallireducens GS-15 | Deltaproteobacteria | - | 59.5% | 3532 | 1.4% | -1.0 |
| Geobacter sulfurreducens PCA | Deltaproteobacteria | - | 60.9% | 3446 | 1.4% | -1.5 |
| Geobacter uraniireducens Rf4 | Deltaproteobacteria | - | 54.2% | 4357 | 1.2% | -4.7 |
| Lawsonia intracellularis PHE/MN1-00 | Deltaproteobacteria | + | 33.1% | 1185 | 4.5% | 0.8 |
| Pelobacter carbinolicus DSM 2380 | Deltaproteobacteria | - | 55.1% | 3353 | 1.5% | 0.5 |
| Pelobacter propionicus DSM 2379 | Deltaproteobacteria | - | 58.5% | 3804 | 2.4% | 1.9 |
| Sorangium cellulosum 'So ce 56' | Deltaproteobacteria | - | 71.4% | 9384 | 3.3% | 2.2 |
| Syntrophobacter fumaroxidans MPOB | Deltaproteobacteria | - | 59.9% | 4064 | 1.4% | 0.1 |
| Syntrophus aciditrophicus SB | Deltaproteobacteria | - | 51.5% | 3168 | 1.2% | -1.9 |
| Arcobacter butzleri RM4018 | Epsilonproteobacteria | - | 27.0% | 2259 | 1.9% | -1.0 |
| Campylobacter concisus 13826 | Epsilonproteobacteria | - | 39.3% | 1985 | 1.8% | 0.8 |
| Campylobacter curvus 525.92 | Epsilonproteobacteria | - | 44.5% | 1931 | 1.5% | -0.3 |
| Campylobacter fetus subsp. fetus 82-40 | Epsilonproteobacteria | - | 33.3% | 1719 | 1.7% | -1.3 |
| Campylobacter hominis ATCC BAA-381 | Epsilonproteobacteria | - | 31.7% | 1687 | 0.8% | -2.8 |
| Campylobacter jejuni RM1221 | Epsilonproteobacteria | - | 30.3% | 1838 | 1.8% | -0.1 |
| Campylobacter jejuni subsp. doylei 269.97 | Epsilonproteobacteria | - | 30.6% | 1731 | 1.8% | 0.2 |
| Campylobacter jejuni subsp. jejuni 81116 | Epsilonproteobacteria | - | 30.5% | 1626 | 1.8% | 0.2 |
| Campylobacter jejuni subsp. jejuni 81-176 | Epsilonproteobacteria | - | 30.6% | 1758 | 1.8% | -0.8 |
| Campylobacter jejuni subsp. jejuni NCTC 11168 | Epsilonproteobacteria | - | 30.5% | 1628 | 1.8% | -0.7 |
| Helicobacter acinonychis str. Sheeba | Epsilonproteobacteria | - | 38.2% | 1618 | 1.5% | -2.3 |
| Helicobacter hepaticus ATCC 51449 | Epsilonproteobacteria | - | 35.9% | 1875 | 2.8% | 1.1 |
| Helicobacter pylori 26695 | Epsilonproteobacteria | - | 38.9% | 1567 | 1.9% | -1.5 |
| Helicobacter pylori G27 | Epsilonproteobacteria | - | 38.9% | 1504 | 1.3% | -3.0 |
| Helicobacter pylori HPAG1 | Epsilonproteobacteria | - | 39.1% | 1540 | 1.8% | -1.7 |
| Helicobacter pylori J99 | Epsilonproteobacteria | - | 39.2% | 1490 | 2.1% | -0.9 |
| Helicobacter pylori P12 | Epsilonproteobacteria | - | 38.8% | 1577 | 1.5% | -3.1 |
| Helicobacter pylori Shi470 | Epsilonproteobacteria | - | 38.9% | 1553 | 1.7% | -2.0 |
| Nitratiruptor sp. SB155-2 | Epsilonproteobacteria | - | 39.7% | 1843 | 0.7% | -1.3 |
| Sulfurimonas denitrificans DSM 1251 | Epsilonproteobacteria | - | 34.5% | 2097 | 1.4% | -1.7 |
| Sulfurovum sp. NBC37-1 | Epsilonproteobacteria | - | 43.9% | 2438 | 0.9% | -1.2 |
| Wolinella succinogenes DSM 1740 | Epsilonproteobacteria | - | 48.5% | 2043 | 1.7% | 1.5 |
| Coprothermobacter proteolyticus DSM 5265 | Firmicutes | - | 44.8% | 1482 | 1.6% | 0.8 |
| Desulfitobacterium hafniense Y51 | Firmicutes | - | 47.4% | 5060 | 1.5% | -2.1 |
| Heliobacterium modesticaldum Ice1 | Firmicutes | - | 57.0% | 3000 | 1.8% | -0.1 |
| Mesoplasma florum L1 | Firmicutes | - | 27.0% | 682 | 1.3% | -2.1 |
| Mycoplasma agalactiae PG2 | Firmicutes | - | 29.7% | 742 | 1.6% | -1.5 |
| Mycoplasma arthritidis 158L3-1 | Firmicutes | - | 30.7% | 631 | 1.6% | -0.7 |
| Mycoplasma capricolum subsp. capricolum ATCC 27343 | Firmicutes | - | 23.8% | 812 | 1.4% | -4.0 |
| Mycoplasma gallisepticum R | Firmicutes | - | 31.5% | 726 | 2.8% | -3.4 |
| Mycoplasma genitalium G37 | Firmicutes | - | 31.4% | 484 | 1.9% | -1.5 |
| Mycoplasma hyopneumoniae 232 | Firmicutes | - | 28.6% | 691 | 1.3% | -1.8 |
| Mycoplasma hyopneumoniae 7448 | Firmicutes | - | 28.5% | 663 | 1.2% | -1.9 |
| Mycoplasma hyopneumoniae J | Firmicutes | - | 28.5% | 665 | 2.1% | -0.5 |
| Mycoplasma mobile 163K | Firmicutes | - | 25.0% | 633 | 2.7% | -2.1 |
| Mycoplasma mycoides subsp. mycoides SC str. PG1 | Firmicutes | - | 24.0% | 1016 | 2.2% | -2.8 |
| Mycoplasma penetrans HF-2 | Firmicutes | - | 25.7% | 1037 | 3.7% | -5.5 |
| Mycoplasma pneumoniae M129 | Firmicutes | - | 40.0% | 689 | 5.2% | 0.3 |
| Mycoplasma pulmonis UAB CTIP | Firmicutes | - | 26.6% | 782 | 2.3% | -1.9 |
| Mycoplasma synoviae 53 | Firmicutes | - | 28.5% | 672 | 2.4% | -2.2 |
| Thermoanaerobacter tengcongensis MB4 | Firmicutes | - | 37.6% | 2588 | 0.5% | -2.5 |
| Fusobacterium nucleatum subsp. nucleatum ATCC 25586 | Fusobacteria | - | 27.2% | 2063 | 0.8% | -0.9 |
| Acidithiobacillus ferrooxidans ATCC 23270 | Gammaproteobacteria | - | 58.8% | 3147 | 2.3% | -1.5 |
| Acinetobacter baumannii AB0057 | Gammaproteobacteria | - | 39.2% | 3801 | 4.0% | 3.7 |
| Acinetobacter baumannii AB307-0294 | Gammaproteobacteria | - | 39.0% | 3451 | 4.2% | 4.2 |
| Acinetobacter baumannii ACICU | Gammaproteobacteria | - | 38.9% | 3759 | 4.2% | 4.5 |
| Acinetobacter baumannii ATCC 17978 | Gammaproteobacteria | - | 38.9% | 3368 | 3.7% | 2.3 |
| Acinetobacter baumannii AYE | Gammaproteobacteria | - | 39.3% | 3711 | 3.6% | 3.0 |
| Acinetobacter baumannii SDF | Gammaproteobacteria | - | 39.1% | 2974 | 3.2% | 1.9 |
| Acinetobacter sp. ADP1 | Gammaproteobacteria | - | 40.4% | 3315 | 4.3% | 2.4 |
| Actinobacillus pleuropneumoniae L20 | Gammaproteobacteria | - | 41.3% | 2012 | 1.7% | -1.0 |
| Actinobacillus pleuropneumoniae serovar 3 str. JL03 | Gammaproteobacteria | - | 41.2% | 2035 | 1.6% | -1.4 |
| Actinobacillus pleuropneumoniae serovar 7 str. AP76 | Gammaproteobacteria | - | 41.2% | 2142 | 1.6% | -1.6 |
| Actinobacillus succinogenes 130Z | Gammaproteobacteria | - | 44.9% | 2079 | 1.4% | -1.8 |
| Aeromonas hydrophila subsp. hydrophila ATCC 7966 | Gammaproteobacteria | - | 61.5% | 4118 | 3.1% | 6.3 |
| Aeromonas salmonicida subsp. salmonicida A449 | Gammaproteobacteria | + | 58.2% | 4436 | 3.0% | 4.8 |
| Alcanivorax borkumensis SK2 | Gammaproteobacteria | - | 54.7% | 2754 | 3.3% | 5.4 |
| Aliivibrio salmonicida LFI1238 | Gammaproteobacteria | - | 39.0% | 3807 | 3.0% | 2.6 |
| Alteromonas macleodii 'Deep ecotype' | Gammaproteobacteria | - | 44.9% | 4071 | 3.4% | 3.2 |
| Buchnera aphidicola str. APS (Acyrthosiphon pisum) | Gammaproteobacteria | - | 26.4% | 564 | 1.8% | -1.0 |
| Buchnera aphidicola str. Bp (Baizongia pistaciae) | Gammaproteobacteria | - | 25.3% | 507 | 1.6% | -2.3 |
| Buchnera aphidicola str. Cc (Cinara cedri) | Gammaproteobacteria | - | 20.1% | 357 | 1.7% | -0.5 |
| Buchnera aphidicola str. Sg (Schizaphis graminum) | Gammaproteobacteria | - | 25.3% | 546 | 1.3% | -1.5 |
| Candidatus Blochmannia floridanus | Gammaproteobacteria | - | 27.4% | 583 | 2.1% | -2.2 |
| Cellvibrio japonicus Ueda107 | Gammaproteobacteria | - | 52.0% | 3754 | 4.6% | 3.4 |
| Chromohalobacter salexigens DSM 3043 | Gammaproteobacteria | - | 63.9% | 3298 | 3.1% | 9.7 |
| Citrobacter koseri ATCC BAA-895 | Gammaproteobacteria | - | 53.8% | 5026 | 2.5% | -0.9 |
| Colwellia psychrerythraea 34H | Gammaproteobacteria | - | 38.0% | 4905 | 4.5% | 5.3 |
| Coxiella burnetii CbuG_Q212 | Gammaproteobacteria | - | 42.6% | 1864 | 2.0% | -0.7 |
| Coxiella burnetii CbuK_Q154 | Gammaproteobacteria | - | 42.6% | 1939 | 1.7% | -1.6 |
| Coxiella burnetii Dugway 5J108-111 | Gammaproteobacteria | - | 42.4% | 2125 | 2.2% | -0.2 |
| Coxiella burnetii RSA 331 | Gammaproteobacteria | - | 42.7% | 1975 | 2.0% | -1.3 |
| Coxiella burnetii RSA 493 | Gammaproteobacteria | - | 42.6% | 2052 | 2.1% | -0.5 |
| Dichelobacter nodosus VCS1703A | Gammaproteobacteria | - | 44.4% | 1280 | 1.8% | 0.0 |
| Enterobacter sakazakii ATCC BAA-894 | Gammaproteobacteria | - | 56.7% | 4442 | 2.7% | 1.8 |
| Escherichia coli 536 | Gammaproteobacteria | - | 50.5% | 4628 | 2.9% | 0.8 |
| Escherichia coli APEC O1 | Gammaproteobacteria | - | 50.3% | 4467 | 2.6% | -0.6 |
| Escherichia coli CFT073 | Gammaproteobacteria | - | 50.5% | 5373 | 2.9% | 0.2 |
| Escherichia coli K-12 | Gammaproteobacteria | - | 50.8% | 4209 | 2.9% | 0.8 |
| Escherichia coli O127:H6 str. E2348/69 | Gammaproteobacteria | + | 50.5% | 4647 | 2.6% | -0.8 |
| Escherichia coli O157:H7 EDL933 | Gammaproteobacteria | + | 50.3% | 5298 | 3.4% | 2.2 |
| Escherichia coli O157:H7 str. EC4115 | Gammaproteobacteria | + | 50.4% | 5477 | 3.1% | 1.5 |
| Escherichia coli O157:H7 str. Sakai | Gammaproteobacteria | + | 50.5% | 5336 | 3.2% | 0.7 |
| Escherichia coli str. K-12 substr. DH10B | Gammaproteobacteria | - | 50.8% | 4120 | 2.7% | 0.5 |
| Escherichia coli str. K-12 substr. W3110 | Gammaproteobacteria | - | 50.8% | 4220 | 2.8% | 1.0 |
| Escherichia coli UTI89 | Gammaproteobacteria | - | 50.6% | 5208 | 2.6% | -1.2 |
| Escherichia fergusonii ATCC 35469 | Gammaproteobacteria | - | 49.9% | 4257 | 2.9% | 1.7 |
| Francisella novicida U112 | Gammaproteobacteria | - | 32.5% | 1719 | 1.7% | -3.8 |
| Francisella philomiragia subsp. philomiragia ATCC 25017 | Gammaproteobacteria | - | 32.6% | 1915 | 2.1% | -2.0 |
| Francisella tularensis subsp. holarctica FTNF002-00 | Gammaproteobacteria | - | 32.2% | 1580 | 1.5% | -4.0 |
| Francisella tularensis subsp. holarctica OSU18 | Gammaproteobacteria | - | 32.2% | 1555 | 1.9% | -3.1 |
| Francisella tularensis subsp. mediasiatica FSC147 | Gammaproteobacteria | - | 32.3% | 1406 | 1.6% | -3.5 |
| Francisella tularensis subsp. tularensis FSC198 | Gammaproteobacteria | - | 32.3% | 1605 | 2.1% | -3.0 |
| Francisella tularensis subsp. tularensis SCHU S4 | Gammaproteobacteria | - | 32.3% | 1603 | 2.1% | -3.3 |
| Francisella tularensis subsp. tularensis WY96-3418 | Gammaproteobacteria | - | 32.3% | 1634 | 2.1% | -2.4 |
| Haemophilus ducreyi 35000HP | Gammaproteobacteria | - | 38.2% | 1717 | 1.9% | -0.7 |
| Haemophilus influenzae 86-028NP | Gammaproteobacteria | - | 38.2% | 1791 | 3.1% | 2.5 |
| Haemophilus influenzae PittEE | Gammaproteobacteria | - | 38.0% | 1611 | 3.2% | 3.2 |
| Haemophilus influenzae PittGG | Gammaproteobacteria | - | 38.0% | 1656 | 2.8% | 2.2 |
| Haemophilus influenzae Rd KW20 | Gammaproteobacteria | - | 38.1% | 1657 | 2.5% | 1.6 |
| Haemophilus somnus 129PT | Gammaproteobacteria | - | 37.2% | 1798 | 2.4% | 0.8 |
| Haemophilus somnus 2336 | Gammaproteobacteria | - | 37.4% | 1980 | 2.4% | 0.1 |
| Hahella chejuensis KCTC 2396 | Gammaproteobacteria | + | 53.9% | 6778 | 2.5% | 2.4 |
| Halorhodospira halophila SL1 | Gammaproteobacteria | - | 68.0% | 2407 | 1.8% | 4.3 |
| Idiomarina loihiensis L2TR | Gammaproteobacteria | - | 47.0% | 2616 | 2.6% | 1.2 |
| Klebsiella pneumoniae 342 | Gammaproteobacteria | - | 56.9% | 5763 | 2.4% | 0.9 |
| Klebsiella pneumoniae subsp. pneumoniae MGH 78578 | Gammaproteobacteria | - | 57.1% | 5182 | 2.0% | -0.3 |
| Legionella pneumophila str. Corby | Gammaproteobacteria | - | 38.5% | 3204 | 2.4% | -1.8 |
| Legionella pneumophila str. Lens | Gammaproteobacteria | - | 38.4% | 2934 | 2.6% | -1.3 |
| Legionella pneumophila str. Paris | Gammaproteobacteria | - | 38.3% | 3165 | 2.6% | -1.6 |
| Legionella pneumophila subsp. pneumophila str. Philadelphia 1 | Gammaproteobacteria | - | 38.3% | 2942 | 2.6% | -1.0 |
| Mannheimia succiniciproducens MBEL55E | Gammaproteobacteria | - | 42.5% | 2380 | 1.6% | -1.1 |
| Marinobacter aquaeolei VT8 | Gammaproteobacteria | - | 56.9% | 4272 | 2.8% | 5.6 |
| Marinomonas sp. MWYL1 | Gammaproteobacteria | - | 42.6% | 4438 | 4.0% | 7.3 |
| Methylococcus capsulatus str. Bath | Gammaproteobacteria | - | 63.6% | 2959 | 2.1% | 2.9 |
| Pasteurella multocida subsp. multocida str. Pm70 | Gammaproteobacteria | - | 40.4% | 2015 | 2.4% | 1.7 |
| Photobacterium profundum SS9 | Gammaproteobacteria | - | 41.7% | 5491 | 4.6% | 6.5 |
| Photorhabdus luminescens subsp. laumondii TTO1 | Gammaproteobacteria | + | 42.8% | 4683 | 3.9% | 4.6 |
| Pseudoalteromonas atlantica T6c | Gammaproteobacteria | - | 44.6% | 4281 | 5.3% | 9.3 |
| Pseudoalteromonas haloplanktis TAC125 | Gammaproteobacteria | - | 40.1% | 3483 | 3.9% | 1.4 |
| Pseudomonas aeruginosa PA7 | Gammaproteobacteria | - | 66.4% | 6285 | 2.3% | 6.0 |
| Pseudomonas aeruginosa PAO1 | Gammaproteobacteria | + | 66.6% | 5567 | 2.1% | 5.1 |
| Pseudomonas aeruginosa UCBPP-PA14 | Gammaproteobacteria | + | 66.3% | 5891 | 2.3% | 4.5 |
| Pseudomonas fluorescens Pf0-1 | Gammaproteobacteria | - | 60.5% | 5735 | 3.0% | 4.0 |
| Pseudomonas fluorescens Pf-5 | Gammaproteobacteria | - | 63.3% | 6135 | 3.2% | 5.9 |
| Pseudomonas mendocina ymp | Gammaproteobacteria | + | 64.7% | 4593 | 2.6% | 6.2 |
| Pseudomonas putida F1 | Gammaproteobacteria | - | 61.9% | 5250 | 3.5% | 8.6 |
| Pseudomonas putida GB-1 | Gammaproteobacteria | - | 61.9% | 5409 | 2.9% | 5.7 |
| Pseudomonas putida KT2440 | Gammaproteobacteria | - | 61.5% | 5349 | 3.3% | 8.3 |
| Pseudomonas putida W619 | Gammaproteobacteria | - | 61.4% | 5181 | 3.0% | 6.8 |
| Pseudomonas stutzeri A1501 | Gammaproteobacteria | - | 63.9% | 4128 | 2.8% | 5.7 |
| Pseudomonas syringae pv. phaseolicola 1448A | Gammaproteobacteria | + | 57.9% | 5169 | 3.8% | 7.2 |
| Pseudomonas syringae pv. syringae B728a | Gammaproteobacteria | + | 59.2% | 5088 | 3.8% | 6.7 |
| Pseudomonas syringae pv. tomato str. DC3000 | Gammaproteobacteria | + | 58.3% | 5607 | 3.7% | 6.3 |
| Psychrobacter arcticus 273-4 | Gammaproteobacteria | - | 42.8% | 2120 | 10.8% | 17.9 |
| Psychrobacter sp. PRwf-1 | Gammaproteobacteria | - | 44.8% | 2385 | 12.2% | 21.6 |
| Psychromonas ingrahamii 37 | Gammaproteobacteria | - | 40.1% | 3541 | 3.3% | 4.0 |
| Saccharophagus degradans 2-40 | Gammaproteobacteria | - | 45.8% | 4008 | 3.5% | 0.4 |
| Salmonella enterica subsp. arizonae serovar 62:z4.z23:-- | Gammaproteobacteria | + | 51.4% | 4510 | 2.8% | 1.7 |
| Salmonella enterica subsp. enterica serovar Agona str. SL483 | Gammaproteobacteria | + | 52.0% | 4614 | 2.7% | 1.0 |
| Salmonella enterica subsp. enterica serovar Choleraesuis str. SC-B67 | Gammaproteobacteria | + | 52.1% | 4659 | 2.8% | 0.9 |
| Salmonella enterica subsp. enterica serovar Dublin str. CT_02021853 | Gammaproteobacteria | + | 52.1% | 4617 | 2.8% | 1.1 |
| Salmonella enterica subsp. enterica serovar Enteritidis str. P125109 | Gammaproteobacteria | + | 52.2% | 4205 | 2.8% | 0.8 |
| Salmonella enterica subsp. enterica serovar Gallinarum str. 287/91 | Gammaproteobacteria | + | 52.2% | 3964 | 2.6% | 0.8 |
| Salmonella enterica subsp. enterica serovar Heidelberg str. SL476 | Gammaproteobacteria | + | 52.1% | 4779 | 2.5% | 0.2 |
| Salmonella enterica subsp. enterica serovar Newport str. SL254 | Gammaproteobacteria | + | 52.2% | 4805 | 2.6% | 1.1 |
| Salmonella enterica subsp. enterica serovar Paratyphi A str. ATCC 9150 | Gammaproteobacteria | + | 52.2% | 4091 | 2.7% | 1.0 |
| Salmonella enterica subsp. enterica serovar Paratyphi B str. SPB7 | Gammaproteobacteria | + | 52.1% | 5601 | 3.1% | 2.5 |
| Salmonella enterica subsp. enterica serovar Schwarzengrund str. CVM19633 | Gammaproteobacteria | + | 52.2% | 4627 | 2.8% | 1.9 |
| Salmonella enterica subsp. enterica serovar Typhi str. CT18 | Gammaproteobacteria | + | 51.9% | 4753 | 2.9% | 1.9 |
| Salmonella enterica subsp. enterica serovar Typhi str. Ty2 | Gammaproteobacteria | + | 52.1% | 4312 | 2.7% | 0.8 |
| Salmonella enterica subsp. enterica serovar Typhimurium str. LT2 | Gammaproteobacteria | + | 52.2% | 4523 | 2.8% | 1.8 |
| Shewanella amazonensis SB2B | Gammaproteobacteria | - | 53.6% | 3645 | 3.0% | 5.0 |
| Shewanella baltica OS155 | Gammaproteobacteria | + | 46.2% | 4489 | 4.7% | 7.6 |
| Shewanella baltica OS185 | Gammaproteobacteria | - | 46.3% | 4394 | 4.7% | 7.2 |
| Shewanella baltica OS195 | Gammaproteobacteria | + | 46.2% | 4687 | 4.8% | 7.7 |
| Shewanella baltica OS223 | Gammaproteobacteria | - | 46.3% | 4440 | 4.8% | 7.3 |
| Shewanella denitrificans OS217 | Gammaproteobacteria | - | 45.1% | 3754 | 4.9% | 5.0 |
| Shewanella frigidimarina NCIMB 400 | Gammaproteobacteria | - | 41.6% | 4029 | 4.5% | 3.5 |
| Shewanella halifaxensis HAW-EB4 | Gammaproteobacteria | - | 44.6% | 4278 | 4.2% | 6.0 |
| Shewanella loihica PV-4 | Gammaproteobacteria | - | 53.7% | 3859 | 3.4% | 4.8 |
| Shewanella oneidensis MR-1 | Gammaproteobacteria | - | 45.9% | 4472 | 4.0% | 5.1 |
| Shewanella pealeana ATCC 700345 | Gammaproteobacteria | - | 44.7% | 4241 | 4.1% | 5.2 |
| Shewanella piezotolerans WP3 | Gammaproteobacteria | - | 43.3% | 4933 | 3.9% | 3.3 |
| Shewanella putrefaciens CN-32 | Gammaproteobacteria | - | 44.5% | 3972 | 4.4% | 6.4 |
| Shewanella sediminis HAW-EB3 | Gammaproteobacteria | - | 46.1% | 4497 | 3.6% | 4.7 |
| Shewanella sp. ANA-3 | Gammaproteobacteria | - | 47.9% | 4360 | 4.5% | 7.0 |
| Shewanella sp. MR-4 | Gammaproteobacteria | - | 47.9% | 3924 | 5.0% | 9.3 |
| Shewanella sp. MR-7 | Gammaproteobacteria | - | 47.9% | 4014 | 5.0% | 8.5 |
| Shewanella sp. W3-18-1 | Gammaproteobacteria | - | 44.6% | 4044 | 4.5% | 6.4 |
| Shewanella woodyi ATCC 51908 | Gammaproteobacteria | - | 43.7% | 4880 | 3.1% | 0.7 |
| Shigella boydii CDC 3083-94 | Gammaproteobacteria | + | 51.0% | 4556 | 2.6% | -3.7 |
| Shigella boydii Sb227 | Gammaproteobacteria | - | 51.1% | 4281 | 2.5% | -6.0 |
| Shigella dysenteriae Sd197 | Gammaproteobacteria | + | 51.0% | 4494 | 5.7% | 14.8 |
| Shigella flexneri 2a str. 2457T | Gammaproteobacteria | - | 50.9% | 4063 | 2.5% | -0.6 |
| Shigella flexneri 2a str. 301 | Gammaproteobacteria | + | 50.7% | 4436 | 2.7% | 0.0 |
| Shigella flexneri 5 str. 8401 | Gammaproteobacteria | - | 50.9% | 4116 | 3.0% | -1.7 |
| Shigella sonnei Ss046 | Gammaproteobacteria | + | 50.8% | 4456 | 2.8% | 1.1 |
| Sodalis glossinidius str. 'morsitans' | Gammaproteobacteria | + | 54.5% | 2516 | 2.6% | 1.2 |
| Stenotrophomonas maltophilia K279a | Gammaproteobacteria | - | 66.3% | 4386 | 3.5% | 6.1 |
| Stenotrophomonas maltophilia R551-3 | Gammaproteobacteria | - | 66.3% | 4039 | 2.7% | 1.8 |
| Thiomicrospira crunogena XCL-2 | Gammaproteobacteria | - | 43.1% | 2196 | 3.4% | 2.4 |
| Vibrio cholerae O395 | Gammaproteobacteria | - | 47.5% | 3875 | 3.2% | 2.5 |
| Vibrio fischeri ES114 | Gammaproteobacteria | - | 38.3% | 3801 | 3.5% | 2.1 |
| Vibrio fischeri MJ11 | Gammaproteobacteria | - | 38.2% | 4039 | 3.1% | 1.0 |
| Vibrio harveyi ATCC BAA-1116 | Gammaproteobacteria | + | 45.4% | 6054 | 2.9% | 0.7 |
| Vibrio splendidus LGP32 | Gammaproteobacteria | - | 43.9% | 4425 | 3.5% | 3.2 |
| Vibrio vulnificus CMCP6 | Gammaproteobacteria | - | 46.7% | 4487 | 3.2% | 2.6 |
| Vibrio vulnificus YJ016 | Gammaproteobacteria | - | 46.7% | 5024 | 3.4% | 4.0 |
| Wigglesworthia glossinidia endosymbiont of Glossina brevipalpis | Gammaproteobacteria | - | 22.5% | 611 | 1.0% | -2.7 |
| Xanthomonas axonopodis pv. citri str. 306 | Gammaproteobacteria | + | 64.7% | 4427 | 4.3% | 6.2 |
| Xanthomonas campestris pv. campestris str. 8004 | Gammaproteobacteria | + | 65.0% | 4273 | 4.0% | 5.8 |
| Xanthomonas campestris pv. campestris str. ATCC 33913 | Gammaproteobacteria | + | 65.1% | 4180 | 4.1% | 6.3 |
| Xanthomonas campestris pv. vesicatoria str. 85-10 | Gammaproteobacteria | + | 64.6% | 4487 | 4.5% | 8.2 |
| Xanthomonas oryzae pv. oryzae KACC10331 | Gammaproteobacteria | + | 63.7% | 4080 | 5.2% | 11.4 |
| Xanthomonas oryzae pv. oryzae MAFF 311018 | Gammaproteobacteria | + | 63.7% | 4372 | 3.9% | 6.5 |
| Xanthomonas oryzae pv. oryzae PXO99A | Gammaproteobacteria | + | 63.6% | 4988 | 3.3% | 2.1 |
| Xylella fastidiosa 9a5c | Gammaproteobacteria | - | 52.6% | 2832 | 6.0% | 2.8 |
| Xylella fastidiosa M12 | Gammaproteobacteria | - | 51.9% | 2104 | 5.5% | 4.2 |
| Xylella fastidiosa M23 | Gammaproteobacteria | - | 51.7% | 2201 | 4.5% | 3.2 |
| Yersinia enterocolitica subsp. enterocolitica 8081 | Gammaproteobacteria | + | 47.2% | 4050 | 3.6% | 2.5 |
| Yersinia pestis Angola | Gammaproteobacteria | + | 47.6% | 4044 | 3.8% | 3.8 |
| Yersinia pestis Antiqua | Gammaproteobacteria | + | 47.7% | 4363 | 3.9% | 3.8 |
| Yersinia pestis CO92 | Gammaproteobacteria | + | 47.6% | 4062 | 4.0% | 4.2 |
| Yersinia pestis KIM | Gammaproteobacteria | + | 47.7% | 4216 | 4.3% | 3.8 |
| Yersinia pestis Nepal516 | Gammaproteobacteria | + | 47.6% | 4094 | 3.9% | 3.2 |
| Yersinia pestis Pestoides F | Gammaproteobacteria | + | 47.7% | 4069 | 3.8% | 3.0 |
| Yersinia pseudotuberculosis IP 31758 | Gammaproteobacteria | + | 47.2% | 4321 | 4.0% | 3.2 |
| Yersinia pseudotuberculosis IP 32953 | Gammaproteobacteria | + | 47.6% | 4035 | 4.3% | 4.1 |
| Yersinia pseudotuberculosis PB1/+ | Gammaproteobacteria | + | 47.5% | 4237 | 4.2% | 3.6 |
| Yersinia pseudotuberculosis YPIII | Gammaproteobacteria | + | 47.5% | 4192 | 4.1% | 3.4 |
| Acidithiobacillus ferrooxidans ATCC 53993 | Other Bacteria | - | 58.9% | 2826 | 2.0% | -1.9 |
| Akkermansia muciniphila ATCC BAA-835 | Other Bacteria | - | 55.8% | 2138 | 2.7% | 1.0 |
| Dictyoglomus thermophilum H-6-12 | Other Bacteria | - | 33.7% | 1912 | 0.3% | -3.3 |
| Magnetococcus sp. MC-1 | Other Bacteria | - | 54.2% | 3716 | 3.6% | 2.6 |
| Syntrophomonas wolfei subsp. wolfei str. Goettingen | Other Bacteria | - | 44.9% | 2504 | 0.9% | -2.6 |
| Thermodesulfovibrio yellowstonii DSM 11347 | Other Bacteria | - | 34.1% | 2033 | 0.4% | -2.8 |
| Rhodopirellula baltica SH 1 | Planctomycetes | - | 55.4% | 7325 | 8.6% | 15.0 |
| Borrelia afzelii PKo | Spirochaetes | - | 27.8% | 1133 | 1.2% | -1.8 |
| Borrelia recurrentis A1 | Spirochaetes | - | 27.5% | 990 | 1.1% | -1.3 |
| Leptospira biflexa serovar Patoc strain 'Patoc 1 (Ames)' | Spirochaetes | - | 38.9% | 3600 | 1.6% | -3.5 |
| Leptospira biflexa serovar Patoc strain 'Patoc 1 (Paris)' | Spirochaetes | - | 38.9% | 3390 | 1.9% | -2.1 |
| Leptospira borgpetersenii serovar Hardjo-bovis JB197 | Spirochaetes | - | 40.2% | 2880 | 1.7% | -0.7 |
| Leptospira borgpetersenii serovar Hardjo-bovis L550 | Spirochaetes | - | 40.2% | 2945 | 1.6% | -1.1 |
| Treponema pallidum subsp. pallidum str. Nichols | Spirochaetes | - | 52.7% | 1036 | 1.0% | -2.5 |
| Fervidobacterium nodosum Rt17-B1 | Thermotogae | - | 35.0% | 1750 | 1.5% | 0.7 |
| Petrotoga mobilis SJ95 | Thermotogae | - | 34.1% | 1898 | 0.6% | -2.6 |
| Thermosipho africanus TCF52B | Thermotogae | - | 30.8% | 1911 | 0.3% | -3.1 |
| Thermosipho melanesiensis BI429 | Thermotogae | - | 31.4% | 1879 | 0.4% | -2.0 |
| Thermotoga lettingae TMO | Thermotogae | - | 38.7% | 2040 | 0.6% | -1.9 |
| Thermotoga maritima MSB8 | Thermotogae | - | 46.2% | 1858 | 0.4% | -1.4 |
| Thermotoga petrophila RKU-1 | Thermotogae | - | 46.1% | 1785 | 0.6% | -0.4 |
| Thermotoga sp. RQ2 | Thermotogae | - | 46.2% | 1819 | 0.5% | -1.1 |
