# Supplementary material for: Sequence-Based Prediction of Type III Secreted Proteins
Source: PLoS Pathog. 2009 Apr 24;5(4):e1000376. doi: 10.1371/journal.ppat.1000376 (PMC2669295; doi:10.1371/journal.ppat.1000376)
Supplement: Table S12 — Input features of the machine learning algorithms after initial feature selection. This table comprises these features, which are selected from all possible feature combinations using three different alphabets (amino acid alphabet, amino acid property alphabet, hydrophobic/hydrophilic alphabet) with a maximal pattern length of three. In order to avoid over-fitting on the data, only features are selected which are not specific to either the positive or the negative set but exists in both. (0.07 MB DOC) [file ppat.1000376.s015.doc]

Table S12. Input features of the machine learning algorithms after initial feature selection

This table comprises these features, which are selected from all possible feature combinations using three different alphabets (amino acid alphabet, amino acid property alphabet, hydrophobic/hydrophilic alphabet) with a maximal pattern length of three. In order to avoid over-fitting on the data, only features are selected which are not specific to either the positive or the negative set but exists in both.

| **Amino acid alphabet** |
| --- |
| Alanine |
| Arginine |
| Asparagine |
| Aspartic Acid |
| Glutamic Acid |
| Glutamine |
| Glycine |
| Histidine |
| Isoleucine |
| Leucine |
| Lysine |
| Methionine |
| Phenylalanine |
| Proline |
| Serine |
| Serine-Leucine |
| Serine-Serine |
| Threonine |
| Threonine-Leucine |
| Tyrosine |
| Valine |

| **Hydrophobic/hydrophilic alphabet** |
| --- |
| hydrophilic-hydrophilic |
| hydrophilic-hydrophilic-hydrophilic |
| hydrophilic-hydrophilic-hydrophobic |
| hydrophilic-hydrophobic-hydrophilic |
| hydrophilic-hydrophobic-hydrophobic |
| hydrophobic-hydrophilic-hydrophilic |
| hydrophobic-hydrophilic-hydrophobic |
| hydrophobic-hydrophobic-hydrophilic |

| **Amino acid property alphabet** |
| --- |
| acidic |
| acidic-hydrophobic |
| alkaline |
| alkaline-alkaline |
| alkaline-hydrophilic |
| alkaline-hydrophobic |
| alkaline-hydrophobic-hydrophobic |
| alkaline-hydrophobic-polar |
| alkaline-polar |
| alkaline-polar-polar |
| aromatic |
| hydrophilic |
| hydrophilic-alkaline |
| hydrophilic-hydrophobic |
| hydrophilic-polar |
| hydrophobic |
| hydrophobic-acidic |
| hydrophobic-alkaline |
| hydrophobic-alkaline-hydrophobic |
| hydrophobic-alkaline-polar |
| hydrophobic-hydrophilic |
| hydrophobic-hydrophobic |
| hydrophobic-hydrophobic-hydrophobic |
| hydrophobic-hydrophobic-polar |
| hydrophobic-ionizable |
| hydrophobic-polar |
| hydrophobic-polar-hydrophobic |
| hydrophobic-polar-polar |
| ionizable |
| ionizable-polar |
| polar |
| polar-acidic |
| polar-alkaline |
| polar-alkaline-hydrophobic |
| polar-alkaline-polar |
| polar-hydrophilic |
| polar-hydrophilic-hydrophobic |
| polar-hydrophobic-hydrophobic |
| polar-hydrophobic-polar |
| polar-polar |
| polar-polar-alkaline |
| polar-polar-hydrophobic |
| polar-polar-polar |
